# Methods in Public Health Environmental Justice Research: a Scoping Review from 2018 to 2021

**DOI:** 10.1007/s40572-023-00406-7

**Published:** 2023-08-15

**Authors:** Joan A. Casey, Misbath Daouda, Ryan S. Babadi, Vivian Do, Nina M. Flores, Isa Berzansky, David J.X. González, Yoshira Ornelas Van Horne, Tamarra James-Todd

**Affiliations:** 1 University of Washington School of Public Health, Seattle, WA USA; 2 Columbia University Mailman School of Public Health, New York, NY USA; 3 Department of Environmental Health, Harvard T.H. Chan School of Public Health, Boston, USA; 4 Department of Environmental Science, Policy & Management and School of Public Health, University of California, Berkeley, Berkeley, CA 94720 USA

**Keywords:** Vulnerable populations, Environmental justice, Racism, Health status disparities, Socioeconomic factors, Environmental exposure

## Abstract

**Purpose of Review:**

The volume of public health environmental justice (EJ) research produced by academic institutions increased through 2022. However, the methods used for evaluating EJ in exposure science and epidemiologic studies have not been catalogued. Here, we completed a scoping review of EJ studies published in 19 environmental science and epidemiologic journals from 2018 to 2021 to summarize research types, frameworks, and methods.

**Recent Findings:**

We identified 402 articles that included populations with health disparities as a part of EJ research question and met other inclusion criteria. Most studies (60%) evaluated EJ questions related to socioeconomic status (SES) or race/ethnicity. EJ studies took place in 69 countries, led by the US (*n* = 246 [61%]). Only 50% of studies explicitly described a theoretical EJ framework in the background, methods, or discussion and just 10% explicitly stated a framework in all three sections. Among exposure studies, the most common area-level exposure was air pollution (40%), whereas chemicals predominated personal exposure studies (35%). Overall, the most common method used for exposure-only EJ analyses was main effect regression modeling (50%); for epidemiologic studies the most common method was effect modification (58%), where an analysis evaluated a health disparity variable as an effect modifier.

**Summary:**

Based on the results of this scoping review, current methods in public health EJ studies could be bolstered by integrating expertise from other fields (e.g., sociology), conducting community-based participatory research and intervention studies, and using more rigorous, theory-based, and solution-oriented statistical research methods.

**Supplementary Information:**

The online version contains supplementary material available at 10.1007/s40572-023-00406-7.

## Introduction

Environmental risk factors account for approximately one quarter of global deaths, with a higher burden among children under 5 and those in low- and middle-income countries (LMICs) [1]. In addition, a consistent social gradient has been observed where persistently marginalized and disadvantaged communities bear disproportionately high environmental exposures compared to more advantaged groups [2–4]. These environmental health disparities are produced by multi-level factors, including discriminatory practices at global, national, regional, and local levels in the distribution of neighborhood resources and environmental hazards to internal dose and individual psychosocial stress response [5]. Correspondingly, addressing such disparities will likely require multi-level policy interventions [6]. Heightened awareness of environmental health disparities in the United States (US) in recent years has pushed some policymakers and organizations to create working groups, funding opportunities, and policies aimed at identifying and mitigating environmental injustices.

The US Environmental Protection Agency (EPA) and the White House EJ Advisory Council (WHEJAC) define environmental justice (EJ) based on “fair [just] treatment and meaningful involvement of all people” in environmental law, regulation, and policy development, implementation, enforcement, practices, and activities related to human health and the environment” [7]. During his first week in office, President Joe Biden issued an executive order on the climate crisis that established the Justice40 Initiative, which directs 40% of benefits from many federal investments to disadvantaged communities [8]. The order also created the federal Office of Environmental Justice. In 2022, the US EPA established an office of Environmental Justice and External Civil Rights and signed a 5-year Memorandum of Understanding with the World Health Organization to cooperate on EJ issues [7, 9]. The 2018–2023 National Institutes of Environmental Health Sciences (NIEHS) Strategic Plan supported research in EJ and environmental health disparities across all priorities [10]. Though this broad interest in EJ research is more recent, the field has benefitted from years of contributions (e.g., definitions, frameworks, and statistical methods) from scholars across multiple disciplines (e.g., sociology, history, geography, epidemiology, environmental health sciences) that can support further work.

Environmental injustice can be defined by focusing on the relationships (commensal, symbiotic, parasitic) between different groups as “the avoidance of hazards and acquisition of benefits [by certain, often more privileged groups] through relationships that negatively impact the environment of [other groups]” [11]. This frame describes the processes through which disproportionate exposures arose rather than describing them as attributes of a disadvantaged community. As an example of such relationships, in the US, Dr. Robert Bullard conducted the first highly publicized EJ study in 1983, finding that toxic waste facilities in Houston, Texas, were disproportionately sited in predominately Black communities [12]. Bullard cited racial discrimination, especially in the housing market, zoning, and decisions by public officials as the major drivers of the observed disparity. Since the 1980s, several authors have offered frameworks to conduct EJ research within. Examples include Gee and Payne-Sturges (2004) [13], Morello-Frosch and Lopez (2006) [14], Corburn (2017) [15], Van Horne et al. (2022) [16], Kreger et al. (2011) [17], Jones 2001 [18], and Bailey et al. 2021 [19] .These frameworks provided the backbone (either explicitly or implicitly) for an explosion of environmental justice research from 2010–2020.

Demonstrating this rise in EJ scholarship, a PubMed search of “environmental justice” found an average of 10 EJ articles published per year from 1992 to 2006, compared to 77 per year over the next 15-year period (2007–2022). In the context of environmental health sciences, EJ research typically seeks to determine the distribution of environmental exposures across different groups or within a disadvantaged population and to evaluate if disproportionate exposure is linked to adverse health effects. In general, this means an EJ study will consider the intersection of social disadvantage with environmental factors for the ultimate goal of achieving health equity. Studies may focus on exposure and sociodemographics alone (i.e., exposure science) or include both exposure and health outcomes (i.e., epidemiologic research) [2, 20–23]. Studies have evaluated different types of environmental justice including distributive environmental injustice or the disproportionate exposure among certain disadvantaged groups. Some studies evaluate procedural or participatory injustice, or exclusion of certain groups from the decision-making process about policies that result in environmental exposure disparities [24]. Environmental injustice occurs globally and most research has been conducted in high-income countries, but this research trend has begun to shift [25, 26].

In this scoping review, we focused on the research process and asked the questions: what were the goals of the environmental justice studies and which methods did researchers use to achieve these goals? Prior reviews have covered specific methods and topics related to EJ, including: methods for EJ air pollution studies [27, 28] and proximity-based studies [29]; participatory EJ research [30]; fine-scale spatio-temporal [31] and remote sensing [32] data for EJ; causal inference methods for EJ [33]; and methods for longitudinal EJ studies [34]. However, to our knowledge, there has not been a comprehensive review of EJ methods as they have been applied across the diverse subdisciplines of environmental health sciences.

Here, we surveyed the scholarly literature published between January 2018 and December 2021. Our aim was to critically compare the diverse approaches to EJ research in the environmental health sciences and to make recommendations for approaches to designing effective, policy-relevant, and solution-oriented EJ research. Through this review, we seek to highlight commonly used methods and statistical approaches, as well as identify gaps and strategies for moving the field of EJ research forward.

## Methods

**Fig. 1:**
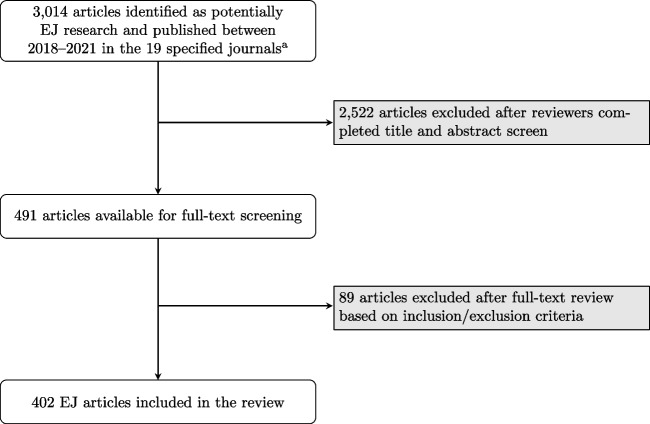
Flowchart of article identification and inclusion in the review. EJ, environmental justice. ^a^See Table 1 for the list of a priori specified journals

We searched PubMed for articles related to EJ published between January 2018 and December 2021 in 19 environmental science and epidemiology journals determined a priori by author team consensus (Table 1). Our goal was to provide an overview of EJ methods in the environmental health sciences and therefore our inclusion criteria allowed for articles that may not have explicitly self-declared as conducting EJ research. This approach allowed us to capture articles that would still fit EJ research within the context of environmental health sciences (e.g., research that investigates the distribution of environmental exposures across different groups or within a marginalized population). We imported all identified references into Covidence (*N* = 3014), an online tool for screening and extracting data for reviews [35], and our team of 10 reviewers assessed each for inclusion using the following steps [35]. First, five reviewers screened the titles and abstracts of 3014 articles ( ~603 articles each), identifying 2522 articles as unrelated to our topic of interest (Fig. 1) based on a set of criteria determined as a study team (Table 2). Next, we conducted a full-text review of the remaining 491 articles to determine inclusion/exclusion based on the same criteria used for title/abstract screening. For the full-text review, each article was screened by two reviewers with disagreements settled via discussion between the two reviewers. Our final sample included 402 articles (13% of the initial search).

**Table 1:** Search strategy for the scoping review on methods for EJ research

Journals searched	Inclusion search terms	Exclusion search terms
1. American Journal of Epidemiology 2. American Journal of Public Health 3. Environment International 4. Environmental Epidemiology 5. Environmental Health 6. Environmental Health Perspectives 7. Environmental Justice 8. Environmental Research 9. Environmental Research Letters 10. Environmental Science & Technology 11. Epidemiology 12. Health and Place 13. International Journal of Environmental Research and Public Health 14. International Journal of Epidemiology 15. International Journal of Hygiene and Environmental Health 16. Journal of Epidemiology and Community Health 17. Journal of Exposure Science and Environmental Epidemiology 18. Journal of Occupational and Environmental Medicine 19. Science of the Total Environment	(Health Inequities OR Health Disparities OR Race Factors OR Minority Health OR Sociology OR Prejudice OR Social Discrimination OR Social Isolation OR Stereotyping OR Redlin* OR race/ethnicity[tiab] OR sexuality OR gender identity) AND (Environmental Justice OR Environmental Health OR Environmental Pollution OR Metals OR Particulate Matter OR Nitrogen Dioxide OR Ozone) AND (2018:2021[pdat])	NOT (“review”[Publication Type])

**Table 2:** Inclusion/exclusion criteria for review

Inclusion (studies included if all 4 criteria met)	Exclusion (studies excluded if one criterion met)
1. Peer-reviewed empirical studies published as formal articles 2. Published January 2018–December 2021 3. Study directly assessed an environmental factor^a^ as the outcome or main exposure of interest 4. Research question incorporated a population experiencing a health disparity^b^ as the study population, a predictor of the environmental exposure, or as a mediator or modifier in analyses	1. Reviews, commentaries, meeting abstracts, dissertations 2. Occupational epidemiology studies 3. Animal studies 4. Studies of primary drug consumption (e.g., cigarettes, vaping, hookah, pharmaceuticals, etc.) 5. Studies of sugar-sweetened beverages 6. Health impact assessments 7. Simulation/modeling studies

Factors included on the NIEHS Environmental Agents list at https://www.niehs.nih.gov/health/topics/agents/index.cfm; we added factors including noise and traffic based on reviewer discretion

^b^Population experiencing a health disparity defined based on the National Institute on Minority Health and Health Disparities’ definition at: https://www.nimhd.nih.gov/about/strategic-plan/nih-strategic-plan-definitions-and-parameters.html; we added populations experiencing a health disparity to more effectively characterize non-US populations based on consensus of two reviewers assigned to each study

We then used the Covidence platform to extract information from each article (one reviewer per article, see Supplemental Methods 1). This information included the relevant populations experiencing a health disparity, environmental factor(s), method(s) used for EJ analysis, EJ-specific finding(s), subjective study quality assessment (below average, average, top 25%, top 10%), and information on studies providing a framework or theory for their research. Populations experiencing a health disparity were defined by six non-mutually exclusive categories: global health, race/ethnicity, SES, sexual/gender minority, underserved rural, or underserved urban [36]. Global health studies were those conducted in LMIC, but we attempted to exclude studies in high-income areas of LMIC (e.g., Cape Town, South Africa). We searched the introduction, methods, and discussion for mention of a framework and subjectively categorized each study section as either explicitly stating a framework, providing some description of a framework (but not adequate), or not describing a framework. To qualify as “explicitly stating a framework,” the section of the paper had to discuss a specific theory or framework (see examples in introduction) or provide a comprehensive summary of upstream factors linked to the relation studied. A study section with “some description of a framework, not adequate” may have cited prior EJ literature and provided 1–2 sentences of text, and a study section with “no description of framework” may have simply stated, for example, “we stratified analyses by race/ethnicity.” We characterized the methods used by study authors to conduct EJ analyses (often a subset of the entire analyses). Following the review of the published articles, we broadly categorized methods as descriptive, qualitative, main effect regression modeling (hereafter, “regression”), effect modification, and mediation. We surveyed methods used for spatial analysis, causal inference, and EJ-specific summary measures (e.g., use of EPA’s EJScreen). Post hoc, we extracted the affiliation and location of each paper’s corresponding author and compared this to the study location country.

## Results

### Overall Summary and Article Distribution by Journal

**Fig. 2:**
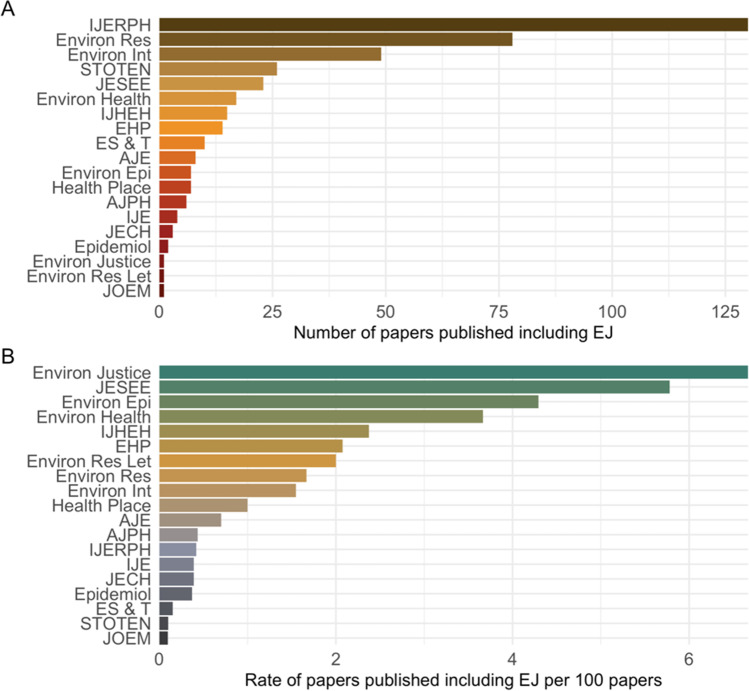
The number of articles identified as including environmental justice (EJ) in 19 environmental health journals from 2018 to 2021. Displayed as **A** absolute count of articles including EJ and **B** rate of articles including EJ per 100 articles published at the journal. AJE, American Journal of Epidemiology; AJPH, American Journal of Public Health; Environ Int, Environment International; Environ Epi, Environmental Epidemiology; Environ Health, Environmental Health; EHP, Environmental Health Perspectives; Environ Justice; Environmental Justice; Environ Res, Environmental Research; Environ Res Let, Environmental Research Letters; Epidemiol, Epidemiology; ES & T, Environmental Science & Technology; IJERPH, International Journal of Environmental Research and Public Health; IJE, International Journal of Epidemiology; IJHEH, International Journal of Hygiene and Environmental Health; JESEE, Journal of Environmental Science and Environmental Epidemiology; JECH, Journal of Epidemiology and Community Health; JOEM, Journal of Occupational and Environmental; Medicine STOTEN, Science of the Total Environment

This scoping review on methods used for EJ research included 402 articles published in 19 environmental health and epidemiology journals between January 2018 and December 2021. Fifteen (4%) of these articles appeared online in 2021 but had final publication dates of 2022. The average number of published EJ studies increased throughout the study period from 5 per month in 2018 to 9 per month in 2021. Among the journals we assessed, the *International Journal of Environmental Research and Public Health* (*IJERPH*) was the largest publisher of environmental health EJ articles (*N* = 127 [32%]), followed by *Environmental Research* (*N* = 77 [19%]), and *Environment International* (*N* = 49 [12%]) (Fig. 2A). We also examined the proportion of articles published in each journal that examined EJ issues in an environmental health framework. *Environmental Justice* published the highest rate of articles (6.7 per 100), followed by the *Journal of Exposure Science and Environmental Epidemiology* (*JESEE*) (5.8 per 100) and *Environmental Epidemiology* (4.3 per 100). The proportion was comparatively low for *IJERPH*, which published 0.4 EJ articles per 100 (Fig. 2B).

### Populations Experiencing a Health Disparity and Study Locations

In order to be included, articles could either (a) evaluate exposure science or epidemiologic questions in a population experiencing a health disparity [36] (e.g., chemical exposures among Black women [37]); or (b) consider a population experiencing a health disparity as an outcome, predictor, effect modifier, or mediator in the main analysis (e.g., the association between ambient temperature and stillbirth stratified by maternal race/ethnicity [38]).

**Fig. 3:**
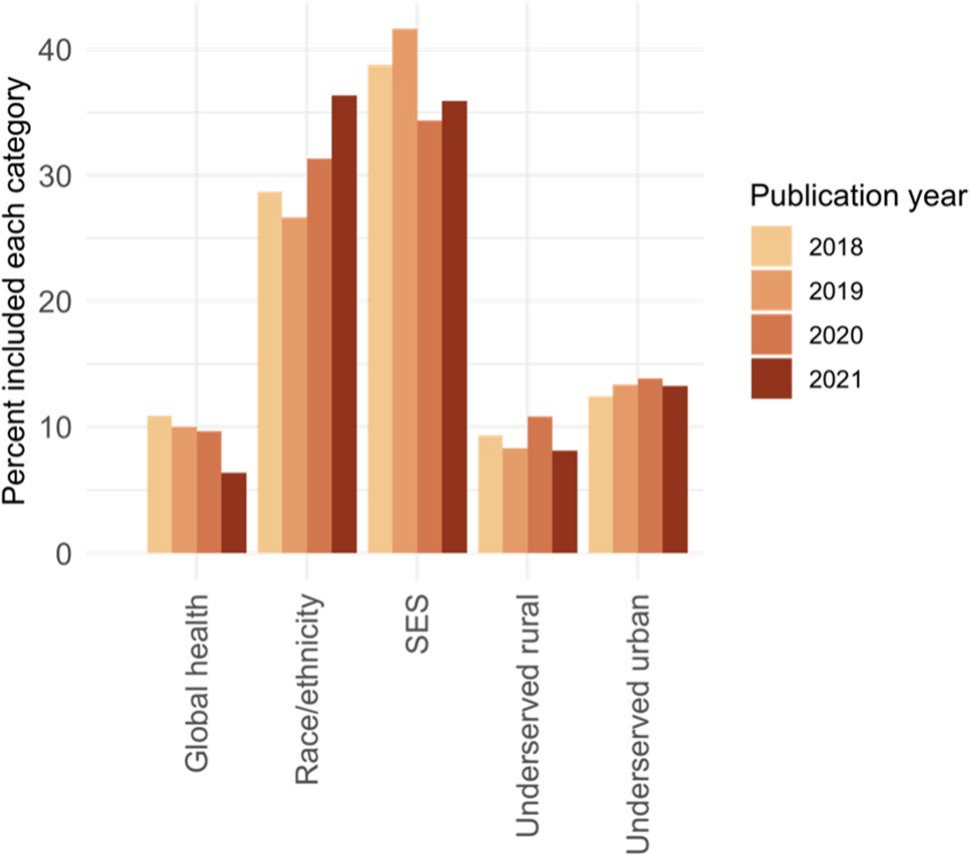
Categories of populations experiencing a health disparity by which articles qualified as environmental justice studies for inclusion in the review. Studies could qualify via multiple categories and the figure categories are not mutually exclusive. SES, socioeconomic status

Articles could qualify by including multiple populations experiencing a health disparity (e.g., SES and underserved rural). The most common way articles qualified was by studying race/ethnicity and SES together (*N* = 83 [21%]); however, many evaluated SES alone (*N* = 80 [20%]) or race/ethnicity alone (*N* = 77 [19%]) (Fig. S1). The most popular categories were race/ethnicity and SES across all study publication years (Fig. 3). None of the EJ studies identified considered gender or sexual minorities. About 15% (*N* = 63) were classified as global health studies, with a total number of 19 general global health, 16 studying underserved rural populations, 14 studying underserved urban populations, and 14 evaluating SES factors. The percent of global health EJ studies decreased between 2018 (11%) and 2021 (6%). We observed the opposite trend for studies including race/ethnicity, which grew from 29% in 2018 to 36% in 2021; this trend was consistent among exposure-only and epidemiologic studies (Fig. S2).

Researchers characterized populations with health disparities in many ways, with some providing few and others providing more details that acknowledged the population as a marginalized group. However, a description of the population experiencing a health disparity was not always explicitly stated. High quality studies described populations experiencing health disparities in detail by doing at least one of the following: (a) providing schematics/conceptual frameworks to guide the reader as to the linkages between environmental and social variables [39, 40]; (b) incorporating a strong theoretical underpinning for the chosen question/population pairing or comparison [41, 42]; (c) including historical context as to the relevance of the selected population experiencing a health disparity [43, 44]; (d) explicitly stating the relevance and meaning of proxy variables as they related to particular constructs [45, 46] (e.g., voter turnout as a proxy for access to resources to mobilize political change [47]); and (e) describing who measured the health disparity population variables [48, 49] (e.g., self-reported SES or race/ethnicity). Some researchers used participant race/ethnicity as a proxy for (unmeasured) differences in allele frequencies [50, 51] or only provided biological explanations for differential associations observed by participant race/ethnicity [52, 53]. As discussed below, these practices are not recommended.

**Fig. 4:**
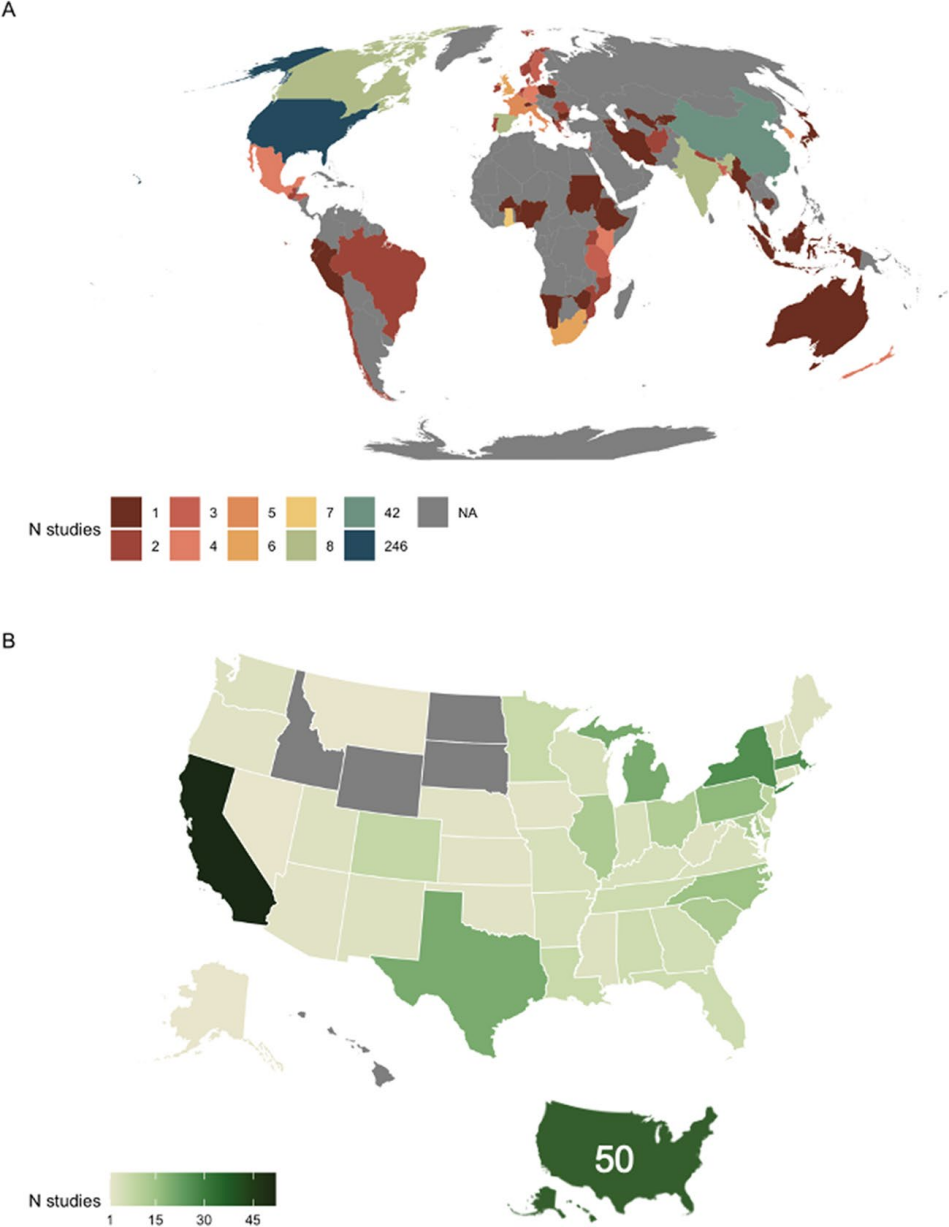
Locations where EJ studies were conducted, 2018–2021. **A** Spatial distribution of EJ studies globally. If studies were conducted in multiple countries, they contributed counts to each country’s total. **B** Spatial distribution of EJ studies within the US. Fifty studies were conducted nationwide (often just continental US) or were nationally representative (dark green nationwide count)

Studies used sociodemographic and health data spanning 1940–2021 across diverse settings (Fig. 4). EJ studies took place in 69 countries, led by the US (*N* = 246 [61%]), China (*N* = 42 [10%]), and Canada, India, and Spain (each 8 [2%]). Researchers at 254 unique institutions (as measured by corresponding author affiliation) conducted the 402 EJ studies included in this review (Table S2). Just 4 institutions (measured by corresponding author affiliation) conducted 10 or more EJ studies: Harvard University (*n* = 20); Columbia University (*n* = 16); UC Berkeley (*n* = 10); University of Michigan (*n* = 10). When looking at all the EJ studies during the review time period, the percent of studies conducted in the US increased, from 52% (*N* = 39) in 2018 to 72% (*N* = 94) in 2021. Within the US, the most studies were conducted in California (*N* = 52 [21%]), Massachusetts and New York (each 26 [11%]), and Michigan and Texas (each 20 [8%]). Nationwide studies or nationally representative samples were also common (*N* = 50 [20%]). Seven studies were conducted among Indigenous peoples [43, 54–59], including in Navajo Nation [54, 55], Akwesasne Mohawk Nation [56], and the Crow Tribe [57]. For 37% (*n* = 58) of non-US studies (*n* = 157), the corresponding author’s institution was in a different country than the study site (Figures S3–S4). In US-based studies (*n* = 245), nearly all corresponding authors had an affiliation with an institution located in the US.

### EJ Frameworks

**Fig. 5:**
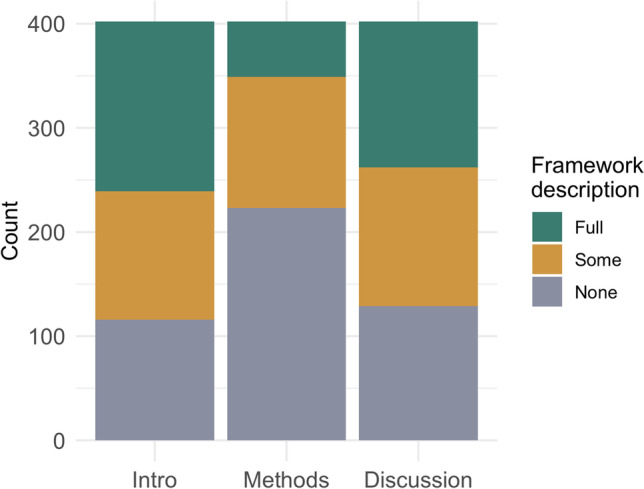
Level of EJ framework description by article section. For a study to qualify as providing a “full” description of a framework, the section of the paper had to discuss a specific theory or framework (see examples in introduction) or provide a comprehensive summary of upstream factors linked to the relation studied. A study with “some (inadequate)” description of a framework may have cited prior EJ literature and provided 1–2 sentences of text, and a study with “no” description of framework may have simply stated, for example, “we stratified analyses by race/ethnicity”

We summarized whether studies described an EJ framework in the introduction, methods, or discussion section in three levels: no discussion, some discussion (inadequate), or explicit (full) discussion. Often, authors did not describe an EJ framework in any section of the published paper (*N* = 76 [19%], Fig. 5). In 200 studies (50%), the authors did explicitly describe a framework in one of the three sections, and in 42 studies (10%) they explicitly stated a framework in all three sections. Authors explicitly discussed an EJ framework in 40% of introduction sections but only in 13% of methods sections and 35% of discussion sections. Over 50% of methods sections made no mention of an EJ framework.

Differences in framework use emerged when comparing exposure-only and epidemiology studies. In area-level exposure-only studies, use of a framework was common. 71% (*N* = 67) explicitly stated a framework in the introduction, methods, or discussion and 24% (*N* = 23) explicitly stated framework in all sections. Far fewer studies that evaluated personal exposure included a framework in any section (38%; *N* = 43) and only 11 (10%; [41, 60–69]) explicitly stated one in all sections. Epidemiology studies included an EJ framework at a similar prevalence of personal-exposure studies. Less than half (46% [*N* = 90]) of epidemiology studies included an EJ framework in any section and just 4% (*N* = 8; [43, 45, 48, 70–74]) included one in all sections. Methods sections generally lacked an EJ framework, with only 15 (8%) epidemiology studies [42, 43, 45, 48, 70–80] explicitly stating one there.

### Methods Used to Assess EJ Questions

We summarized study attributes and methods by two study types: environmental exposure-only and epidemiologic. We classified exposures as area-level (e.g., neighborhood) and personal-level (e.g., participant address or biospecimen) and summarized the exposures evaluated. We also characterized the methods used to conduct EJ analyses in 5 mutually exclusive categories based on common methods used in exposure science and epidemiologic research, as well as what was seen in these published articles: descriptive, qualitative methods, regression (main effect regression), effect modification, and mediation. We also touch on topics such as spatial analysis, causal inference methods, and measures somewhat unique to EJ studies.

#### Exposure-Only Study Attributes

**Fig. 6:**
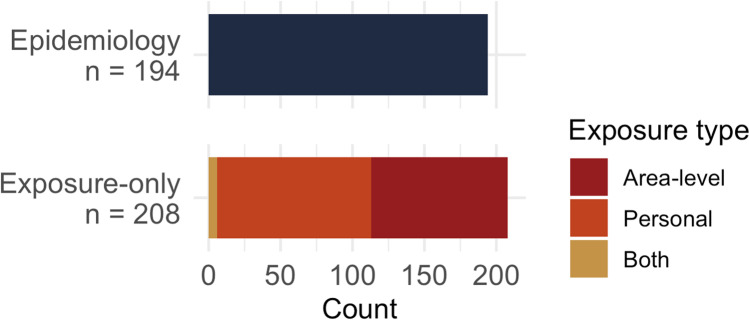
Total number of epidemiology and exposure-only studies included with exposure measurement type among exposure-only studies

About half (52%; *n* = 208) of included studies focused only on exposure, for example, examining the racial/ethnic composition of block groups located near and farther from natural gas flaring [81] or the effectiveness of biomass stoves for improving indoor air quality by individual-level SES [82]. Among exposure-only studies, 46% (*n* = 95) used area-level measures alone, 51% (*n* = 107) focused on personal exposure, and 3% (*n* = 6) studies used both area-level and individual-level measures of exposure (Fig. 6). For subsequent summaries, we group personal exposure studies with the six studies that evaluated both personal and area-level exposures.

Personal exposure assessment relied on modeled environmental data linked to residential locations [83–86], wearable monitors [87–89], questionnaires [90–92], biomarkers of exposure from biospecimens [93–96], and in-home environmental data collection [66, 97–100]. Area-level exposure-only studies used satellite imagery [101–103], emissions data [104], environmental sampling [39, 55, 105, 106], mobile monitoring [107], exposure modeling [108], surveys [109], and administrative databases [47, 110, 111]. These area-level studies primarily evaluated air pollution (40%), water pollution (9%), ambient temperature (9%), and greenspace (8%). Personal exposure studies considered a different set of environmental factors, dominated by chemicals (35%) but also including metals (19%), air pollution (19%), and environmental tobacco smoke (12%). The full list of exposures evaluated by study type appears in Table 3.

**Table 3:** Environmental exposures evaluated in exposure-only studies

	Exposure type	
Ranking	Area-level (%; [example citations]) *N* = 94 studies	Personal^a^ (%; [example citations]) *N* = 111 studies
1	Air pollution (40%; [84, 104, 112–115])	Chemicals (35%; [56, 93, 94, 116, 117])
2	Ambient temperature (9%; [103, 107, 118, 119]) Water pollution (9%; [55, 103, 105, 106])	Metals (19%; [66, 67, 120, 121])
3	Greenspace (8%; [44, 102, 122])	Air pollution (19%; [60, 82, 123, 124])
4	Climate events (5%; [125–127]) Industrial facilities (5%; [128–130]) Metals (5%; [131–133])	Environmental tobacco smoke (12%; [134–136])
5	Cumulative environmental exposures (4%; [39, 137])	WASH (7%; [97, 138, 139])
6	WASH (3%; [109, 110])	Housing environmental quality (3%; [140–142])
7	Noise pollution (2%; [108, 111]) Oil and gas infrastructure (2%; [47, 81]) Pesticides (2%; [108, 143]) Soil contamination (2%; [144, 145])	Greenspace (2%; [65, 146])
8	Light pollution (1%; [101])	Climate events (1%; [147]) Cumulative environmental exposures (1%; [148]) Noise pollution (1%; [83]) Oil and gas infrastructure (1%; [68]) Pesticides (1%; [149])

WASH, water, sanitation, and hygiene

^a^Studies (*n* = 6) that included both personal and area-level are included in the personal exposure column

Considering the studied population experiencing a health disparity, 34 (16%) exposure-only studies were conducted in a LMIC and focused on air pollution (*N* = 16 [47%]), water, sanitation, and hygiene (WASH, *n* = 9 [26%]), and ambient temperature (*n* = 3 [9%]). Most other exposure-only studies considered race/ethnicity and/or SES (*n* = 123) and their environmental exposures are represented in Table 3.

#### Exposure-Only Study Statistical Methods

**Fig. 7:**
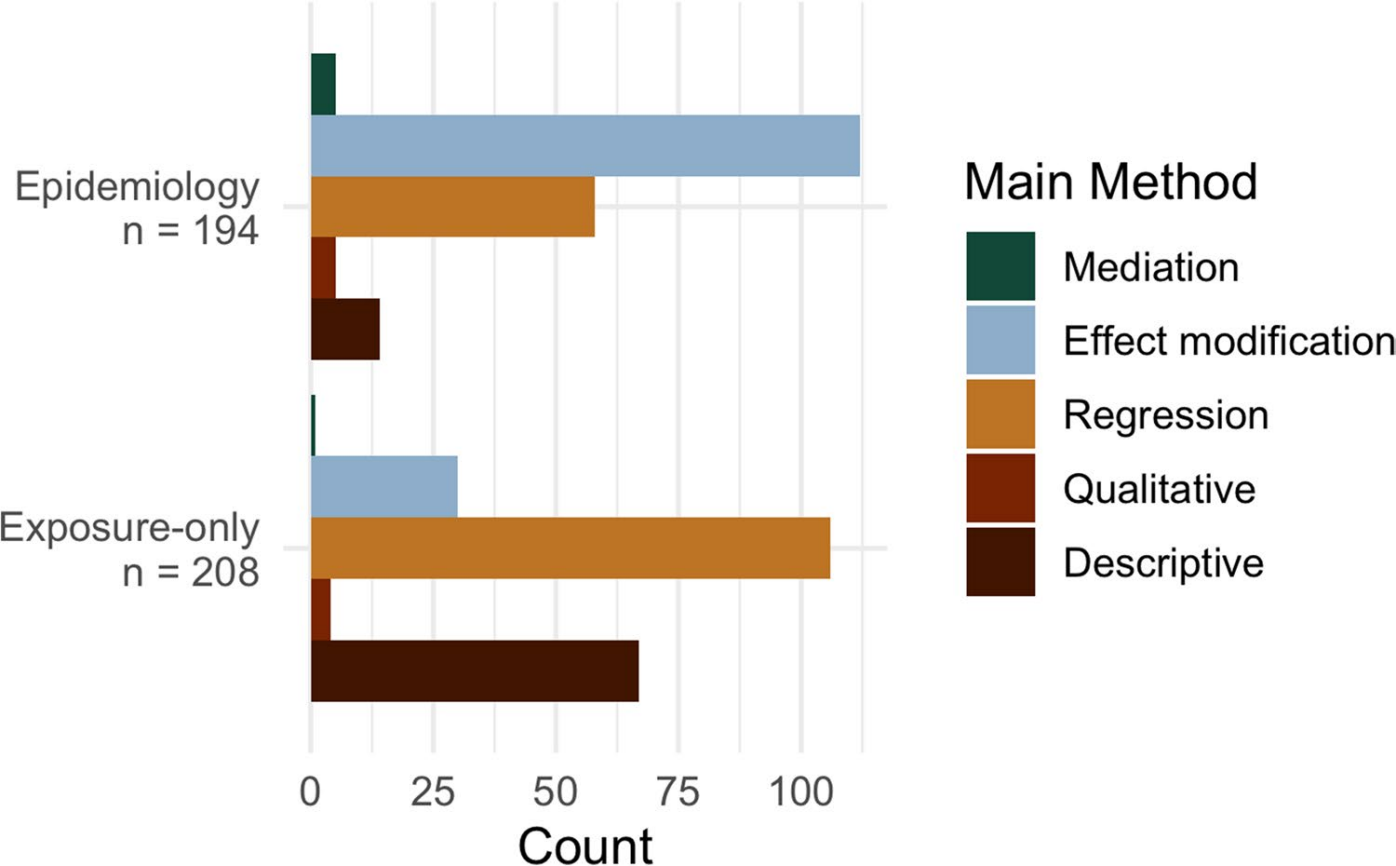
Main method used for EJ analyses by epidemiology and exposure-only study types

The majority (*n* = 105 [50%]) of exposure-only studies used main effect regression to evaluate the EJ question of interest (Fig. 7, Table S3). Examples of this include studies assessing the association between individual income and residential air pollution exposure [60], the association between individual race/ethnicity and volatile organic compound metabolite levels [150], or the association between block group level racial/ethnic composition and income and ambient temperature [118]. Descriptive analyses were the next most common (32%) and included analyses such as calculating the prevalence of environmental tobacco smoke exposure by the racial/ethnic identity of the birthing person [151] or the correlation between neighborhood educational attainment and noise levels [83]. Thirty (14%) exposure-only studies used effect modification methods, either stratification or statistical interaction to test for differential relationships by the health disparity population. Examples include stratifying the overall model evaluating the association between moving to a greener neighborhood and change in physical activity by area-based income [152], or as Buck Louis et al. [153] included, an interaction term between birthing person race/ethnicity and chemical plasma concentration in a model testing the main effect between chemical concentration and neonatal anthropometric measurements. Authors rarely used qualitative (*n* = 4 [2%] [41, 57, 65, 154]) or mediation (*n* = 1 [< 1%] [64]) methods.

In a sub-analysis, we compared the methods used in the most common area-level (air pollution; *n* = 38) and personal-exposure (chemicals; *n* = 38) studies (Fig. S5). Main effect regression was the most common EJ method in both study types (50% for air pollution and 61% for chemicals). Differences also emerged. No area-level air pollution studies used mediation methods and no personal chemical studies used qualitative methods as their main EJ method. Descriptive statistics were common for area-level air pollution studies (45%) and effect modification was common for personal chemical studies (18%). Personal chemical exposure studies that used effect modification were representative of the body of exposure studies that evaluated effect modification by a health disparity metric and virtually all stratified main models or included one or more interaction terms without additional rationale in the methods section (e.g., [153, 155–158]). For additional description of methods used in air pollution EJ studies, please see a recent review by Gardner-Frolick et al. [27].

We now turn to community-based participatory research (CBPR) or community-engaged methods. The levels of community involvement used for strengthening research efforts vary from involvement only in the recruitment of participants to full integration as partners in the research process of exposure and environmental epidemiological studies [159]. In this scoping review, we identified limited exposure studies utilizing CBPR or community-engaged methods. Only 10 of the 208 (3%) exposure studies in this scoping review acknowledged using a CBPR approach [57, 66, 67, 130, 145, 154, 160–163]. Among these 10 studies, seven [57, 66, 67, 145, 154, 160, 161] discussed the extensive community and partnership building process typical of CBPR studies. The main exposure medium of interest among exposure studies was water, with four studies focusing on this pathway [57, 66, 67, 163], followed by two studies focusing on air pollution [154, 161] and two on chemicals [160, 162]. While qualitative approaches were employed by some exposure studies utilizing CBPR [57, 161], most relied on non-parametric methods such as Spearman correlations and Kruskal-Wallis test [66, 130, 145, 160]. Additionally, multivariate mixed effects regression models and advanced risk assessment modeling methods were also key statistical methods used [67, 161, 163]. One of the standout CBPR exposure studies was a project based in Richmond, CA that was conducted in collaboration with the Center for Environmental Research and Children’s Health (CERCH) and RYSE, a youth air quality justice organization [161]. Together, they not only identified disparities in NO_2_, with census tracts with higher Black populations being disproportionally affected, but also identified that higher levels of greenspace were associated with lower NO_2_. The study employed univariate and linear regression methods for the air quality analysis and contextualized the findings by engaging the youth in workshops and photovoice. Their recommendations extended beyond a call for more research, as they interpreted findings of greenspace being associated with lower NO_2_ as a call to action to plant more trees.

#### Epidemiologic Studies

About half (*n* = 194 [48%]) of the EJ studies included were epidemiologic, evaluating associations between environmental exposures and health outcomes. These studies considered a range of environmental factors as the primary exposure of interest, including ambient air pollution (*N* = 74 [38%]), chemicals (*n* = 24 [12%]), ambient temperature (*N* = 19 [10%]), and greenspace (*n* = 17 [9%]) (Table 4). Authors considered diverse health outcomes (Table 4); adverse birth outcomes (*n* = 37 [19%]), mortality (*n* = 27 [14%]), cardiovascular disease (CVD) risk factors (e.g., type 2 diabetes, hypertension; *n* = 22 [11%]), and respiratory disease (*n* = 18 [9%]) were most prevalent. The most common exposure-outcome combinations were: air pollution and mortality (*N* = 15 [46, 164–177]); chemicals and developmental outcomes (*N* = 7 [178–184]); and ambient temperature and mortality [185–189] and adverse birth outcomes [38, 190–193] (both *N* = 5). Twenty-nine studies were conducted in LMIC countries and like exposure-only studies primarily focused on air pollution exposures (*N* = 19 [45%]).

**Table 4:** Environmental exposures and health outcomes evaluated in epidemiologic studies

Exposures (%; [example citations])	Health outcomes^a^ (%; [example citations])
Air pollution (38%; [76, 170, 194–196])	Adverse birth outcomes (19%; [45, 102, 197–199])
Chemicals (12%; [181, 184, 200–202])	Mortality (14%; [46, 165, 170, 174, 188, 189])
Ambient temperature (10%; [193, 203, 204])	Cardiometabolic (10%; [49, 205–208])
Greenspace (9%; [74, 207, 209])	Respiratory (10%; [210–214])
Metals (8%; [40, 215, 216])	Developmental (9%; [178, 182, 183])
Multiple exposures (4%; [43, 217])	Mental health and violence-related (7%; [194, 218, 219])
Pesticides (3%; [79, 220])	Reproductive (6%; [221–223])
Housing environmental quality (3%; [214, 224])	Immunological (5%; [72, 225, 226])
Built environment (2%; [77, 227]) Climate event (2%; [228, 229]) Industrial facilities (2%; [230, 231])	Endocrine (5%; [37, 232]) Multiple^b^ (5%; [233, 234])
WASH (2%; [235, 236])	Cancer (3%; [237, 238])
Cumulative environmental exposures (1%; [80]) Noise pollution (1%; [239]) Pollen (1%; [240]) Water pollution (1%; [241])	Bodyweight (3%; [202, 242]) Other^c^ (3%; [243, 244])
Blue space (0.5%; [245]) Oil and gas infrastructure (0.5%; [73]) Environmental tobacco smoke (0.5%; [246])	Neurological (2%; [247, 248])
	Renal (1%; [249]) Hepatic (1%; [216])

WASH, water, sanitation, and hygiene

^a^Categories based on those previously used by the National Academies: http://nap.nationalacademies.org/26156

^b^Multiple outcomes included studies that evaluated more than two categories, such as bodyweight, cardiometabolic, and respiratory.

^c^Other included 5 studies that considered dental health and perceived health or quality of life

#### Epidemiologic Study Statistical Methods

Most epidemiologic studies qualified as EJ research by evaluating effect modification by a health disparity factor (*N* = 112 [58%]) (Fig. 7, Table S3). These studies stratified their main models by the health disparity factor or included an interaction of the health disparity factor and the environmental exposure of interest. For example, Fong et al. evaluated the association between residential greenspace at the time of birth and adverse birth outcomes stratified by individual SES and found stronger associations for birthing people with higher education attainment for term low birth weight and small for gestational age birth [250]. Many studies assessed effect modification without explanation, often stating in the methods section, “we tested for effect modification by stratifying models by a third variable” (e.g., race/ethnicity, SES) that indicated a population experiencing a health disparity. Some studies provided appropriate context and explained such testing. For example, Niehoff et al. evaluated effect modification by race/ethnicity of the overall association between toenail metal concentrations and risk of breast cancer and devoted a paragraph in the introduction to explaining why, stating that racial/ethnic minorities may live closer to industrial facilities and experience additional environmental and social stressors (including racism) that could amplify the effect of metal exposure [251].

Main effect regression was the next most common method (*N* = 58 [30%]) used for EJ analyses in epidemiologic studies, where studies evaluated associations between exposures and outcomes exclusively in health disparities populations. Chevier et al. [200] assessed the association between maternal serum and urine insecticide concentrations and adverse birth outcomes in a rural population in South Africa, which met our population experiencing a health disparity definition. In the US, Nozadi et al. evaluated prenatal exposure to metals among Navajo Nation pregnant people and childhood development [54]. In comparison to exposure-only studies, a much smaller percent of epidemiologic studies used descriptive methods (*N* = 14 (7%) vs. 32% for exposure-only studies). For example, James-Todd et al. identified hair products commonly used among 359 Black women in New York City and identified a high prevalence of hormonal activity among these products [37]. Five epidemiologic studies used qualitative methods [43, 214, 231, 243, 244], spanning topics from urban flooding in Detroit [244], industrial mining in Burkina Faso, Mozambique, and Tanzania [231], and environmental and reproductive justice on the Gulf Coast of the US [43]. Finally, five epidemiologic studies used formal mediation analyses to determine if environmental factors mediated the observed relationship between health disparity factors (e.g., poverty) and adverse health outcomes [42, 207, 252–254]. In Bangladesh, Huang et al. considered serum metals as a mediator between childhood marriage and preterm birth, finding that elevated levels of zinc, arsenic, strontium, and barium appeared to mediate the association between childhood marriage and preterm birth [252]. In the US, Song et al. used the Multi-ethnic Study of Atherosclerosis (MESA) and found that ambient PM_2.5_ exposure mediated the association between participant race/ethnicity and higher systolic blood pressure, especially in men [254].

As in exposure-only studies, only 10 of 194 environmental epidemiology studies incorporated community-based participatory or community engagement methods [43, 73, 224, 231, 234, 243, 244, 255–257]. Four [43, 231, 243, 244] employed a qualitative approach (e.g., focus group, interviews), with the other six relying on regression methods [73, 234, 255–257] and latent profile analysis combined with generalized estimating equations [224]. Six studies were conducted in the US [43, 73, 224, 243, 244, 255] and 4 in global settings: Lithuania [256]; Burkina Faso, Mozambique, and Tanzania [231]; Honduras [257]; and Peru [234]. The main exposure of interest among epidemiological studies utilizing community-based methods were air pollution [255, 257], greenspace [224, 256], and fossil fuel production facilities [73, 231]. Respiratory health [73, 224] and cardiovascular health outcomes [255–257] were the most studied outcomes for community-based environmental epidemiology studies. A majority of the studies discussed relying on partners or community members for participant recruitment, with only a few highlighting the utilization of CBPR methods through the whole design of the study. A key research to action environmental epidemiology article was led by Johnston (2021) [73], where academic researchers partnered with Esperanza Community Housing to train *Promotores de Salud* (community health workers) and recruit 961 residents near oil fields in Los Angeles County, USA. Utilizing generalized linear models, the team identified that distance to oil field was associated with lower lung function. Their research has now been cited as key scientific findings during public testimonies, reports, and organizing, which have collectively contributed to the banning of new oil and gas drilling and the phase out of existing operations in the Los Angeles County, USA [258].

#### Additional Methods in Exposure and Epidemiologic Studies

##### Causal Inference Methods

In some instances, causal inference methods may assist researchers in identifying the potential causes for the observed associations [33]. In this review, only one exposure study used causal inference methods [87]. Chillrud et al. estimated changes in indoor air pollution using a difference-in-differences analysis after a randomized cookstove intervention in Ghana, finding improved air quality with use of a liquefied petroleum gas stove, but levels still exceeding recommended limits. More epidemiologic studies employed causal methods. In China, Han et al. used difference-in-differences analysis and found a stronger association between PM_2.5_ concentration and all-cause mortality in low SES (low literacy, college attendance, gross domestic product) vs. higher SES counties [167]. With marginal structural models and inverse-probability-of-treatment weights, Chevier et al. found insecticides were related to accelerated fetal growth in girls [200]. In a final example, Iyer et al. used causal mediation methods to understand how Black-white prostate cancer disparities would change if they fixed greenness exposure to the 75th percentile experienced by white men [42].

##### Spatial Statistics

Many EJ studies involve the use of spatial data, including exposure, outcome, or measure of disadvantage. One issue that arises in regression modeling is residual spatial autocorrelation, or non-independence of error terms. This can impact confidence interval coverage. Methods for handling this data ranged from ignoring it to a range of spatial models. In their paper on neighborhood racial composition, concentrated disadvantage, and air pollution, Liévanos used Moran’s *I* to test residuals for spatial autocorrelation and then applied a spatial lag model [259]. Other exposure studies used spatial error or spatial lag models (e.g., [102, 115, 260, 261]). Others treated the residual correlation as a nuisance, for example, including unstructured [262] and spatially structured [227] random intercepts or using generalized estimating equations [127]. Wheeler et al. included a random intercept at the census tract level when evaluating the association between neighborhood deprivation and blood lead levels in Maryland [262].

A second class of spatial methods sought to identify clusters or “hot spots.” Elford et al. used Moran’s *I* local indicators of spatial autocorrelation (LISA) to identify areas in Toronto with high ultrafine particulate matter exposure and low income, high percentages of immigrants, high government transfer income dependence, and low education rates [114]. Chakraborty similarly applied the local bivariate Moran's I statistic to first identify counties with high hazardous air pollution burdens and high COVID-19 incidence rates and then compared sociodemographics among the high-high counties, finding higher percentages of Black residents and other socially vulnerable groups [78].

##### Metrics for EJ Research

Much of this review focused on environmental exposures and health outcomes. Equally important for EJ research are measures of social factors. In this final section, we describe several perhaps less-familiar concepts often used in EJ research: EJ screening tools, segregation, and gentrification.

Several studies used EJ screening tools [70, 80, 263–267], which identify communities facing dual environmental and socioeconomic burdens based on a numerical summary of many spatiotemporal variables. Examples of such indices include California’s CalEnviroScreen [268], the Whitehouse Council on Environmental Quality’s Climate and Environmental Justice Screening Tool [269], and the US EPA’s EJScreen [270]. In California, Mousavi et al. used CalEnviroScreen and found that census tracts with a higher overall score had higher background PM_2.5_ concentrations and also higher 4th of July fireworks-related PM_2.5_ compared to other communities [265]. Rather than use the entire score, many studies evaluated score components [70, 80, 264]. For example, Padula et al. evaluated the association between components of CalEnviroScreen 2.0 and preterm birth, finding a significant association between the pollution burden score [e.g., air and water pollution] and increased preterm birth risk (other components include population characteristics [e.g., prevalence of children], environmental effects [e.g., hazardous waste], and social factors [e.g., poverty]) [70]. Reliance on score components speak to some of the disadvantages of full scores: interpretability, weighting, and intervention. While useful for identifying EJ communities to allocate government spending, full EJ scores make assumptions about how to weigh different components (e.g., should air pollution count the same as poverty and the same as hazardous waste sites?). Full scores also beg the question: *what drives the association*? Therefore, many researchers opt to focus on score components. Existing EJ screening tools also may not fit every circumstance, and different tools may prioritize different marginalized populations [271]. For example, Zhao et al. built an updated EJ tool for Allegheny County, PA based on EJScreen which that also included labor market access [267].

Racial residential and economic segregation and gentrification also emerged as key social factors in EJ research [71, 125, 237, 272–274]. Segregation—spatial social inequality—has been linked to worse environmental quality via concentration of power in the hands of a few, increased commute distances and increased traffic-related air pollution, limited diffusion of green technology, and erosion of trust and cooperation that undermines environmental protection [275]. Gentrification, where low SES communities experience investment and an influx of higher SES residents [276], may occur disproportionately in communities with higher baseline environmental quality or possibly result in improved environmental quality [277]. Ekinga et al. used an index of Black isolation [278] in St. Louis, MO, and in spatial analyses found that census tracts with the highest levels of Black racial isolation compared to low racial/economic isolation were more likely to be located in air toxic hotspots [237]. In Barcelona, Zayas-Costa et al. evaluated gentrification as an effect modifier of the relationship between greenways and reduced depression/anxiety [274]. They found that the relationship was confined to gentrifiable communities (i.e., low-income communities not yet gentrifying) and was not present in gentrifying or wealthy communities [274]. A related and emerging topic is “climate gentrification,” where disadvantaged communities are being displaced by green climate infrastructure [279, 280]. Aune et al. identified this phenomenon in New Orleans after Hurricane Katrina where higher ground elevation census tracts were more likely to gentrify between 2000 to 2015 [125].

## Discussion

In this scoping review, we found that two major types of studies—exposure science and environmental epidemiology—were represented in environmental health EJ research. In assessing a number of metrics for rigor of these studies, we found that authors of area-level exposure science studies more commonly stated a theoretical EJ framework in the background, methods, or discussion compared to individual-level exposure science or epidemiology studies. Across study types, frameworks infrequently appeared in method sections. The most common analytic tool used across all studies was the evaluation of differences between population subgroups by assessing effect measure modification. For this, few studies provided rationale for this method, or goal of the analysis, beyond simple documentation of difference. Exposure science studies more commonly evaluated health disparities-related variables as predictors of exposure, with some rationale based in environmental frameworks compared to epidemiologic studies. Overall, solution-oriented methods, including intervention-based studies and CBPR studies were less commonly employed, as were statistical methods that evaluated interventions (real or hypothetical), using approaches such as g-computation. Utilizing solution-oriented methods may provide more interpretable and actionable evidence for policymakers and affected communities.

Given the variability in the rigor of studies and the need to move toward more solution-oriented approaches for achieving health equity, we provide several recommendations for improving EJ environmental health:

*Recommendation #1: Recognize that EJ, as an evolving field, confronts diverse and intersecting structural problems, which requires the careful contextualized application of the best available theory and methods.* Environmental health researchers are increasingly updating their theories and integrating emerging methods to interrogate socially constructed variables including race, SES, and gender. Poorly applying methods and theories or failing to consider the complexities of these variables across space, time, and cultures can constrain the internal and external validity of EJ work. So can less accurate measurement of key variables, which can lead to issues of selection bias and measurement error. When examining underlying drivers of structural environmental health disparities, it is important to define the rationale for the inclusion of specific variables, particularly those that were socially constructed, just as we do in the investigation of other health determinants. Beyond selecting, collecting, and measuring EJ-related variables, accurately discussing these key variables is essential. Authors can use background and methods sections to explain how the chosen data and study design elements fit an EJ-relevant research question or framework [15–18]. Further, EJ researchers should push themselves to collect novel variables and work with alternative datasets (i.e., look outside the streetlight) that may extend beyond traditional environmental health risk factors.

Relevant constructs, variable selection, and measurement are context-dependent and may vary across and/or within countries. Thus, providing relevant background and discussion is key to contextualizing study variables and design. Reviewed racial/ethnic studies in the US frequently compared non-Hispanic Black and non-Hispanic White populations, a relevant comparison given the US’s longstanding, multi-level disparate treatment of these groups. However, such a comparison may not be as relevant in other countries; for example, in a Netherlands-based study investigating racial/ethnic disparities in road traffic noise exposure and depressed mood, authors opted to compare across Dutch, Moroccan, Turkish, and Surinamese ethnicity, which reflected more the country’s own socio-historic past. In the US, one study about inequitable park access in Miami accounted for the heterogeneity within the broad “Hispanic” racial/ethnic category by incorporating national origin. The authors go on to outline how the unique migration histories (e.g., Cuban Adjustment Act of 1966) across subcategories of “Hispanic” were crucial in informing their research question and variable construct [241]. We identified only 42 (10%) studies that similarly included detailed framework discussions in their introduction, methods, and discussion sections. These authors provide relevant context that shapes why they conducted the study, how they constructed variables, and what the study findings mean. However, many of the reviewed studies lacked the appropriate context, and in 76 (19%) studies, an EJ framework (e.g., [13–19]) was not discussed at all. Future studies should provide relevant context to ground the EJ research questions throughout [51].

The challenge of dealing with multiple levels of data (e.g., individual, community) and intersecting dimensions of marginalization (e.g., misogynoir) and ensuring that we report out our research results and triangulate our research questions to address the multiple factors at play in EJ research will be key to providing rigorous research studies in this field.

*Recommendation #2: Make EJ questions central in environmental health studies and use appropriate methods to answer them.* Many studies relegated EJ questions as a secondary analysis (e.g., stratifying main models by health disparity variable) without providing a rationale for conducting such an analysis. Further, an EJ framework or construct likely includes several analyses due to the presence of multiple pathways and numerous variables (e.g., studies assessing effect modification by SES variables may evaluate the interaction for income and education). Testing several hypotheses or pathways can lead to multiple testing problems. While methods exist for addressing this issue (Bonferroni adjustment or less stringent adjustments like the false discovery rate), as a field we have not fully identified a gold standard for addressing issues of multiple comparison, which may impact EJ studies just as much as other studies leading to error, misinterpretation, or the overlooking of study results. Many fields have begun to shift away from solely relying on arbitrary p-value thresholds or statistical significance to inform scientific, environmental, social, political, or practical importance [242], and EJ researchers too may want to consider different metrics or approaches. Also worth noting, spatial studies may have unique circumstances; for instance, in calculating local indicators of spatial association (LISA), which are often employed in spatial analyses as screens for distributional injustices, the method conducts statistical tests for each spatial unit (e.g., in a US county analysis, there would be 3100+ tests). This can result in spurious findings, and often overidentifies “hot spots.” However, only some studies applied any sort of p-value adjustment (e.g., Bonferroni, false discovery rate) or sensitivity analyses, as recommended [281]. For example, in an analysis of the convergence of COVID-19 and chronic air pollution, the authors did not apply any adjustment and thus over-identified vulnerable counties [77]. Results from such unadjusted analyses may not be as useful or be misleading to policymakers interested in targeted interventions.

*Recommendation #3: Limit the mischaracterization, misspecification, and/or omission of nuanced social constructs such as race, ethnicity, sex, and gender.* EJ studies tend to heavily rely on social constructs to account for historical or contemporary processes, which carries two risks: (1) the use of inappropriately defined constructs can lead to erroneous attribution of disparities to these constructs and (2) the omission of key constructs can prevent identification of root causes. Here, we focus on race and sex/gender to illustrate this recommendation.

Race is commonly used in US-based EJ studies as a proxy for historically racist policies (e.g., redlining, housing segregation), social disadvantage, inequitable processes (e.g., gentrification), and experiences of racism [282]. Not only did we find that authors did not comment on the limitations of race to act as a proxy for these complex processes, but we also found that most studies used race with inaccurate justifications and model misspecifications, typically treating race as a biological feature rather than a socially constructed characteristic [282, 283]. Further, some studies centered Whiteness and othered individuals of other races, with one study using the term “something other than White” when distinguishing between individual study subjects [96]. Inaccurate justifications, model misspecification, and White-centering framing can perpetuate unproductive interpretations and atheoretical discourse [284–288]. Some recommendations for discussing race and other constructs (e.g., SES [237], urbanicity) include clearly (a) justifying the reason for using the variable, (b) describing how the variable was measured/operationalized, (c) outlining the limitations of its use, and (d) discussing mechanisms for variable-driven disparities outside of biological mechanisms (e.g., social determinants of health) [238, 239, 289]. Following such recommendations will be key for providing interpretable EJ studies.

There has recently been attention to the sexual and gender minorities in the context of environmental health and environmental justice, though none of the studies on these topics met our inclusion criteria. Gender, sex, and sexual orientation are interrelated and complex constructs with substantial sociocultural diversity [290]. Individuals may be marginalized due to gender identity or expression that do not match expectations from dominant social groups, due to sex traits that do not conform to the phenotype of a single sex (e.g., intersex individuals), and/or due to sexual orientations that include homosexual, gay, lesbian, bisexual, queer, pansexual, questioning, same gender loving, and Two Spirit [290]. In a 2022 review [291], Goldsmith and Bell discussed community and structural discrimination for sexual and gender minorities not typically considered by environmental health scientists, including denial of access to housing loans and employment opportunities, as well as individual factors such as disproportionate burdens of certain underlying conditions (e.g., HIV and respiratory issues associated with chest binding) and psychosocial stress, all of which could contribute to health disparities. Environmental health researchers, working with relevant communities and subject area experts, can address critical unanswered questions at the intersections of environmental justice and the experiences of sexual and gender minorities.

*Recommendation #4: Obtain expertise from sociology and other fields in the design and implementation of EJ research.* Environmental health is a field that relies on multiple fields ranging from environmental exposure science to specific subdisciplines of medicine, as well as statistics. When considering research questions about environmental justice and health disparities, environmental health must rely on expertise from varying fields including sociology, history, law, urban design, public policy, and implementation science. For instance, a study focusing on the historical determinants of the distribution of traffic-related air pollution would greatly benefit from a historian’s knowledge of the drivers of road infrastructure siting and associated demographic changes. As in many scientific disciplines, training in or inclusion of other fields may be somewhat limited, which may lead to use of less effective study design, data collection, analytic approaches that could ultimately affect the scientific rigor, study quality and impact. Multi- and transdisciplinary EJ work is an opportunity to expand our training of students and postdoctoral fellows to integrate concepts of social determinants, macro- and micro-level processes (e.g., historical, legal, or political processes driving environmental exposures), and appropriate statistical approaches. Training can occur through university coursework, as well as through career development within professional societies and government organizations. Valuing the rigor of training needed to conduct EJ research will bolster the effectiveness of this research moving forward, as well as providing grounding for EJ research as its own course of study rather than a secondary, afterthought, or fringe research area.

*Recommendation #5: Recognize the importance of community-engaged, community-based participatory, and community-relevant research.* The EJ movement has a long history of being led by communities. From the activist in Warren County resisting infiltration of a waste facility [292] to the recent banning of new oil and gas drilling in the Los Angeles Community [293]. While not all EJ studies will incorporate community-engaged practices it is still important to acknowledge the role and value of being in community [294, 295]. Particularly when it comes to advancing meaningful change, participatory research with EJ communities is more likely to result in structural level change [30]. Full integration of community members into the design, implementation, and action level decisions is recommended to advance EJ.

It is important to mention that as recognition of the critical need for community-based research programs has grown so has the need for institutional support. One of the primary mechanisms of support has been through NIEHS funded Research Centers, which requires a Community Engagement Core [296]. Recently, the U.S EPA has announced the availability of over $100 million dollars in support of EJ grants [270]. With this new influx of support, it will be more important than ever for researchers to integrate participatory methods with communities that are working towards achieving environmental justice [297]. In addition, it will be important for the academy to recognize the timelines and publication requirements to conduct community-based research when considering productivity and tenure cases [298].

*Recommendation #6: Utilize more solution-oriented study design and statistical methods to address environmental justice, given the underlying goal of achieving health equity.* With the goal of achieving health equity, EJ research should increasingly seek to design and conduct solution-oriented research [299]. The vast majority of EJ research focuses on identifying disproportionate burdens using methods such as effect measure modification, the most common analytic approach among our reviewed epidemiology studies and the second most common in environmental exposure sciences studies. Yet, this approach only documents differences between populations without necessarily evaluating the reason for the differences. Documenting this difference is necessary, but is not sufficient, if the goal of EJ research is to achieve health equity. With this in mind, our research questions and the statistical methods must become more solution-oriented by taking the next step—identifying drivers of the observed disparities, finding strategies for intervening, and reducing disparities. This will allow us to move from a fixed research approach to a solution-oriented approach to achieve our EJ and health equity goals. Examples of solution-oriented work include the use of g-computation to evaluate how reducing chemical exposures could reduce the risk of chemical-associated health outcomes, such as preterm birth [300]. We must also assess past and present policies as they impact EJ. For example, in some U.S. cities, historical racist policies such as redlining have been found to be associated with higher exposure to environmental hazards and worse health outcomes [301]. When conducting studies on historical policies such as redlining, it is important to additionally design studies that evaluate the related present-day mechanisms on which we could intervene (e.g., green gentrification, current neighborhood racial segregation, presence of health-promoting environmental attributes). Similar processes are present in other settings across the world, though prior research may be limited or absent. Finally, the use of more qualitative or descriptive studies to identify reasons for disparities might be warranted [302, 303], especially when novel risk factors within health disparities populations are less well-known.

*Recommendation #7: Rigorously design and evaluate interventions with a focus on health equity.* Environmental health disparities cannot be addressed without multilevel and structural interventions that center EJ throughout the research and implementation process. As such, the field of implementation science can play a tremendous role in improving health equity; interventions ranging from increased access to greenspace for inner city children, to adoption of cleaner cooking methods by primary cooks in low- and middle-income countries [304], to reducing contaminants levels from drinking water in correctional facilities [305] all have the potential to improve health outcomes for underserved groups. Ensuring that this objective is met requires that researchers actively seek to both identify and dismantle structural sources of inequity [306]. To do so, researchers should apply social determinants of health lenses to key implementation steps including but not limited to the definition of conceptual frameworks and the prioritization of evidence-based practices, programs, or policies [307]. Concretely, this might entail the following considerations: (1) the potential role of socio-economic and contextual factors in modifying barriers and facilitators of adoption is incorporated in the model or (2) the intervention or policy’s effectiveness among populations experiencing inequities [307]. Similarly, it is crucial to acknowledge the role that previously implemented interventions might unintendedly play in maintaining and/or exacerbating environmental health disparities. When such programs are identified, de-implementation (defined as discontinuing or abandoning practices that are not proven to be effective or are potentially harmful) should be considered [307]. Though relatively new as an area of inquiry, de-implementation research offers frameworks targeting the system to halt environmentally inequitable practices to ultimately achieve environmental justice. Finally, dissemination strategies can support the adoption and sustainability of health equity initiatives. This warrants that the framing of messages and the selection of communication channels occurs in authentic partnership with community members.

*Recommendation #8: Expand the scope of EJ research to include Global South populations.* The majority of the environmental justice literature has focused on the US despite researchers documenting environmental inequities in other Global North [308] and in Global South countries [309]. Addressing this research gap requires first, recognizing that it exists, and second, developing systematic approaches that will allow for a context-dependent adaptation of the EJ frameworks, that have been predominantly used in the US, while limiting bias. On the one hand, extending to the Global South a framework developed in the Global North can be a tactical strategy, allowing research to be conducted within an established paradigm. Using accepted concepts, constructs, and methods can allow studies conducted in the Global South to contribute to knowledge production and support advocacy for change more easily [310]. On the other hand, the health disparity variables in places outside of the US can differ widely from those within; this applies to both the types of markers but also to how they intersect with one another. Foreign frameworks that do not center important historical forces (e.g., the legacy of colonialism, genocides) and contemporary dynamics (e.g., transnational waste disposal, globalization, natural resource extraction) may overlook, attenuate, and prevent the identification of context-specific EJ issues as well as restrain creativity [2]. Researchers eager to conduct EJ research in settings where environmental disparities are not currently studied should familiarize themselves with both the sociohistorical processes and modern-day potential drivers of inequities specific to that regions [311]. This requires acquiring sufficient training and experience to analyze and incorporate insights from archival documents, interviews and ethnographies, and longitudinal data [312]. Importantly, the identification of context-specific markers of inequality and discrimination may point to a paucity of data needed to rigorously evaluate alongside environmental burdens. Indeed, absence of adequate data to measure and analyze environmental injustice is a major problem faced by countries across the Global South [313]. While this challenge may complicate the research process, it also represents a tremendous opportunity to work with governmental entities to co-generate comprehensive datasets at the appropriate temporal and spatial resolution. Such collaborations and initial documentations can pave the way for rigorous and meaningful EJ analyses from the Global South in the future. Further, we found that for 37% of non-US studies, the corresponding author institution was not located in the country where the research was carried out. Leadership by local researchers can improve study quality via place-based knowledge, access to data sets, and action after the study concludes. Further work can build an understanding about the working dynamics and effectiveness of multinational teams conducting EJ research in low- and middle-income country settings.

## Conclusion

Environmental health research necessarily involves complex physical and social contexts that are shaped by structural factors, including racism, classism, sexism, ableism, heteronormativity, and discrimination based on religious belief [5]. These factors vary across time and place, necessitating the appropriate application of relevant EJ theory and selection of effective methods.

This scoping review highlighted the types of public health EJ research and analytic methods used from 2018 to 2021, a period of dramatic increase in research studies in this area. We focused the scoping review on exposure science and epidemiologic studies and observed high variability study rigor. Some of the most dramatic differences in studies were whether studies explicitly stated an EJ framework for addressing the research question and whether they used a solution-oriented approach. In addition, studies showed limited use of other disciplines, such as sociology, which could have better informed the EJ-related work. Of importance, CBPR and intervention study design and methods were not commonly used in either exposure science or epidemiologic studies, with somewhat greater use in the latter. Finally, we note the need to improve methods in EJ research to incorporate not only descriptive methodologies, but also solution-driven analytic approaches in both exposure science and epidemiologic studies. Finally, science communication, research translation, and dissemination studies are needed to evaluate the best ways in which we might be able to reduce environmental health disparities. Importantly, theory and methods from EJ should be carefully considered by all researchers across the environmental health sciences given that the definitions of EJ discussed in this review encompass much, if not all, of the work we do as environmental health scientists. Through asking more informed questions based on current and new EJ frameworks, integrating methods that allow for the identification and implementation of solutions, and incorporating more community-based approaches, we can continue pushing EJ research forward toward achieving health equity.

## Supplementary Information

ESM 1

## References

[1] World Health Organization (2022). Preventing disease through healthy environments: a global assessment of the burden of disease from environmental risks.

[2] Agyeman J (2016). Trends and directions in environmental justice: from inequity to everyday life, community, and just sustainabilities. Annu Rev Environ Resour..

[3] Mohai P, Pellow D, Roberts JT (2009). Environmental justice. Annu Rev Environ Resour..

[4] Taylor D (2014). Toxic Communities.

[5] Lett E (2022). Conceptualizing, Contextualizing, and Operationalizing Race in Quantitative Health Sciences Research. Ann Fam Med..

[6] Gee GC, Payne-Sturges DC (2004). Environmental health disparities: a framework integrating psychosocial and environmental concepts. Environ Health Perspect..

[7] US Environmental Protection Agency (2022). Learn about environmental justice.

[8] The White House (2022). Executive order on tackling the climate crisis at home and abroad.

[9] World Health Organization (2022). Agreement recognizes the increased environmental and public health risks from the warming global climate and prioritizes environmental justice.

[10] National Toxicology Program (2018). National Institute of Environmental Health Sciences.

[11] Kelly-Reif K, Wing S (2016). Urban-rural exploitation: an underappreciated dimension of environmental injustice. J Rural Stud..

[12] Bullard RD (1983). Solid Waste Sites and the Black Houston Community*. Sociol Inq..

[13] Gee GC, Payne-Sturges DC (2004). Environmental health disparities: a framework integrating psychosocial and environmental concepts.

[14] Morello-Frosch R, Lopez R (2006). The riskscape and the color line: examining the role of segregation in environmental health disparities. Environ Res..

[15] Corburn J (2017). Concepts for studying urban environmental justice. Curr Environ Health Rep..

[16] Van Horne YO (2022). An applied environmental justice framework for exposure science. J Expo Sci Environ Epidemiol..

[17] Kreger M (2011). Creating an environmental justice framework for policy change in childhood asthma: a grassroots to treetops approach. Am J Public Health.

[18] Jones CP (2000). Levels of racism: a theoretic framework and a gardener's tale. Am J Public Health.

[19] Bailey ZD (2017). Structural racism and health inequities in the USA: evidence and interventions. Lancet..

[20] Davide DF, Alessandra F, Roberto P (2022). Distributive justice in environmental health hazards from industrial contamination: a systematic review of national and near-national assessments of social inequalities. Soc Sci Med..

[21] McGregor D, Whitaker S, Sritharan M (2020). Indigenous environmental justice and sustainability. Curr Opin Environ Sustain..

[22] Kroepsch AC (2019). Environmental justice in unconventional oil and natural gas drilling and production: a critical review and research agenda. Environ Sci Technol..

[23] Brulle RJ, Pellow DN (2006). Environmental justice: human health and environmental inequalities. Annu Rev Public Health.

[24] Walker G (2009). Beyond distribution and proximity: exploring the multiple spatialities of environmental justice. Antipode..

[25] Chakraborty J (2017). Focus on environmental justice: new directions in international research. Environ Res Lett..

[26] Gouveia N (2022). Air Pollution and environmental justice in Latin America: where are we and how can we move forward?. Curr Environ Health Rep..

[27] Gardner-Frolick R, Boyd D, Giang A (2022). Selecting data analytic and modeling methods to support air pollution and environmental justice investigations: a critical review and guidance framework. Environ Sci Technol..

[28] Miao Q (2015). Environmental equity research: review with focus on outdoor air pollution research methods and analytic tools. Arch Environ Occup Health..

[29] Chakraborty J, Maantay JA, Brender JD (2011). Disproportionate proximity to environmental health hazards: methods, models, and measurement. Am J Public Health..

[30] Davis LF, Ramírez-Andreotta MD (2021). Participatory research for environmental justice: a critical interpretive synthesis. Environ Health Perspect..

[31] Park YM, Kwan M-P (2017). Multi-contextual segregation and environmental justice research: Toward fine-scale spatiotemporal approaches. Int J Environ Res Public Health..

[32] Weigand M (2019). Remote sensing in environmental justice research—a review. ISPRS Int J Geo Inf..

[33] Banzhaf HS, Ma L, Timmins C (2019). Environmental justice: establishing causal relationships. Ann Rev Resour Econ..

[34] Mohai P, Saha R (2015). Which came first, people or pollution? A review of theory and evidence from longitudinal environmental justice studies. Environ Res Lett..

[35] Covidence systematic review software. *Melbourne, Australia*: *Veritas Health Innovation*. [cited 2022; Available from: www.covidence.org

[36] National Institute for Minority Health and Health Disparities (2022). *Minority health and health disparities: definitions and parameters*.

[37] James-Todd T (2021). Hormonal activity in commonly used Black hair care products: evaluating hormone disruption as a plausible contribution to health disparities. J Expo Sci Environ Epidemiol..

[38] Rammah A (2019). Temperature, placental abruption and stillbirth. Environ Int..

[39] Yang J (2021). Rethinking of environmental health risks: a systematic approach of physical-social health vulnerability assessment on heavy-metal exposure through soil and vegetables. Int J Environ Res Public Health..

[40] Zilversmit Pao L (2019). The cumulative risk of chemical and nonchemical exposures on birth outcomes in healthy women: the Fetal Growth Study. Int J Environ Res Public Health..

[41] Assefa GM (2021). Gender equality and social inclusion in relation to water, sanitation and hygiene in the Oromia region of Ethiopia. Int J Environ Res Public Health..

[42] Iyer HS (2020). The contribution of residential greenness to mortality among men with prostate cancer: a registry-based cohort study of Black and White men. Environ Epidemiol..

[43] Liddell JL, Kington SG (2021). "Something was attacking them and their reproductive organs": environmental reproductive justice in an Indigenous tribe in the United States Gulf Coast. Int J Environ Res Public Health..

[44] Nardone A (2020). Redlines and greenspace: the relationship between historical redlining and 2010 greenspace across the United States. Environ Health Perspect..

[45] do Nascimento FP, de Almeida MF, Gouveia N (2022). Individual and contextual socioeconomic status as effect modifier in the air pollution-birth outcome association. Sci Total Environ..

[46] Lipfert FW, Wyzga RE (2020). Environmental predictors of survival in a cohort of U.S. military veterans: a multi-level spatio-temporal analysis stratified by race. Environ Res..

[47] Silva GS, Warren JL, Deziel NC (2018). Spatial modeling to identify sociodemographic predictors of hydraulic fracturing wastewater injection wells in Ohio Census Block Groups. Environ Health Perspect..

[48] Tu R (2021). Low socioeconomic status aggravated associations of exposure to mixture of air pollutants with obesity in rural Chinese adults: a cross-sectional study. Environ Res..

[49] Juarez PD (2020). The effects of social, personal, and behavioral risk factors and PM(2.5) on cardio-metabolic disparities in a cohort of community health center patients. Int J Environ Res Public Health..

[50] Chiu YH (2018). Maternal intake of pesticide residues from fruits and vegetables in relation to fetal growth. Environ Int..

[51] Shaffer RM (2019). Maternal urinary phthalate metabolites in relation to gestational diabetes and glucose intolerance during pregnancy. Environ Int..

[52] Parada H (2020). Understanding the relationship between environmental arsenic and prostate cancer aggressiveness among African-American and European-American men in North Carolina. Int J Environ Res Public Health..

[53] Fishe J (2022). Environmental effects on acute exacerbations of respiratory diseases: A real-world big data study. Sci Total Environ..

[54] Nozadi SS (2021). Prenatal metal exposures and infants' developmental outcomes in a Navajo population. Int J Environ Res Public Health..

[55] Credo J (2019). Quantification of elemental contaminants in unregulated water across western Navajo nation. Int J Environ Res Public Health..

[56] Ravenscroft J, Schell LM, Akwesasne E (2018). Task Force on the, Patterns of PCB exposure among Akwesasne adolescents: the role of dietary and inhalation pathways. Environ Int..

[57] Martin C (2021). Our relationship to water and experience of water insecurity among Apsaalooke (Crow Indian) people, Montana. Int J Environ Res Public Health..

[58] Sobel M (2022). Environmental-level exposure to metals and metal-mixtures associated with spirometry-defined lung disease in American Indian adults: evidence from the Strong Heart Study. Environ Res..

[59] Nigra AE (2020). Inequalities in public water arsenic concentrations in counties and community water systems across the United States, 2006-2011. Environ Health Perspect..

[60] Tonne C (2018). Socioeconomic and ethnic inequalities in exposure to air and noise pollution in London. Environ Int..

[61] Yeter D, Banks EC, Aschner M (2020). Disparity in risk factor severity for early childhood blood lead among predominantly African-American black children: the 1999 to 2010 US NHANES. Int J Environ Res Public Health..

[62] Chiofalo JM (2019). Pediatric blood lead levels within New York City public versus private housing, 2003-2017. Am J Public Health..

[63] Nguyen VK (2020). A comprehensive analysis of racial disparities in chemical biomarker concentrations in United States women, 1999-2014. Environ Int..

[64] Moody HA, Grady SC (2021). Lead emissions and population vulnerability in the Detroit Metropolitan Area, 2006-2013: impact of pollution, housing age and neighborhood racial isolation and poverty on blood lead in children. Int J Environ Res Public Health..

[65] Fontan-Vela M (2021). Active use and perceptions of parks as urban assets for physical activity: A mixed-methods study. Health Place..

[66] Odetola L, Sills S, Morrison S (2021). A pilot study on the feasibility of testing residential tap water in North Carolina: implications for environmental justice and health. J Expo Sci Environ Epidemiol..

[67] Eggers MJ (2018). Community engaged cumulative risk assessment of exposure to inorganic well water contaminants, Crow Reservation, Montana. Int J Environ Res Public Health..

[68] Ish J, Symanski E, Whitworth KW (2019). Exploring disparities in maternal residential proximity to unconventional gas development in the Barnett Shale in north Texas. Int J Environ Res Public Health..

[69] Rowles Iii LS (2020). Seasonal contamination of well-water in flood-prone colonias and other unincorporated U.S. communities. Sci Total Environ..

[70] Padula AM (2018). Environmental pollution and social factors as contributors to preterm birth in Fresno County. Environ Health..

[71] Yitshak-Sade M (2020). Estimating the combined effects of natural and built environmental exposures on birthweight among urban residents in Massachusetts. Int J Environ Res Public Health..

[72] Bradatan C (2020). Child health, household environment, temperature and rainfall anomalies in Honduras: a socio-climate data linked analysis. Environ Health..

[73] Johnston JE (2021). Respiratory health, pulmonary function and local engagement in urban communities near oil development. Environ Res..

[74] Runkle JD (2022). Racial and ethnic disparities in pregnancy complications and the protective role of greenspace: A retrospective birth cohort study. Sci Total Environ..

[75] Chevrier J (2019). Sex and poverty modify associations between maternal peripartum concentrations of DDT/E and pyrethroid metabolites and thyroid hormone levels in neonates participating in the VHEMBE study, South Africa. Environ Int..

[76] Riddell CA (2022). Hyper-localized measures of air pollution and risk of preterm birth in Oakland and San Jose, California. Int J Epidemiol..

[77] Awuor L, Melles S (2019). The influence of environmental and health indicators on premature mortality: An empirical analysis of the City of Toronto's 140 neighborhoods. Health Place..

[78] Chakraborty J (2021). Convergence of COVID-19 and chronic air pollution risks: Racial/ethnic and socioeconomic inequities in the U.S. Environ Res..

[79] Orta OR (2021). Brominated flame retardants and organochlorine pesticides and incidence of uterine leiomyomata: a prospective ultrasound study. Environ Epidemiol..

[80] Huang H (2018). Investigation of association between environmental and socioeconomic factors and preterm birth in California. Environ Int..

[81] Johnston JE (2020). Environmental justice dimensions of oil and gas flaring in south Texas: disproportionate exposure among Hispanic communities. Environ Sci Technol..

[82] Grajeda LM (2020). Effectiveness of gas and chimney biomass stoves for reducing household air pollution pregnancy exposure in Guatemala: sociodemographic effect modifiers. Int J Environ Res Public Health..

[83] Thacher JD (2020). High-resolution assessment of road traffic noise exposure in Denmark. Environ. Res..

[84] Rosofsky A (2019). The impact of air exchange rate on ambient air pollution exposure and inequalities across all residential parcels in Massachusetts. J Expo Sci Environ Epidemiol..

[85] Guo H (2020). Who are more exposed to PM2.5 pollution: a mobile phone data approach. Environ Int..

[86] Yu S, Zhu X, He Q (2020). An assessment of urban park access using house-level data in urban China: through the lens of social equity. Int J Environ Res Public Health..

[87] Chillrud SN (2021). The effect of clean cooking interventions on mother and child personal exposure to air pollution: results from the Ghana Randomized Air Pollution and Health Study (GRAPHS). J Expo Sci Environ Epidemiol..

[88] Sanchez M (2020). Personal exposure to particulate matter in peri-urban India: predictors and association with ambient concentration at residence. J Expo Sci Environ Epidemiol..

[89] Benka-Coker ML (2018). Exposure to household air pollution from biomass cookstoves and levels of fractional exhaled nitric oxide (FeNO) among Honduran women. Int J Environ Res Public Health..

[90] Xie M (2020). Household exposure to secondhand smoke among Chinese children: status, determinants, and co-exposures. Int J Environ Res Public Health..

[91] Wang VA (2021). Acculturation and endocrine disrupting chemical-associated personal care product use among US-based foreign-born Chinese women of reproductive age. J Expo Sci Environ Epidemiol..

[92] Collins HN (2021). Differences in personal care product use by race/ethnicity among women in California: implications for chemical exposures. J Expo Sci Environ Epidemiol..

[93] Mitro SD (2019). Phthalate metabolite exposures among immigrants living in the United States: findings from NHANES, 1999–2014. J Expo Sci Environ Epidemiol..

[94] Wattigney WA (2022). Biomonitoring of per- and polyfluoroalkyl substances in minority angler communities in central New York State. Environ Res..

[95] Egan KB (2021). Blood Lead Levels in U.S. Children Ages 1-11 Years, 1976-2016. Environ Health Perspect..

[96] Jukic AMZ (2021). A prospective study of maternal 25-hydroxyvitamin D (25OHD) in the first trimester of pregnancy and second trimester heavy metal levels. Environ Res..

[97] Berendes DM (2020). Variation in E. coli concentrations in open drains across neighborhoods in Accra, Ghana: The influence of onsite sanitation coverage and interconnectedness of urban environments. Int J Hyg Environ Health..

[98] Elf JL (2018). Sources of household air pollution and their association with fine particulate matter in low-income urban homes in India. J Expo Sci Environ Epidemiol..

[99] Shrestha PM (2019). Impact of outdoor air pollution on indoor air quality in low-income homes during wildfire seasons. Int J Environ Res Public Health..

[100] Chu MT (2021). Real-time indoor PM(2.5) monitoring in an urban cohort: Implications for exposure disparities and source control. Environ Res..

[101] Nadybal SM, Collins TW, Grineski SE (2020). Light pollution inequities in the continental United States: A distributive environmental justice analysis. Environ. Res..

[102] Fong KC, Mehta NK, Bell ML (2020). Disparities in exposure to surrounding greenness related to proportion of the population that were immigrants to the United States. Int J Hyg Environ Health..

[103] Mitchell BC, Chakraborty J, Basu P (2021). Social inequities in urban heat and greenspace: analyzing climate justice in Delhi, India. Int J Environ Res Public Health..

[104] Mikati I (2018). Disparities in distribution of particulate matter emission sources by race and poverty status. Am J Public Health..

[105] Khabo-Mmekoa CMN, Momba MNB (2019). The impact of social disparities on microbiological quality of drinking water supply in Ugu District Municipality of Kwazulu-Natal Province, South Africa. Int J Environ Res Public Health..

[106] Fawkes L, Sansom G (2021). Preliminary study of lead-contaminated drinking water in public parks-an assessment of equity and exposure risks in two Texas communities. Int J Environ Res Public Health..

[107] Voelkel J (2018). Assessing vulnerability to urban heat: a study of disproportionate heat exposure and access to refuge by socio-demographic status in Portland, Oregon. Int J Environ Res Public Health..

[108] Liu H, Bai X, Pang X (2020). Intercity variability and local factors influencing the level of pesticide residues in marketed fruits and vegetables of China. Sci Total Environ..

[109] Kong YL (2020). Socio-economic factors related to drinking water source and sanitation in Malaysia. Int J Environ Res Public Health..

[110] Luo Q (2018). A spatio-temporal pattern and socio-economic factors analysis of improved sanitation in China, 2006(-)2015. Int J Environ Res Public Health..

[111] Ramphal B (2022). Noise complaint patterns in New York City from January 2010 through February 2021: Socioeconomic disparities and COVID-19 exacerbations. Environ. Res..

[112] Yang Y (2019). Spatio(-)temporal relationship and evolvement of socioeconomic factors and PM(2.5) in China during 1998(-)2016. Int J Environ Res Public Health..

[113] Xiao Q (2020). Changes in spatial patterns of PM(2.5) pollution in China 2000-2018: Impact of clean air policies. Environ Int..

[114] Elford S, Adams MD (2021). Associations between socioeconomic status and ultrafine particulate exposure in the school commute: An environmental inequality study for Toronto, Canada. Environ Res..

[115] Yan D (2022). Exploring the real contribution of socioeconomic variation to urban PM(2.5) pollution: New evidence from spatial heteroscedasticity. Sci Total Environ..

[116] Park C (2019). Urinary phthalate metabolite and bisphenol A levels in the Korean adult population in association with sociodemographic and behavioral characteristics: Korean National Environmental Health Survey (KoNEHS) 2012-2014. Int J Hyg Environ Health..

[117] Wesselink AK (2021). Correlates of urinary concentrations of phthalate and phthalate alternative metabolites among reproductive-aged Black women from Detroit, Michigan. J Expo Sci Environ Epidemiol..

[118] Dialesandro J (2021). Dimensions of thermal inequity: neighborhood social demographics and urban heat in the southwestern U.S. Int J Environ Res Public Health..

[119] Wang Q (2021). The relationship between population heat vulnerability and urbanization levels: A county-level modeling study across China. Environ Int..

[120] Abbasi A, Pals B, Gazze L (2020). Policy changes and child blood lead levels by age 2 years for children born in Illinois, 2001-2014. Am J Public Health..

[121] Liu X (2021). Novel application of machine learning algorithms and model-agnostic methods to identify factors influencing childhood blood lead levels. Environ Sci Technol..

[122] Browning M, Rigolon A (2018). Do income, race and ethnicity, and sprawl influence the greenspace-human health link in city-level analyses? Findings from 496 cities in the United States. Int J Environ Res Public Health..

[123] Carrion D (2019). Examining the relationship between household air pollution and infant microbial nasal carriage in a Ghanaian cohort. Environ Int..

[124] Flanagan E (2019). Connecting air pollution exposure to socioeconomic status: a cross-sectional study on environmental injustice among pregnant women in Scania, Sweden. Int J Environ Res Public Health..

[125] Aune KT, Gesch D, Smith GS (2020). A spatial analysis of climate gentrification in Orleans Parish, Louisiana post-Hurricane Katrina. Environ Res..

[126] Masri S (2021). Disproportionate impacts of wildfires among elderly and low-income communities in California from 2000-2020. Int J Environ Res Public Health..

[127] Chakraborty J, Collins TW, Grineski SE (2019). Exploring the environmental justice implications of Hurricane Harvey flooding in Greater Houston, Texas. Am J Public Health..

[128] Lieberman-Cribbin W (2021). Socioeconomic disparities in incidents at toxic sites during Hurricane Harvey. J Expo Sci Environ Epidemiol..

[129] Chakraborty J, Basu P (2018). Linking industrial hazards and social inequalities: environmental injustice in Gujarat, India. Int J Environ Res Public Health..

[130] Wortzel JD (2021). Ascertainment bias in a historic cohort study of residents in an asbestos manufacturing community. Int J Environ Res Public Health..

[131] Baek M (2021). Neighborhood-level lead paint hazard for children under 6: a tool for proactive and equitable intervention. Int J Environ Res Public Health..

[132] Dietrich M (2019). The first pollution investigation of road sediment in Gary, Indiana: Anthropogenic metals and possible health implications for a socioeconomically disadvantaged area. Environ Int..

[133] Wheeler DC (2019). Explaining variation in elevated blood lead levels among children in Minnesota using neighborhood socioeconomic variables. Sci Total Environ..

[134] Berman T (2018). Socioeconomic inequalities in exposure to environmental tobacco smoke in children in Israel. Environ Int..

[135] Xiao C (2020). Housing conditions, neighborhood physical environment, and secondhand smoke exposure at home: evidence from Chinese rural-to-urban migrant workers. Int J Environ Res Public Health..

[136] Alves J (2020). Change in the prevalence and social patterning of first-and second-hand smoking in Portugal: a repeated cross-sectional study (2005 and 2014). Int J Environ Res Public Health..

[137] Min E (2021). Quantifying the distribution of environmental health threats and hazards in Washington State using a cumulative environmental inequality index. Environ Justice.

[138] Bauza V (2020). Enteric pathogens from water, hands, surface, soil, drainage ditch, and stream exposure points in a low-income neighborhood of Nairobi, Kenya. Sci Total Environ..

[139] Daniel D (2019). Understanding the effect of socio-economic characteristics and psychosocial factors on household water treatment practices in rural Nepal using Bayesian Belief Networks. Int J Hyg Environ Health..

[140] Su B, Wu L (2020). Occupants' health and their living conditions of remote Indigenous communities in New Zealand. Int J Environ Res Public Health..

[141] Groot J (2022). Indoor home environments of Danish children and the socioeconomic position and health of their parents: a descriptive study. Environ. Int..

[142] Coombs K (2018). Variability of indoor fungal microbiome of green and non-green low-income homes in Cincinnati, Ohio. Sci Total Environ..

[143] Hicks DJ (2020). Census demographics and Chlorpyrifos use in California's Central Valley, 2011-15: a distributional environmental justice analysis. Int J Environ Res Public Health..

[144] Masri S (2020). Social and spatial distribution of soil lead concentrations in the City of Santa Ana, California: Implications for health inequities. Sci Total Environ..

[145] O'Shea MJ (2021). Lead pollution, demographics, and environmental health risks: the case of Philadelphia, USA. Int J Environ Res Public Health..

[146] Rehling J (2021). Socioeconomic differences in walking time of children and adolescents to public green spaces in urban areas-results of the German Environmental Survey (2014-2017). Int J Environ Res Public Health..

[147] Lieberman-Cribbin W (2021). Unequal social vulnerability to Hurricane Sandy flood exposure. J Expo Sci Environ Epidemiol..

[148] Li B, Xiao D (2021). The impact of income inequality on subjective environmental pollution: individual evidence from China. Int J Environ Res Public Health..

[149] Quandt SA (2020). Using life history calendars to estimate in utero and early life pesticide exposure of Latinx children in farmworker families. Int J Environ Res Public Health..

[150] Konkle SL (2020). National secular trends in ambient air volatile organic compound levels and biomarkers of exposure in the United States. Environ. Res..

[151] Hoshiko S (2019). Differences in prenatal tobacco exposure patterns among 13 race/ethnic groups in California. Int J Environ Res Public Health..

[152] Persson A (2019). Is moving to a greener or less green area followed by changes in physical activity?. Health Place..

[153] Buck Louis GM (2018). Endocrine disruptors and neonatal anthropometry, NICHD Fetal Growth Studies - Singletons. Environ Int..

[154] Symanski E (2020). Metal air pollution partnership solutions: building an academic-government-community-industry collaboration to improve air *quality and health in environmental justice communities in Houston*. Environ Health..

[155] Sterrett ME (2021). Maternal food and beverage consumption behaviors and discrepant phthalate exposure by race. Int J Environ Res Public Health..

[156] Buckley JP (2019). Ultra-processed food consumption and exposure to phthalates and bisphenols in the US National Health and Nutrition Examination Survey, 2013-2014. Environ. Int..

[157] Ding N (2020). Longitudinal trends in perfluoroalkyl and polyfluoroalkyl substances among multiethnic midlife women from 1999 to 2011: the Study of Women's Health Across the Nation. Environ Int..

[158] Lin PD (2021). Temporal trends of concentrations of per- and polyfluoroalkyl substances among adults with overweight and obesity in the United States: results from the Diabetes Prevention Program and NHANES. Environ Int..

[159] Williamson DHZ (2020). A scoping review of capacity-building efforts to address environmental justice concerns. Int J Environ Res Public Health..

[160] Dodson RE (2021). Personal care product use among diverse women in California: Taking Stock Study. J Expo Sci Environ Epidemiol..

[161] Nolan JES (2021). "Freedom to Breathe": Youth Participatory Action Research (YPAR) to Investigate Air Pollution Inequities in Richmond, CA. Int J Environ Res Public Health..

[162] Barton KE (2020). Sociodemographic and behavioral determinants of serum concentrations of per- and polyfluoroalkyl substances in a community highly exposed to aqueous film-forming foam contaminants in drinking water. Int J Hyg Environ Health..

[163] Kulinkina AV (2020). Rural Ghanaian households are more likely to use alternative unimproved water sources when water from boreholes has undesirable organoleptic characteristics. Int J Hyg Environ Health..

[164] Christidis T (2019). Low concentrations of fine particle air pollution and mortality in the Canadian Community Health Survey cohort. Environ. Health..

[165] Dehom S (2021). Racial difference in the association of long-term exposure to fine particulate matter (PM(2.5)) and cardiovascular disease mortality among renal transplant recipients. Int J Environ Res Public Health..

[166] Fong KC, Bell ML (2021). Do fine particulate air pollution (PM(2.5)) exposure and its attributable premature mortality differ for immigrants compared to those born in the United States?. Environ Res..

[167] Han C (2021). Socioeconomic disparity in the association between long-term exposure to PM(2.5) and mortality in 2640 Chinese counties. Environ Int..

[168] Jorgenson AK (2021). Inequality amplifies the negative association between life expectancy and air pollution: A cross-national longitudinal study. Sci Total Environ..

[169] Kazemiparkouhi F (2022). The impact of Long-Term PM(2.5) constituents and their sources on specific causes of death in a US Medicare cohort. Environ Int..

[170] Lee H (2018). Ambient air pollution and completed suicide in 26 South Korean cities: Effect modification by demographic and socioeconomic factors. Sci Total Environ..

[171] Loizeau M (2018). Does the air pollution model influence the evidence of socio-economic disparities in exposure and susceptibility?. Environ Res..

[172] Pope CA (2019). Mortality risk and fine particulate air pollution in a large, representative cohort of U.S. adults. Environ Health Perspect..

[173] Saez M, Lopez-Casasnovas G (2019). Assessing the effects on health inequalities of differential exposure and differential susceptibility of air pollution and environmental noise in Barcelona, 2007-2014. Int J Environ Res Public Health..

[174] Sohrabi S, Zietsman J, Khreis H (2020). Burden of disease assessment of ambient air pollution and premature mortality in urban areas: the role of socioeconomic status and transportation. Int J Environ Res Public Health..

[175] Son JY (2020). Health disparities attributable to air pollutant exposure in North Carolina: Influence of residential environmental and social factors. Health Place..

[176] Son JY (2021). Long-term exposure to PM2.5 and mortality for the older population: effect modification by residential greenness. Epidemiology..

[177] Thind MPS (2019). Fine particulate air pollution from electricity generation in the US: health impacts by race, income, and geography. Environ Sci Technol..

[178] Berger K (2018). Association of prenatal urinary concentrations of phthalates and bisphenol A and pubertal timing in boys and girls. Environ. Health Perspect..

[179] Binder AM (2018). Childhood and adolescent phenol and phthalate exposure and the age of menarche in Latina girls. Environ Health..

[180] Daniel S (2020). Prenatal and early childhood exposure to phthalates and childhood behavior at age 7 years. Environ Int..

[181] Daniel S (2020). Perinatal phthalates exposure decreases fine-motor functions in 11-year-old girls: Results from weighted Quantile sum regression. Environ Int..

[182] Loftus CT (2021). Exposure to prenatal phthalate mixtures and neurodevelopment in the Conditions Affecting Neurocognitive Development and Learning in Early childhood (CANDLE) study. Environ Int..

[183] McDonald JA (2018). Hair product use, age at menarche and mammographic breast density in multiethnic urban women. Environ Health..

[184] Perera FP (2018). Combined effects of prenatal exposure to polycyclic aromatic hydrocarbons and material hardship on child ADHD behavior problems. Environ Res..

[185] Hu K (2019). Evidence for urban-rural disparity in temperature-mortality relationships in Zhejiang Province, China. Environ. Health Perspect..

[186] Li Y (2019). Heatwave events and mortality outcomes in Memphis, Tennessee: testing effect modification by socioeconomic status and urbanicity. Int J Environ Res Public Health..

[187] Murage P, Hajat S, Bone A (2018). Variation in cold-related mortality in england since the introduction of the cold weather plan: which areas have the greatest unmet needs?. Int J Environ Res Public Health..

[188] Xing Q (2020). Impacts of urbanization on the temperature-cardiovascular mortality relationship in Beijing, China. Environ Res..

[189] Yang J (2019). Heatwave and mortality in 31 major Chinese cities: definition, vulnerability and implications. Sci Total Environ..

[190] Basu R (2018). Temperature and term low birth weight in California. Am J Epidemiol..

[191] Li C (2021). Temperature variation and preterm birth among live singleton deliveries in Shenzhen, China: A time-to-event analysis. Environ Res..

[192] Smith ML, Hardeman RR (2020). Association of summer heat waves and the probability of preterm birth in Minnesota: an exploration of the intersection of race and education. Int J Environ Res Public Health..

[193] Son JY (2019). Impacts of high temperature on adverse birth outcomes in Seoul, Korea: Disparities by individual- and community-level characteristics. Environ Res..

[194] Berman JD (2019). Acute air pollution exposure and the risk of violent behavior in the United States. Epidemiology..

[195] Buthelezi SA (2019). Household fuel use for heating and cooking and respiratory health in a low-income, South African coastal community. Int J Environ Res Public Health..

[196] Kulick ER (2020). Long-term exposure to ambient air pollution, APOE-epsilon4 status, and cognitive decline in a cohort of older adults in northern Manhattan. Environ Int..

[197] Daouda M (2021). Association between county-level coal-fired power plant pollution and racial disparities in preterm births from 2000 to 2018. Environ Res Lett..

[198] Enders C (2019). Exposure to coarse particulate matter during gestation and term low birthweight in California: variation in exposure and risk across region and socioeconomic subgroup. Sci Total Environ..

[199] Roberman J, Emeto TI, Adegboye OA (2021). Adverse birth outcomes due to exposure to household air pollution from unclean cooking fuel among women of reproductive age in Nigeria. Int J Environ Res Public Health..

[200] Chevrier J (2019). Associations of maternal exposure to dichlorodiphenyltrichloroethane and pyrethroids with birth outcomes among participants in the Venda Health Examination of Mothers, Babies and Their Environment residing in an area sprayed for malaria control. Am J Epidemiol..

[201] Gaston SA (2020). Hair maintenance and chemical hair product usage as barriers to physical activity in childhood and adulthood among African American women. Int J Environ Res Public Health..

[202] Heggeseth BC (2019). Heterogeneity in childhood body mass trajectories in relation to prenatal phthalate exposure. Environ Res..

[203] Basu R (2018). Examining the association between apparent temperature and mental health-related emergency room visits in California. Am J Epidemiol..

[204] Pearson D (2020). Temperature and hand, foot and mouth disease in California: An exploratory analysis of emergency department visits by season, 2005-2013. Environ Res..

[205] Knapp M (2018). The relationships between park quality, park usage, and levels of physical activity in low-income, African American neighborhoods. Int J Environ Res Public Health..

[206] Montresor-Lopez JA (2021). The relationship between traffic-related air pollution exposures and allostatic load score among youth with type 1 diabetes in the SEARCH cohort. Environ. Res..

[207] Zhang L, Wu L (2021). Effects of environmental quality perception on depression: subjective social class as a mediator. Int J Environ Res Public Health..

[208] Zhang M (2021). In utero exposure to heavy metals and trace elements and childhood blood pressure in a U.S. urban, low-income, minority birth cohort. Environ. Health Perspect..

[209] Zandieh R, Martinez J, Flacke J (2019). Older adults' outdoor walking and inequalities in neighbourhood green spaces characteristics. Int J Environ Res Public Health..

[210] Bi C (2018). Phthalates and organophosphates in settled dust and HVAC filter dust of U.S. low-income homes: Association with season, building characteristics, and childhood asthma. Environ Int..

[211] Rana J (2019). Associations between indoor air pollution and acute respiratory infections among under-five children in Afghanistan: do SES and sex matter?. Int J Environ Res Public Health..

[212] Tamire M (2019). Respiratory symptoms and lung function among Ethiopian women in relation to household fuel use. Int J Environ Res Public Health..

[213] Vowles M (2020). Investigation of the environmental and socio-economic characteristics of counties with a high asthma burden to focus asthma action in Utah. Int J Environ Res Public Health..

[214] Workman B (2021). Evaluation of a program to reduce home environment risks for children with asthma residing in urban areas. Int J Environ Res Public Health..

[215] Bravo MA, Miranda ML (2021). Effects of accumulated environmental, social and host exposures on early childhood educational outcomes. Environ. Res..

[216] Frediani JK (2018). Arsenic exposure and risk of nonalcoholic fatty liver disease (NAFLD) among U.S. adolescents and adults: an association modified by race/ethnicity, NHANES 2005-2014. Environ Health.

[217] Milando CW (2021). Modeling the impact of exposure reductions using multi-stressor epidemiology, exposure models, and synthetic microdata: an application to birthweight in two environmental justice communities. J Expo Sci Environ Epidemiol..

[218] Pun VC, Manjourides J, Suh HH (2018). Association of neighborhood greenness with self-perceived stress, depression and anxiety symptoms in older U.S adults. Environ. Health.

[219] Sim K (2020). Nonlinear temperature-suicide association in Japan from 1972 to 2015: Its heterogeneity and the role of climate, demographic, and socioeconomic factors. Environ. Int..

[220] Hyland C (2021). Associations between pesticide mixtures applied near home during pregnancy and early childhood with adolescent behavioral and emotional problems in the CHAMACOS study. Environ Epidemiol.

[221] Bangma J (2020). Identifying risk factors for levels of per- and polyfluoroalkyl substances (PFAS) in the placenta in a high-risk pregnancy cohort in North Carolina. Environ Sci Technol..

[222] Papatheodorou S (2021). Residential radon exposure and hypertensive disorders of pregnancy in Massachusetts, USA: A cohort study. Environ Int..

[223] Varnell RR (2021). Menstrual cycle patterns and irregularities in hired Latinx child farmworkers. J. Occup. Environ. Med..

[224] Humphrey JL (2019). Social and environmental neighborhood typologies and lung function in a low-income, urban population. Int J Environ Res Public Health..

[225] Bowe B (2021). Ambient fine particulate matter air pollution and the risk of hospitalization among COVID-19 positive individuals: cohort study. Environ Int..

[226] Klompmaker JO (2021). County-level exposures to greenness and associations with COVID-19 incidence and mortality in the United States. Environ Res..

[227] Bravo MA, Anthopolos R, Miranda ML (2019). Characteristics of the built environment and spatial patterning of type 2 diabetes in the urban core of Durham, North Carolina. J Epidemiol Community Health..

[228] Allen EM, Munala L, Henderson JR (2021). Kenyan women bearing the cost of climate change. Int J Environ Res Public Health..

[229] Sun S (2020). Tropical cyclones and risk of preterm birth: a retrospective analysis of 20 million births across 378 US counties. Environ Int..

[230] Casey JA (2018). Retirements of coal and oil power plants in California: association with reduced preterm birth among populations nearby. Am J Epidemiol..

[231] Leuenberger A (2021). "It is like we are living in a different world": health inequity in communities surrounding industrial mining sites in Burkina Faso, Mozambique, and Tanzania. Int J Environ Res Public Health..

[232] Williams AD (2019). Ambient volatile organic compounds and racial/ethnic disparities in gestational diabetes mellitus: are Asian/Pacific Islander women at greater risk?. Am. J. Epidemiol..

[233] Knobel P (2021). Associations of objective and perceived greenness measures with cardiovascular risk factors in Philadelphia, PA: a spatial analysis. Environ Res..

[234] Korn A (2018). Physical and mental health impacts of household gardens in an urban slum in Lima, Peru. Int J Environ Res Public Health..

[235] Hobbs M (2020). Area-level deprivation, childhood dental ambulatory sensitive hospitalizations and community water fluoridation: evidence from New Zealand. Int. J. Epidemiol..

[236] Winter SC, Obara LM, Barchi F (2019). Environmental correlates of health-related quality of life among women living in informal settlements in Kenya. Int J Environ Res Public Health..

[237] Ekenga CC, Yeung CY, Oka M (2019). Cancer risk from air toxics in relation to neighborhood isolation and sociodemographic characteristics: a spatial analysis of the St. Louis metropolitan area, USA. Environ Res..

[238] Guo H (2019). Air pollution and lung cancer incidence in China: who are faced with a greater effect?. Environ Int..

[239] Li H (2021). Associations of long-term exposure to environmental noise and outdoor light at night with age at natural menopause in a US women cohort. Environ Epidemiol..

[240] De Roos AJ (2020). Ambient daily pollen levels in association with asthma exacerbation among children in Philadelphia, Pennsylvania. Environ Int..

[241] Eick SM (2019). Socioeconomic status and the association between arsenic exposure and type 2 diabetes. Environ Res..

[242] Bloom MS (2022). Association between gestational PFAS exposure and Children's adiposity in a diverse population. Environ Res..

[243] Kaiser ML, Hand MD, Pence EK (2020). Individual and community engagement in response to environmental challenges experienced in four low-income urban neighborhoods. Int J Environ Res Public Health..

[244] Sampson NR (2018). "We're just sitting ducks": recurrent household flooding as an underreported environmental health threat in Detroit's changing climate. Int J Environ Res Public Health..

[245] Crouse DL (2018). Associations between living near water and risk of mortality among urban Canadians. Environ Health Perspect..

[246] Bhatta DN, Glantz S (2019). Parental tobacco use and child death: analysis of data from demographic and health surveys from South and South East Asian countries. Int J Epidemiol..

[247] Wasserman GA (2018). A cross-sectional study of water arsenic exposure and intellectual function in adolescence in Araihazar, Bangladesh. Environ Int..

[248] Williams AA (2019). Building vulnerability in a changing climate: indoor temperature exposures and health outcomes in older adults living in public housing during an extreme heat event in Cambridge, MA. Int J Environ Res Public Health..

[249] Malig BJ (2019). Associations between ambient temperature and hepatobiliary and renal hospitalizations in California, 1999 to 2009. Environ Res..

[250] Fong KC (2018). Residential greenness and birthweight in the state of Massachusetts, USA. Int J Environ Res Public Health..

[251] Niehoff NM (2021). Metals and breast cancer risk: a prospective study using toenail biomarkers. Am J Epidemiol..

[252] Huang H (2021). Child marriage, maternal serum metal exposure, and risk of preterm birth in rural Bangladesh: evidence from mediation analysis. J Expo Sci Environ Epidemiol..

[253] Liu J (2022). Blood lead levels mediate the relationship between social adversity and child externalizing behavior. Environ Res..

[254] Song L (2020). Ambient air pollution as a mediator in the pathway linking race/ethnicity to blood pressure elevation: The multi-ethnic study of atherosclerosis (MESA). Environ Res..

[255] Corlin L (2018). Relationship of time-activity-adjusted particle number concentration with blood pressure. Int J Environ Res Public Health..

[256] Grazuleviciene R (2020). Neighborhood social and built environment and disparities in the risk of hypertension: a cross-sectional study. Int J Environ Res Public Health..

[257] Rajkumar S (2019). Household air pollution from biomass-burning cookstoves and metabolic syndrome, blood lipid concentrations, and waist circumference in Honduran women: A cross-sectional study. Environ Res..

[258] Smith, D. *In historic move, Los Angeles bans new oil wells, phases out existing ones*. 2022 06 Mar 2023]; Available from: https://www.latimes.com/california/story/2022-12-02/in-historic-move-l-a-bans-new-oil-wells-phases-out-existing-ones.

[259] Lievanos RS (2019). Racialized structural vulnerability: neighborhood racial composition, concentrated disadvantage, and fine particulate matter in California. Int J Environ Res Public Health..

[260] Johnson Gaither C (2019). African American exposure to prescribed fire smoke in Georgia, USA. Int J Environ Res Public Health..

[261] Yang Y, Lan H, Li J (2019). Spatial econometric analysis of the impact of socioeconomic factors on PM(2.5) concentration in China's inland cities: a case study from Chengdu Plain Economic Zone. Int J Environ Res Public Health..

[262] Wheeler DC, Boyle J, Nelson EJ (2022). Modeling annual elevated blood lead levels among children in Maryland in relation to neighborhood deprivation. Sci Total Environ..

[263] Driver A (2019). Utilization of the Maryland Environmental Justice Screening Tool: a Bladensburg, Maryland case study. Int J Environ Res Public Health..

[264] Padula AM (2021). Drinking water contaminants in California and hypertensive disorders in pregnancy. Environ Epidemiol.

[265] Mousavi A (2021). Impact of 4th of July fireworks on spatiotemporal PM(2.5) concentrations in California based on the PurpleAir Sensor Network: implications for policy and environmental justice. Int J Environ Res Public Health..

[266] Tanzer R (2019). Demonstration of a low-cost multi-pollutant network to quantify intra-urban spatial variations in air pollutant source impacts and to evaluate environmental justice. Int J Environ Res Public Health..

[267] Zhao J, Gladson L, Cromar K (2018). A novel environmental justice indicator for managing local air pollution. Int J Environ Res Public Health..

[268] August, L., et al. *CalEnvironScreen 4.0*. 2021 8 Nov 2022]; Available from: https://oehha.ca.gov/media/downloads/calenviroscreen/report/calenviroscreen40reportf2021.pdf.

[269] Council on Environmental Quality. *Climate and economic justice screening tool*. 2022 6 Feb 2023]; Available from: https://screeningtool.geoplatform.gov/en/#3/33.47/-97.5.

[270] US Environmental Protection Agency. *EJScreen: environmental justice screening and mapping tool*. 06 Feb 2023]; Available from: https://www.epa.gov/ejscreen.

[271] Balakrishnan, C., et al. *Screening for environmental justice: a framework for comparing national, state, and local data tools* 2022 06 Mar 2023]; Available from: https://www.urban.org/sites/default/files/2022-11/Screening%20for%20Environmental%20Justice-%20A%20Framework%20for%20Comparing%20National%2C%20State%2C%20and%20Local%20Data%20Tools.pdf.

[272] Grineski SEWCT, Rubio R (2019). Distributional environmental injustices for a minority group without minority status: Arab Americans and residential exposure to carcinogenic air pollution in the US. Int J Environ Res Public Health..

[273] Reames TG, Bravo MA (2019). People, place and pollution: Investigating relationships between air quality perceptions, health concerns, exposure, and individual- and area-level characteristics. Environ Int..

[274] Zayas-Costa M (2021). Mental health outcomes in Barcelona: the interplay between gentrification and greenspace. Int J Environ Res Public Health..

[275] Cushing L (2015). The haves, the have-nots, and the health of everyone: the relationship between social inequality and environmental quality. Annu. Rev. Public Health.

[276] Smith GS (2020). Impacts of gentrification on health in the US: a systematic review of the literature. J. Urban Health.

[277] Bhavsar NA, Kumar M, Richman L (2020). Defining gentrification for epidemiologic research: a systematic review. PLoS One.

[278] Oka M, Wong DW (2014). Capturing the two dimensions of residential segregation at the neighborhood level for health research. Front Public Health..

[279] Anguelovski I (2019). Why green “climate gentrification” threatens poor and vulnerable populations. Proc Natl Acad Sci..

[280] Maantay JA, Maroko AR (2018). Brownfields to Greenfields: Environmental Justice Versus Environmental Gentrification. Int J Environ Res Public Health..

[281] Anselin, L. *Local Spatial Autocorrelation*. 2020 09 Mar 2023]; Available from: https://geodacenter.github.io/workbook/6a_local_auto/lab6a.html#adjusting-p-values.

[282] Payne-Sturges DC, Gee GC, Cory-Slechta DA (2021). Confronting racism in environmental health sciences: moving the science forward for eliminating racial inequities. Environ Health Perspect..

[283] Boyd RW (2020). On racism: a new standard for publishing on racial health inequities.

[284] Buehler JW (1999). Abandoning race as a variable in public health research. Am J Public Health..

[285] Williams DR, Lavizzo-Mourey R, Warren RC (1994). The concept of race and health status in America. Public Health Rep..

[286] Ford CL, Airhihenbuwa CO (2010). Critical race theory, race equity, and public health: toward antiracism praxis. Am J Public Health..

[287] Chadha, N., et al. *Toward the abolition of biological race in medicine*. 2021 27 Jan 2023]; Available from: https://belonging.berkeley.edu/toward-abolition-biological-race-medicine-8.

[288] Chowkwanyun M, Reed AL (2020). Racial health disparities and Covid-19 - caution and context. N Engl J Med..

[289] Benmarhnia T, Hajat A, Kaufman JS (2021). Inferential challenges when assessing racial/ethnic health disparities in environmental research. Environ Health..

[290] National Academies of Sciences, E., and Medicine (2022). Measuring sex, gender identity, and sexual orientation.

[291] Goldsmith L, Bell ML (2022). Queering environmental justice: unequal environmental health burden on the LGBTQ+ community. Am J Public Health..

[292] Borunda, A. *The origins of environmental justice—and why it’s finally getting the attention it deserves*. 2021 31 Jan 2023]; Available from: https://www.nationalgeographic.com/environment/article/environmental-justice-origins-why-finally-getting-the-attention-it-deserves

[293] Winters, J. *LA bans oil and gas drilling in historic vote*. 2022 31 Jan 2023]; Available from: https://grist.org/beacon/la-bans-oil-and-gas-drilling-in-historic-vote/.

[294] McLeroy KR (2003). Community-based interventions. Am J Public Health..

[295] Minkler M, Wallerstein N (2011). Community-based participatory research for health: From process to outcomes.

[296] O'Fallon LR, Dearry A (2002). Community-based participatory research as a tool to advance environmental health sciences. Environ Health Perspect..

[297] Lett E (2022). Equity tourism: ravaging the justice landscape. Health Policy (New York)..

[298] Bowleg L (2021). "The master's tools will never dismantle the master's house": ten critical lessons for black and other health equity researchers of color. Health Educ Behav..

[299] Zota AR, Shamasunder B (2021). Environmental health equity: moving toward a solution-oriented research agenda. J Expo Sci Environ Epidemiol..

[300] Welch BM (2022). Associations between prenatal urinary biomarkers of phthalate exposure and preterm birth: a pooled study of 16 us cohorts. JAMA Pediatr..

[301] Swope CB, Hernandez D, Cushing LJ (2022). The relationship of historical redlining with present-day neighborhood environmental and health outcomes: a scoping review and conceptual model. J. Urban Health.

[302] Brown P (2003). Qualitative methods in environmental health research. Environ Health Perspect..

[303] Scammell MK (2010). Qualitative environmental health research: an analysis of the literature, 1991-2008. Environ Health Perspect..

[304] Rosenthal J (2017). Implementation science to accelerate clean cooking for public health. Environ Health Perspect..

[305] Nigra AE, Navas-Acien A (2020). Arsenic in US correctional facility drinking water, 2006-2011. Environ Res..

[306] Carrion D, Belcourt A, Fuller CH (2022). Heading upstream: strategies to shift environmental justice research from disparities to equity. Am J Public Health..

[307] Shelton RC (2021). Application of an antiracism lens in the field of implementation science (IS): recommendations for reframing implementation research with a focus on justice and racial equity. Implementation Res Practice..

[308] Mitchell G, Norman P, Mullin K (2015). Who benefits from environmental policy? An environmental justice analysis of air quality change in Britain, 2001–2011. Environ Res Lett..

[309] Basu P, Chakraborty J (2016). Environmental justice implications of industrial hazardous waste generation in India: a national scale analysis. Environ Res Lett..

[310] Natarajan U, Gonzalez CG, Atapattu SA (2021). Environmental Justice in the Global South, in The Cambridge Handbook of Environmental Justice and Sustainable Development, S.S.

[311] Pellow DN (2004). The politics of illegal dumping: an environmental justice framework. Qual Sociol..

[312] Boone CG, Buckley GL (2017). Historical approaches to environmental justice. The Routledge Handbook of Environmental Justice.

[313] Basu P (2017). Environmental justice in South and Southeast Asia: Inequalities and struggles in rural and urban contexts. The Routledge handbook of environmental justice.

