# Supplementary material for: Methods in Public Health Environmental Justice Research: a Scoping Review from 2018 to 2021
Source: Curr Environ Health Rep. 2023 Aug 15;10(3):312–36. doi: 10.1007/s40572-023-00406-7 (PMC10504232; doi:10.1007/s40572-023-00406-7)
Supplement: Supplementary file 1 — ESM 1 [file 40572_2023_406_MOESM1_ESM.docx]

**Supplementary Information for “Methods in public health environmental justice research: A scoping review from 2018–2021”**

**Figure S1: Health disparity population categories by which articles qualified as environmental justice studies for inclusion in the review.** SES, socioeconomic status


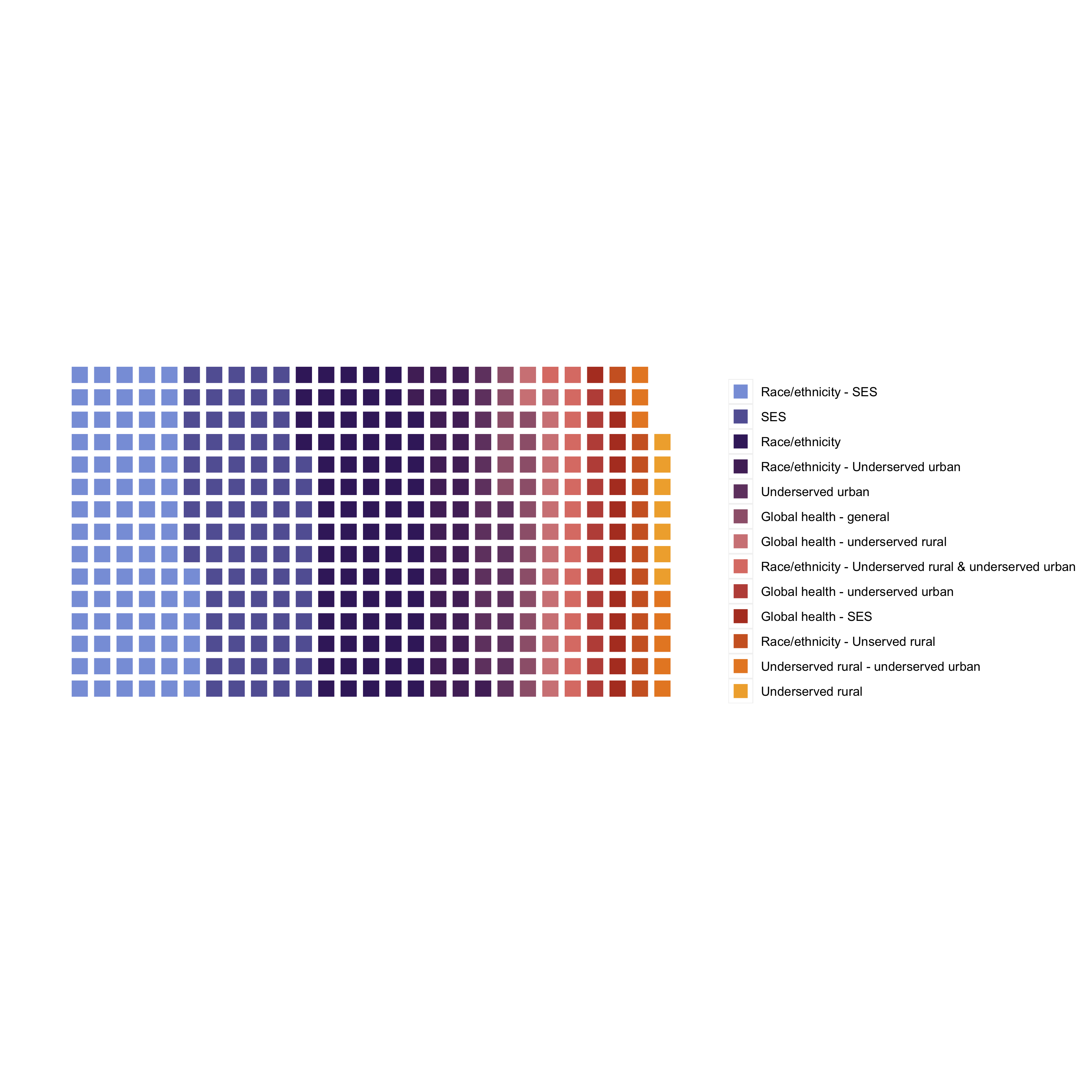


**Figure S2:** **Health disparity population categories by which articles qualified as environmental justice studies for inclusion in the review by study category (epidemiology vs. exposure-only).** SES, socioeconomic status.


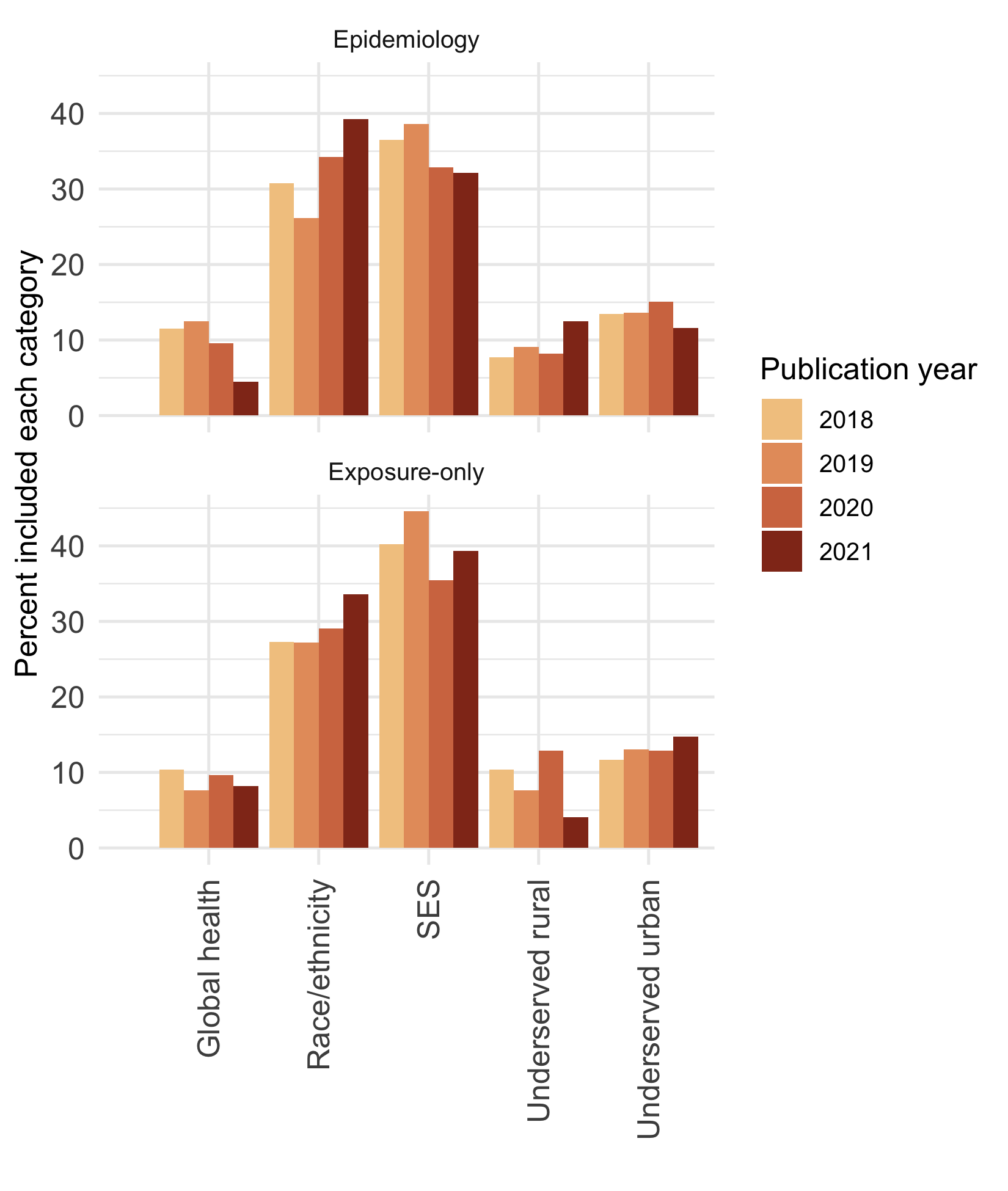


**Figure S3:** **Concordance of corresponding author affiliation and study location country.** Among international studies (left bar), 37% of corresponding authors did not have an affiliation location in the country where the study was conducted. Among US-based studies (right bar), nearly all (98%) corresponding authors had an affiliation with an institution located in the US.

**
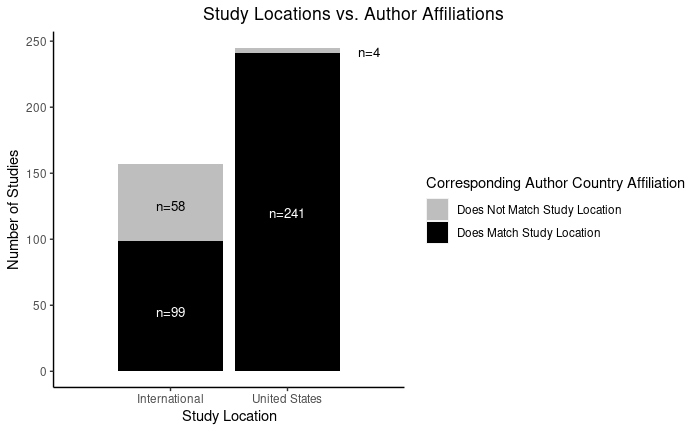
**

**Figure S4: Locations of (A) US-based studies and (B) corresponding author affiliation, 2018-2021.** 50 studies were conducted nationwide (often just continental US) or were nationally representative (dark green nationwide count).

**
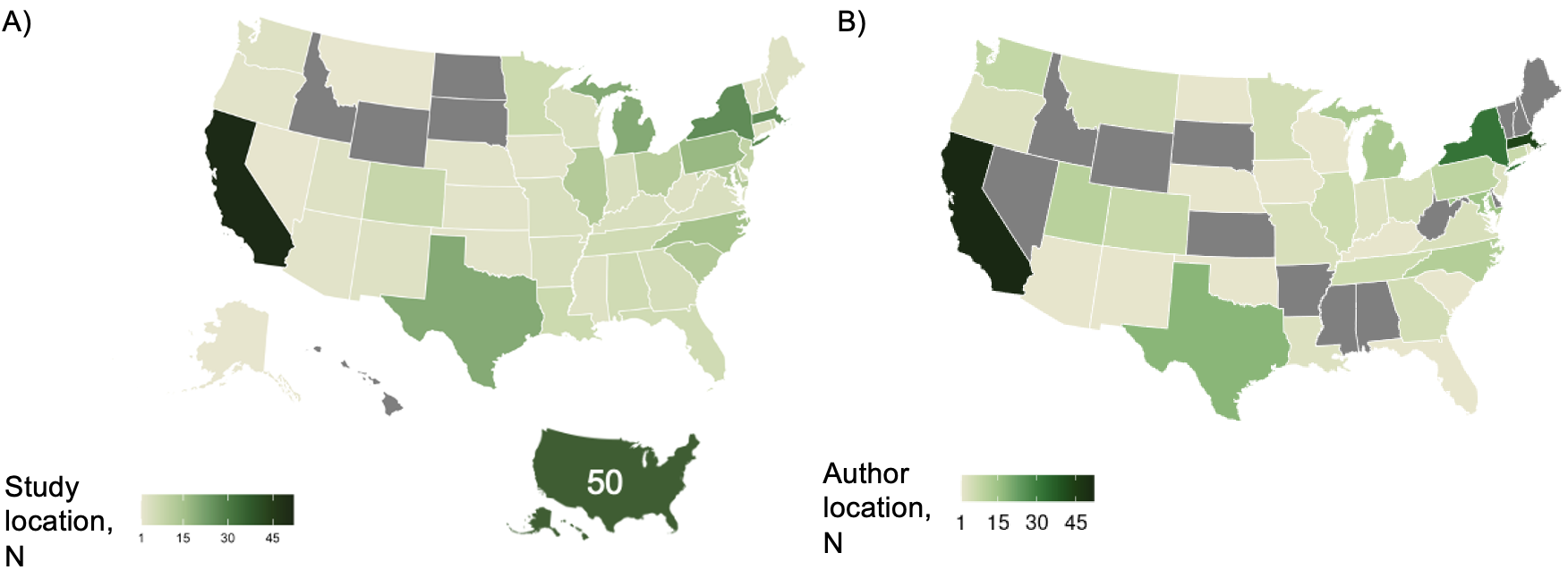
**

**Figure S5:** **Summary of methods used for the most common area-level exposure-only study (air pollution) and the most common personal exposure-only study (chemicals).** Note that no area-level air pollution studies used mediation analyses and no personal-level chemical studies used qualitative methods.


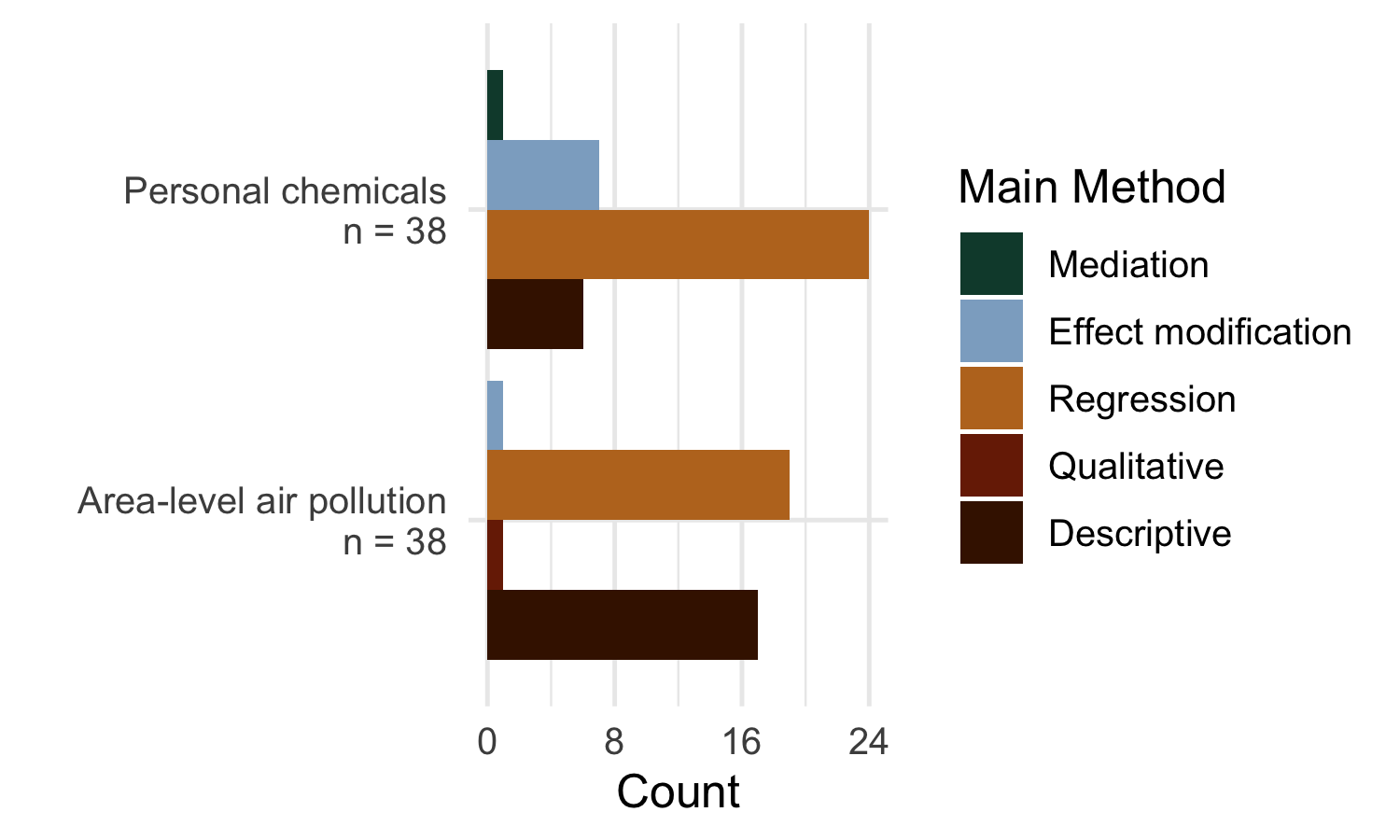


| **Table S1:** **Covidence^a^ data extraction form used to collect information from included articles.** | | |
| --- | --- | --- |
| **Item** | **Prompt** | **Reviewer entry** |
| **SX.1** | **First author (year)** | Text entry field |
| **SX.2** | **Journal** | Text entry field |
| **SX.3** | **Study population** | Text entry field |
| **SX.4** | **Study years** | Text entry field |
| **SX.5** | **Marginalized group of focus / EJ relevant variable** | Text entry field |
| **SX.6** | **Health disparity population** | - Race/ethnicity - SES - Sexual/gender minority - Underserved rural - Underserved urban - Global health |
| **SX.7** | **Study design** | - Exposure science study - personal exposure - Exposure science study - area-level exposure - Intervention study - Epidemiologic study - Cross-sectional - Longitudinal - Retrospective cohort - Prospective cohort - Case-control - Case-crossover - Other |
| **SX.8** | **EJ framework defined/discussed** | - Explicitly states framework (intro) - Some description framework, not adequate (intro) - No description framework (intro) - Explicitly states framework (methods) - Some description framework, not adequate (methods) - No description framework (methods) - Explicitly states framework (discussion) - Some description framework, not adequate (discussion) - No description framework (discussion) |
| **SX.9** | **Marginalized group / EJ predictor variables** | Text entry field |
| **SX.10** | **Marginalized group / EJ outcome variables** | Text entry field |
| **SX.11** | **Marginalized group / EJ mediator variables** | Text entry field |
| **SX.12** | **Marginalized group / EJ effect modifier variables** | Text entry field |
| **SX.13** | **Environmental factor predictor variables** | Text entry field |
| **SX.14** | **Environmental factor outcome variables** | Text entry field |
| **SX.15** | **Health outcome(s)** | Text entry field |
| **SX.16** | **Statistical method used for EJ** | Text entry field |
| **SX.17** | **Main EJ findings**  Please note if data/results were quantitatively reported | Text entry field |
| **SX.18** | **Subjective study quality** | - Top 10% - Top 25% - Average - Below average |
| **SX.19** | **Notes**  Is this a top environmental justice study? Does this paper have an example of great or poor methods? Please note it here. | Text entry field |
| ^a^Covidence is a web-based collaboration software platform that streamlines the production of systematic and other literature reviews. Covidence systematic review software, Veritas Health Innovation, Melbourne, Australia. Available at [www.covidence.org](http://www.covidence.org)  EJ, Environmental justice  🔾: Indicates single user selection possible  ❑: Indicates multiple user selections possible  **Table S2: Corresponding author affiliation for institutions with 3+ papers included in this review.**   \| **Corresponding author's affiliation** \| **Study Count** \| **Percent of Studies** \| \| --- \| --- \| --- \| \| Harvard University \| 20 \| 5.0 \| \| Columbia University \| 16 \| 4.0 \| \| University of California, Berkeley \| 10 \| 2.5 \| \| University of Michigan \| 10 \| 2.5 \| \| Boston University \| 9 \| 2.2 \| \| University of Utah \| 9 \| 2.2 \| \| University of California, San Francisco \| 8 \| 2.0 \| \| University of Maryland \| 8 \| 2.0 \| \| University of Washington \| 8 \| 2.0 \| \| Johns Hopkins University \| 7 \| 1.7 \| \| Yale University \| 7 \| 1.7 \| \| Universitat Pompeu Fabra \| 6 \| 1.5 \| \| University of Texas at El Paso \| 6 \| 1.5 \| \| Barcelona Institute for Global Health \| 5 \| 1.2 \| \| California Environmental Protection Agency \| 5 \| 1.2 \| \| CIBER Epidemiología y Salud Pública \| 5 \| 1.2 \| \| Icahn School of Medicine at Mount Sinai \| 5 \| 1.2 \| \| Sun Yat-Sen University \| 5 \| 1.2 \| \| Tufts University \| 5 \| 1.2 \| \| University of Colorado \| 5 \| 1.2 \| \| US CDC \| 5 \| 1.2 \| \| US NIEHS \| 5 \| 1.2 \| \| Emory University \| 4 \| 1.0 \| \| George Mason University \| 4 \| 1.0 \| \| University of California, Davis \| 4 \| 1.0 \| \| University of Illinois \| 4 \| 1.0 \| \| Zhengzhou University \| 4 \| 1.0 \| \| Beijing Normal University \| 3 \| 0.7 \| \| Brown University \| 3 \| 0.7 \| \| California Department of Public Health \| 3 \| 0.7 \| \| Chinese CDC \| 3 \| 0.7 \| \| Drexel University \| 3 \| 0.7 \| \| McGill University \| 3 \| 0.7 \| \| Monash University \| 3 \| 0.7 \| \| Swiss Tropical and Public Health Institute \| 3 \| 0.7 \| \| Texas A&M \| 3 \| 0.7 \| \| Tulane University \| 3 \| 0.7 \| \| University of Basel \| 3 \| 0.7 \| \| University of California, Irvine \| 3 \| 0.7 \| \| University of California, San Diego \| 3 \| 0.7 \| \| University of Hong Kong \| 3 \| 0.7 \| \| University of Massachusetts \| 3 \| 0.7 \| \| University of North Carolina \| 3 \| 0.7 \| \| University of Pennsylvania \| 3 \| 0.7 \| \| University of Southern California \| 3 \| 0.7 \| \| University of Texas Health Science Center at Houston \| 3 \| 0.7 \| \| US EPA \| 3 \| 0.7 \| \| Wake Forest University \| 3 \| 0.7 \| \| Wuhan University \| 3 \| 0.7 \| | | |

**Table S3:** **Main method used for EJ analyses by exposure-only and epidemiology studies.**

| **Method** | **Exposure-only (n=208)** | **Epidemiology (n=194)** |
| --- | --- | --- |
| Descriptive | 67 (32) | 14 (7) |
| Qualitative | 4 (2) | 5 (3) |
| Main effect regression | 105 (50) | 58 (30) |
| Effect modification | 31 (15) | 112 (58) |
| Mediation | 1 (0.5) | 5 (3) |

**Supplementary Reference List: Full list of the 402 studies included in this review.**

1. Abbasi A, Pals B, Gazze L. Policy Changes and Child Blood Lead Levels by Age 2 Years for Children Born in Illinois, 2001-2014. Am J Public Health. 2020;110(5):734-40.
2. Adeyeye TE, Insaf TZ, Al-Hamdan MZ, Nayak SG, Stuart N, DiRienzo S, et al. Estimating policy-relevant health effects of ambient heat exposures using spatially contiguous reanalysis data. Environ Health. 2019;18(1):35.
3. Aelion CM, Davis HT. Blood lead levels in children in urban and rural areas: Using multilevel modeling to investigate impacts of gender, race, poverty, and the environment. Sci Total Environ. 2019;694:133783.
4. Aerts R, Nemery B, Bauwelinck M, Trabelsi S, Deboosere P, Van Nieuwenhuyse A, et al. Residential green space, air pollution, socioeconomic deprivation and cardiovascular medication sales in Belgium: A nationwide ecological study. Sci Total Environ. 2020;712:136426.
5. Aerts R, Van Overmeire I, Colles A, Andjelkovic M, Malarvannan G, Poma G, et al. Determinants of persistent organic pollutant (POP) concentrations in human breast milk of a cross-sectional sample of primiparous mothers in Belgium. Environ Int. 2019;131:104979.
6. Agay-Shay K, Michael Y, Basagana X, Martinez-Solanas E, Broday D, Lensky IM, et al. Mean and variance of greenness and pregnancy outcomes in Tel Aviv during 2000-14: longitudinal and cross-sectional approaches. Int J Epidemiol. 2019;48(4):1054-72.
7. Ahamad MG, Tanin F, Shrestha N. Household Smoke-Exposure Risks Associated with Cooking Fuels and Cooking Places in Tanzania: A Cross-Sectional Analysis of Demographic and Health Survey Data. Int J Environ Res Public Health. 2021;18(5).
8. Allain ML, Collins TW. Differential Access to Park Space Based on Country of Origin within Miami's Hispanic/Latino Population: A Novel Analysis of Park Equity. Int J Environ Res Public Health. 2021;18(16).
9. Allen EM, Munala L, Henderson JR. Kenyan Women Bearing the Cost of Climate Change. Int J Environ Res Public Health. 2021;18(23).
10. Allotey JA, Boyle M, Sapkota A, Zhu L, Peng RD, Garza MA, et al. Determinants of phthalate exposure among a U.S.-based group of Latino workers. Int J Hyg Environ Health. 2021;234:113739.
11. Alves J, Filipe R, Machado J, Nunes B, Perelman J. Change in the Prevalence and Social Patterning of First-and Second-Hand Smoking in PORTUGAL: A Repeated Cross-Sectional Study (2005 and 2014). Int J Environ Res Public Health. 2020;17(10).
12. Anastasiou E, Feinberg A, Tovar A, Gill E, Ruzmyn Vilcassim MJ, Wyka K, et al. Secondhand smoke exposure in public and private high-rise multiunit housing serving low-income residents in New York City prior to federal smoking ban in public housing, 2018. Sci Total Environ. 2020;704:135322.
13. Aoki Y, Brody DJ. WIC Participation and Blood Lead Levels among Children 1-5 Years: 2007-2014. Environ Health Perspect. 2018;126(6):067011.
14. Araya F, Stingone JA, Claudio L. Inequalities in Exposure to Ambient Air Neurotoxicants and Disparities in Markers of Neurodevelopment in Children by Maternal Nativity Status. Int J Environ Res Public Health. 2021;18(14).
15. Arbuckle TE, Liang CL, Fisher M, Caron NJ, Fraser WD, and the MSG. Exposure to tobacco smoke and validation of smoking status during pregnancy in the MIREC study. J Expo Sci Environ Epidemiol. 2018;28(5):461-9.
16. Arku RE, Ezzati M, Baumgartner J, Fink G, Zhou B, Hystad P, et al. Elevated blood pressure and household solid fuel use in premenopausal women: Analysis of 12 Demographic and Health Surveys (DHS) from 10 countries. Environ Res. 2018;160:499-505.
17. Armijos RX, Weigel MM, Obeng-Gyasi E, Racines-Orbe M. Elevated blood lead and metal/metalloid levels and environmental exposure sources in urban Ecuadorian school-age children and mothers. Int J Hyg Environ Health. 2021;235:113770.
18. Asamoah A, Essumang DK, Muff J, Kucheryavskiy SV, Sogaard EG. Assessment of PCBs and exposure risk to infants in breast milk of primiparae and multiparae mothers in an electronic waste hot spot and non-hot spot areas in Ghana. Sci Total Environ. 2018;612:1473-9.
19. Assefa GM, Sherif S, Sluijs J, Kuijpers M, Chaka T, Solomon A, et al. Gender Equality and Social Inclusion in Relation to Water, Sanitation and Hygiene in the Oromia Region of Ethiopia. Int J Environ Res Public Health. 2021;18(8).
20. Asta F, Michelozzi P, Cesaroni G, De Sario M, Badaloni C, Davoli M, et al. The Modifying Role of Socioeconomic Position and Greenness on the Short-Term Effect of Heat and Air Pollution on Preterm Births in Rome, 2001-2013. Int J Environ Res Public Health. 2019;16(14).
21. Aunan K, Ma Q, Lund MT, Wang S. Population-weighted exposure to PM(2.5) pollution in China: An integrated approach. Environ Int. 2018;120:111-20.
22. Aune KT, Gesch D, Smith GS. A spatial analysis of climate gentrification in Orleans Parish, Louisiana post-Hurricane Katrina. Environ Res. 2020;185:109384.
23. Awuor L, Melles S. The influence of environmental and health indicators on premature mortality: An empirical analysis of the City of Toronto's 140 neighborhoods. Health Place. 2019;58:102155.
24. Baek M, Outrich MB, Barnett KS, Reece J. Neighborhood-Level Lead Paint Hazard for Children under 6: A Tool for Proactive and Equitable Intervention. Int J Environ Res Public Health. 2021;18(5).
25. Balakrishnan P, Jones MR, Vaidya D, Tellez-Plaza M, Post WS, Kaufman JD, et al. Ethnic, Geographic, and Genetic Differences in Arsenic Metabolism at Low Arsenic Exposure: A Preliminary Analysis in the Multi-Ethnic Study of Atherosclerosis (MESA). Int J Environ Res Public Health. 2018;15(6).
26. Bangma J, Eaves LA, Oldenburg K, Reiner JL, Manuck T, Fry RC. Identifying Risk Factors for Levels of Per- and Polyfluoroalkyl Substances (PFAS) in the Placenta in a High-Risk Pregnancy Cohort in North Carolina. Environ Sci Technol. 2020;54(13):8158-66.
27. Barton KE, Starling AP, Higgins CP, McDonough CA, Calafat AM, Adgate JL. Sociodemographic and behavioral determinants of serum concentrations of per- and polyfluoroalkyl substances in a community highly exposed to aqueous film-forming foam contaminants in drinking water. Int J Hyg Environ Health. 2020;223(1):256-66.
28. Bastain TM, Chavez T, Habre R, Hernandez-Castro I, Grubbs B, Toledo-Corral CM, et al. Prenatal ambient air pollution and maternal depression at 12 months postpartum in the MADRES pregnancy cohort. Environ Health. 2021;20(1):121.
29. Basu R, Gavin L, Pearson D, Ebisu K, Malig B. Examining the Association Between Apparent Temperature and Mental Health-Related Emergency Room Visits in California. Am J Epidemiol. 2018;187(4):726-35.
30. Basu R, Rau R, Pearson D, Malig B. Temperature and Term Low Birth Weight in California. Am J Epidemiol. 2018;187(11):2306-14.
31. Bauza V, Madadi V, Ocharo R, Nguyen TH, Guest JS. Enteric pathogens from water, hands, surface, soil, drainage ditch, and stream exposure points in a low-income neighborhood of Nairobi, Kenya. Sci Total Environ. 2020;709:135344.
32. Benka-Coker ML, Clark ML, Rajkumar S, Young BN, Bachand AM, Balmes JR, et al. Exposure to Household Air Pollution from Biomass Cookstoves and Levels of Fractional Exhaled Nitric Oxide (FeNO) among Honduran Women. Int J Environ Res Public Health. 2018;15(11).
33. Berendes DM, de Mondesert L, Kirby AE, Yakubu H, Adomako L, Michiel J, et al. Variation in E. coli concentrations in open drains across neighborhoods in Accra, Ghana: The influence of onsite sanitation coverage and interconnectedness of urban environments. Int J Hyg Environ Health. 2020;224:113433.
34. Berger K, Eskenazi B, Kogut K, Parra K, Lustig RH, Greenspan LC, et al. Association of Prenatal Urinary Concentrations of Phthalates and Bisphenol A and Pubertal Timing in Boys and Girls. Environ Health Perspect. 2018;126(9):97004.
35. Bergmans RS, Larson P, Bennion E, Mezuk B, Wozniak MC, Steiner AL, et al. Short-term exposures to atmospheric evergreen, deciduous, grass, and ragweed aeroallergens and the risk of suicide in Ohio, 2007-2015: Exploring disparities by age, gender, and education level. Environ Res. 2021;200:111450.
36. Berman JD, Burkhardt J, Bayham J, Carter E, Wilson A. Acute Air Pollution Exposure and the Risk of Violent Behavior in the United States. Epidemiology. 2019;30(6):799-806.
37. Berman T, Barnett-Itzhaki Z, Axelrod R, Keinan-Boker L, Shimony T, Goldsmith R, et al. Socioeconomic inequalities in exposure to environmental tobacco smoke in children in Israel. Environ Int. 2018;121(Pt 1):643-8.
38. Bhatta DN, Glantz S. Parental tobacco use and child death: analysis of data from demographic and health surveys from South and South East Asian countries. Int J Epidemiol. 2019;48(1):199-206.
39. Bi C, Maestre JP, Li H, Zhang G, Givehchi R, Mahdavi A, et al. Phthalates and organophosphates in settled dust and HVAC filter dust of U.S. low-income homes: Association with season, building characteristics, and childhood asthma. Environ Int. 2018;121(Pt 1):916-30.
40. Binder AM, Corvalan C, Calafat AM, Ye X, Mericq V, Pereira A, et al. Childhood and adolescent phenol and phthalate exposure and the age of menarche in Latina girls. Environ Health. 2018;17(1):32.
41. Bloom MS, Commodore S, Ferguson PL, Neelon B, Pearce JL, Baumer A, et al. Association between gestational PFAS exposure and Children's adiposity in a diverse population. Environ Res. 2022;203:111820.
42. Bloom MS, Valachovic EL, Begum TF, Kucklick JR, Brock JW, Wenzel AG, et al. Association between gestational phthalate exposure and newborn head circumference; impacts by race and sex. Environ Res. 2021;195:110763.
43. Bloom MS, Wenzel AG, Brock JW, Kucklick JR, Wineland RJ, Cruze L, et al. Racial disparity in maternal phthalates exposure; Association with racial disparity in fetal growth and birth outcomes. Environ Int. 2019;127:473-86.
44. Bose-O'Reilly S, Lettmeier B, Shoko D, Roider G, Drasch G, Siebert U. Infants and mothers levels of mercury in breast milk, urine and hair, data from an artisanal and small-scale gold mining area in Kadoma / Zimbabwe. Environ Res. 2020;184:109266.
45. Bowe B, Xie Y, Gibson AK, Cai M, van Donkelaar A, Martin RV, et al. Ambient fine particulate matter air pollution and the risk of hospitalization among COVID-19 positive individuals: Cohort study. Environ Int. 2021;154:106564.
46. Bradatan C, Dennis JA, Flores-Yeffal N, Swain S. Child health, household environment, temperature and rainfall anomalies in Honduras: a socio-climate data linked analysis. Environ Health. 2020;19(1):10.
47. Bravo MA, Anthopolos R, Miranda ML. Characteristics of the built environment and spatial patterning of type 2 diabetes in the urban core of Durham, North Carolina. J Epidemiol Community Health. 2019;73(4):303-10.
48. Bravo MA, Miranda ML. Effects of accumulated environmental, social and host exposures on early childhood educational outcomes. Environ Res. 2021;198:111241.
49. Brown-Amilian S, Akolade Y. Disparities in COPD Hospitalizations: A Spatial Analysis of Proximity to Toxics Release Inventory Facilities in Illinois. Int J Environ Res Public Health. 2021;18(24).
50. Browning M, Rigolon A. Do Income, Race and Ethnicity, and Sprawl Influence the Greenspace-Human Health Link in City-Level Analyses? Findings from 496 Cities in the United States. Int J Environ Res Public Health. 2018;15(7).
51. Buck Louis GM, Zhai S, Smarr MM, Grewal J, Zhang C, Grantz KL, et al. Endocrine disruptors and neonatal anthropometry, NICHD Fetal Growth Studies - Singletons. Environ Int. 2018;119:515-26.
52. Buckley JP, Kim H, Wong E, Rebholz CM. Ultra-processed food consumption and exposure to phthalates and bisphenols in the US National Health and Nutrition Examination Survey, 2013-2014. Environ Int. 2019;131:105057.
53. Buthelezi SA, Kapwata T, Wernecke B, Webster C, Mathee A, Wright CY. Household Fuel Use for Heating and Cooking and Respiratory Health in a Low-Income, South African Coastal Community. Int J Environ Res Public Health. 2019;16(4).
54. Campbell SL, Remenyi TA, Williamson GJ, White CJ, Johnston FH. The Value of Local Heatwave Impact Assessment: A Case-Crossover Analysis of Hospital Emergency Department Presentations in Tasmania, Australia. Int J Environ Res Public Health. 2019;16(19).
55. Carrion D, Kaali S, Kinney PL, Owusu-Agyei S, Chillrud S, Yawson AK, et al. Examining the relationship between household air pollution and infant microbial nasal carriage in a Ghanaian cohort. Environ Int. 2019;133(Pt A):105150.
56. Casey JA, Cushing L, Depsky N, Morello-Frosch R. Climate Justice and California's Methane Superemitters: Environmental Equity Assessment of Community Proximity and Exposure Intensity. Environ Sci Technol. 2021;55(21):14746-57.
57. Casey JA, Karasek D, Ogburn EL, Goin DE, Dang K, Braveman PA, et al. Retirements of Coal and Oil Power Plants in California: Association With Reduced Preterm Birth Among Populations Nearby. Am J Epidemiol. 2018;187(8):1586-94.
58. Cassidy-Bushrow AE, Burmeister C, Lamerato L, Lemke LD, Mathieu M, O'Leary BF, et al. Prenatal airshed pollutants and preterm birth in an observational birth cohort study in Detroit, Michigan, USA. Environ Res. 2020;189:109845.
59. Chakraborty J. Convergence of COVID-19 and chronic air pollution risks: Racial/ethnic and socioeconomic inequities in the U.S. Environ Res. 2021;193:110586.
60. Chakraborty J, Basu P. Linking Industrial Hazards and Social Inequalities: Environmental Injustice in Gujarat, India. Int J Environ Res Public Health. 2018;16(1).
61. Chakraborty J, Basu P. Air Quality and Environmental Injustice in India: Connecting Particulate Pollution to Social Disadvantages. Int J Environ Res Public Health. 2021;18(1).
62. Chakraborty J, Collins TW, Grineski SE. Exploring the Environmental Justice Implications of Hurricane Harvey Flooding in Greater Houston, Texas. Am J Public Health. 2019;109(2):244-50.
63. Chen H, Burnett RT, Bai L, Kwong JC, Crouse DL, Lavigne E, et al. Residential Greenness and Cardiovascular Disease Incidence, Readmission, and Mortality. Environ Health Perspect. 2020;128(8):87005.
64. Chen Z, Huang BZ, Sidell MA, Chow T, Eckel SP, Pavlovic N, et al. Near-roadway air pollution associated with COVID-19 severity and mortality - Multiethnic cohort study in Southern California. Environ Int. 2021;157:106862.
65. Chevrier J, Rauch S, Crause M, Obida M, Gaspar F, Bornman R, et al. Associations of Maternal Exposure to Dichlorodiphenyltrichloroethane and Pyrethroids With Birth Outcomes Among Participants in the Venda Health Examination of Mothers, Babies and Their Environment Residing in an Area Sprayed for Malaria Control. Am J Epidemiol. 2019;188(1):130-40.
66. Chevrier J, Rauch S, Obida M, Crause M, Bornman R, Eskenazi B. Sex and poverty modify associations between maternal peripartum concentrations of DDT/E and pyrethroid metabolites and thyroid hormone levels in neonates participating in the VHEMBE study, South Africa. Environ Int. 2019;131:104958.
67. Chiang C, Pacyga DC, Strakovsky RS, Smith RL, James-Todd T, Williams PL, et al. Urinary phthalate metabolite concentrations and serum hormone levels in pre- and perimenopausal women from the Midlife Women's Health Study. Environ Int. 2021;156:106633.
68. Chiao C, Deji-Abiodun O. A global analysis of the regional variation in the symptoms of acute respiratory infection during childhood: Epidemics and their association with environmental vulnerability. Health Place. 2020;65:102400.
69. Chillrud SN, Ae-Ngibise KA, Gould CF, Owusu-Agyei S, Mujtaba M, Manu G, et al. The effect of clean cooking interventions on mother and child personal exposure to air pollution: results from the Ghana Randomized Air Pollution and Health Study (GRAPHS). J Expo Sci Environ Epidemiol. 2021;31(4):683-98.
70. Chiofalo JM, Golub M, Crump C, Calman N. Pediatric Blood Lead Levels Within New York City Public Versus Private Housing, 2003-2017. Am J Public Health. 2019;109(6):906-11.
71. Chiu YH, Williams PL, Gillman MW, Hauser R, Rifas-Shiman SL, Bellavia A, et al. Maternal intake of pesticide residues from fruits and vegetables in relation to fetal growth. Environ Int. 2018;119:421-8.
72. Christidis T, Erickson AC, Pappin AJ, Crouse DL, Pinault LL, Weichenthal SA, et al. Low concentrations of fine particle air pollution and mortality in the Canadian Community Health Survey cohort. Environ Health. 2019;18(1):84.
73. Chu MT, Gillooly SE, Levy JI, Vallarino J, Reyna LN, Cedeno Laurent JG, et al. Real-time indoor PM(2.5) monitoring in an urban cohort: Implications for exposure disparities and source control. Environ Res. 2021;193:110561.
74. Collins HN, Johnson PI, Calderon NM, Clark PY, Gillis AD, Le AM, et al. Differences in personal care product use by race/ethnicity among women in California: implications for chemical exposures. J Expo Sci Environ Epidemiol. 2021.
75. Collins TW, Grineski SE, Chakraborty J, Flores AB. Environmental injustice and Hurricane Harvey: A household-level study of socially disparate flood exposures in Greater Houston, Texas, USA. Environ Res. 2019;179(Pt A):108772.
76. Collins TW, Grineski SE, Nadybal S. Social disparities in exposure to noise at public schools in the contiguous United States. Environ Res. 2019;175:257-65.
77. Contreras JD, Trangucci R, Felix-Arellano EE, Rodriguez-Dozal S, Siebe C, Riojas-Rodriguez H, et al. Modeling Spatial Risk of Diarrheal Disease Associated with Household Proximity to Untreated Wastewater Used for Irrigation in the Mezquital Valley, Mexico. Environ Health Perspect. 2020;128(7):77002.
78. Coombs K, Taft D, Ward DV, Green BJ, Chew GL, Shamsaei B, et al. Variability of indoor fungal microbiome of green and non-green low-income homes in Cincinnati, Ohio. Sci Total Environ. 2018;610-611:212-8.
79. Corlin L, Ball S, Woodin M, Patton AP, Lane K, Durant JL, et al. Relationship of Time-Activity-Adjusted Particle Number Concentration with Blood Pressure. Int J Environ Res Public Health. 2018;15(9).
80. Credo J, Torkelson J, Rock T, Ingram JC. Quantification of Elemental Contaminants in Unregulated Water across Western Navajo Nation. Int J Environ Res Public Health. 2019;16(15).
81. Crouse DL, Balram A, Hystad P, Pinault L, van den Bosch M, Chen H, et al. Associations between Living Near Water and Risk of Mortality among Urban Canadians. Environ Health Perspect. 2018;126(7):077008.
82. Crouse DL, Pinault L, Balram A, Brauer M, Burnett RT, Martin RV, et al. Complex relationships between greenness, air pollution, and mortality in a population-based Canadian cohort. Environ Int. 2019;128:292-300.
83. Daniel D, Diener A, Pande S, Jansen S, Marks S, Meierhofer R, et al. Understanding the effect of socio-economic characteristics and psychosocial factors on household water treatment practices in rural Nepal using Bayesian Belief Networks. Int J Hyg Environ Health. 2019;222(5):847-55.
84. Daniel S, Balalian AA, Insel BJ, Liu X, Whyatt RM, Calafat AM, et al. Prenatal and early childhood exposure to phthalates and childhood behavior at age 7 years. Environ Int. 2020;143:105894.
85. Daniel S, Balalian AA, Whyatt RM, Liu X, Rauh V, Herbstman J, et al. Perinatal phthalates exposure decreases fine-motor functions in 11-year-old girls: Results from weighted Quantile sum regression. Environ Int. 2020;136:105424.
86. Daouda M, Henneman L, Kioumourtzoglou MA, Gemmill A, Zigler C, Casey J. Association between county-level coal-fired power plant pollution and racial disparities in preterm births from 2000 to 2018. Environ Res Lett. 2021;16(3).
87. Davis LF, Ramírez-Andreotta MD. Participatory research for environmental justice: a critical interpretive synthesis. Environ Health Perspect. 2021;129(2):026001.
88. De Roos AJ, Kenyon CC, Zhao Y, Moore K, Melly S, Hubbard RA, et al. Ambient daily pollen levels in association with asthma exacerbation among children in Philadelphia, Pennsylvania. Environ Int. 2020;145:106138.
89. Deguen S, Ahlers N, Gilles M, Danzon A, Carayol M, Zmirou-Navier D, et al. Using a Clustering Approach to Investigate Socio-Environmental Inequality in Preterm Birth-A Study Conducted at Fine Spatial Scale in Paris (France). Int J Environ Res Public Health. 2018;15(9).
90. Dehom S, Knutsen S, Bahjri K, Shavlik D, Oda K, Ali H, et al. Racial Difference in the Association of Long-Term Exposure to Fine Particulate Matter (PM(2.5)) and Cardiovascular Disease Mortality among Renal Transplant Recipients. Int J Environ Res Public Health. 2021;18(8).
91. Demetillo MAG, Navarro A, Knowles KK, Fields KP, Geddes JA, Nowlan CR, et al. Observing Nitrogen Dioxide Air Pollution Inequality Using High-Spatial-Resolution Remote Sensing Measurements in Houston, Texas. Environ Sci Technol. 2020;54(16):9882-95.
92. Deng Q, Deng L, Lu C, Li Y, Norback D. Parental stress and air pollution increase childhood asthma in China. Environ Res. 2018;165:23-31.
93. Dialesandro J, Brazil N, Wheeler S, Abunnasr Y. Dimensions of Thermal Inequity: Neighborhood Social Demographics and Urban Heat in the Southwestern U.S. Int J Environ Res Public Health. 2021;18(3).
94. Dietrich M, Wolfe A, Burke M, Krekeler MPS. The first pollution investigation of road sediment in Gary, Indiana: Anthropogenic metals and possible health implications for a socioeconomically disadvantaged area. Environ Int. 2019;128:175-92.
95. Ding N, Harlow SD, Batterman S, Mukherjee B, Park SK. Longitudinal trends in perfluoroalkyl and polyfluoroalkyl substances among multiethnic midlife women from 1999 to 2011: The Study of Women's Health Across the Nation. Environ Int. 2020;135:105381.
96. do Nascimento FP, de Almeida MF, Gouveia N. Individual and contextual socioeconomic status as effect modifier in the air pollution-birth outcome association. Sci Total Environ. 2022;803:149790.
97. Dobraca D, Lum R, Sjodin A, Calafat AM, Laurent CA, Kushi LH, et al. Urinary biomarkers of polycyclic aromatic hydrocarbons in pre- and peri-pubertal girls in Northern California: Predictors of exposure and temporal variability. Environ Res. 2018;165:46-54.
98. Dodson RE, Cardona B, Zota AR, Robinson Flint J, Navarro S, Shamasunder B. Personal care product use among diverse women in California: Taking Stock Study. J Expo Sci Environ Epidemiol. 2021;31(3):487-502.
99. Dos Santos NR, Rodrigues JLG, Bandeira MJ, Anjos A, Araujo CFS, Adan LFF, et al. Manganese exposure and association with hormone imbalance in children living near a ferro-manganese alloy plant. Environ Res. 2019;172:166-74.
100. Driver A, Mehdizadeh C, Bara-Garcia S, Bodenreider C, Lewis J, Wilson S. Utilization of the Maryland Environmental Justice Screening Tool: A Bladensburg, Maryland Case Study. Int J Environ Res Public Health. 2019;16(3).
101. Egan KB, Cornwell CR, Courtney JG, Ettinger AS. Blood Lead Levels in U.S. Children Ages 1-11 Years, 1976-2016. Environ Health Perspect. 2021;129(3):37003.
102. Egendorf SP, Mielke HW, Castorena-Gonzalez JA, Powell ET, Gonzales CR. Soil Lead (Pb) in New Orleans: A Spatiotemporal and Racial Analysis. Int J Environ Res Public Health. 2021;18(3).
103. Eggers MJ, Doyle JT, Lefthand MJ, Young SL, Moore-Nall AL, Kindness L, et al. Community Engaged Cumulative Risk Assessment of Exposure to Inorganic Well Water Contaminants, Crow Reservation, Montana. Int J Environ Res Public Health. 2018;15(1).
104. Eick SM, Ferreccio C, Acevedo J, Castriota F, Cordero JF, Roh T, et al. Socioeconomic status and the association between arsenic exposure and type 2 diabetes. Environ Res. 2019;172:578-85.
105. Eisenberg A, Seymour E, Hill AB, Akers J. Toxic structures: Speculation and lead exposure in Detroit's single-family rental market. Health Place. 2020;64:102390.
106. Ekenga CC, Yeung CY, Oka M. Cancer risk from air toxics in relation to neighborhood isolation and sociodemographic characteristics: A spatial analysis of the St. Louis metropolitan area, USA. Environ Res. 2019;179(Pt B):108844.
107. Elf JL, Kinikar A, Khadse S, Mave V, Suryavanshi N, Gupte N, et al. Sources of household air pollution and their association with fine particulate matter in low-income urban homes in India. J Expo Sci Environ Epidemiol. 2018;28(4):400-10.
108. Elford S, Adams MD. Associations between socioeconomic status and ultrafine particulate exposure in the school commute: An environmental inequality study for Toronto, Canada. Environ Res. 2021;192:110224.
109. Enders C, Pearson D, Harley K, Ebisu K. Exposure to coarse particulate matter during gestation and term low birthweight in California: Variation in exposure and risk across region and socioeconomic subgroup. Sci Total Environ. 2019;653:1435-44.
110. Fabisiak JP, Jackson EM, Brink LL, Presto AA. A risk-based model to assess environmental justice and coronary heart disease burden from traffic-related air pollutants. Environ Health. 2020;19(1):34.
111. Fan S, Xue Z, Yuan J, Zhou Z, Wang Y, Yang Z, et al. Associations of Residential Greenness with Diabetes Mellitus in Chinese Uyghur Adults. Int J Environ Res Public Health. 2019;16(24).
112. Fawkes L, Sansom G. Preliminary Study of Lead-Contaminated Drinking Water in Public Parks-An Assessment of Equity and Exposure Risks in Two Texas Communities. Int J Environ Res Public Health. 2021;18(12).
113. Feng S, Chen L, Sun R, Feng Z, Li J, Khan MS, et al. The Distribution and Accessibility of Urban Parks in Beijing, China: Implications of Social Equity. Int J Environ Res Public Health. 2019;16(24).
114. Fishe J, Zheng Y, Lyu T, Bian J, Hu H. Environmental effects on acute exacerbations of respiratory diseases: A real-world big data study. Sci Total Environ. 2022;806(Pt 1):150352.
115. Flanagan E, Stroh E, Oudin A, Malmqvist E. Connecting Air Pollution Exposure to Socioeconomic Status: A Cross-Sectional Study on Environmental Injustice among Pregnant Women in Scania, Sweden. Int J Environ Res Public Health. 2019;16(24).
116. Foley K, Ferencz L, Meghea C, Abram Z, Penzes M, Fogarasi-Grenczer A, et al. Home- and Car-Based Rules in Foster Care Settings to Reduce Exposure to Secondhand Smoke: Before and after Romanian National Clean Air Legislation. Int J Environ Res Public Health. 2018;15(8).
117. Fong KC, Bell ML. Do fine particulate air pollution (PM(2.5)) exposure and its attributable premature mortality differ for immigrants compared to those born in the United States? Environ Res. 2021;196:110387.
118. Fong KC, Kloog I, Coull BA, Koutrakis P, Laden F, Schwartz JD, et al. Residential Greenness and Birthweight in the State of Massachusetts, USA. Int J Environ Res Public Health. 2018;15(6).
119. Fong KC, Mehta NK, Bell ML. Disparities in exposure to surrounding greenness related to proportion of the population that were immigrants to the United States. Int J Hyg Environ Health. 2020;224:113434.
120. Fontan-Vela M, Rivera-Navarro J, Gullon P, Diez J, Anguelovski I, Franco M. Active use and perceptions of parks as urban assets for physical activity: A mixed-methods study. Health Place. 2021;71:102660.
121. Frediani JK, Naioti EA, Vos MB, Figueroa J, Marsit CJ, Welsh JA. Arsenic exposure and risk of nonalcoholic fatty liver disease (NAFLD) among U.S. adolescents and adults: an association modified by race/ethnicity, NHANES 2005-2014. Environ Health. 2018;17(1):6.
122. Gaston SA, James-Todd T, Harmon Q, Taylor KW, Baird D, Jackson CL. Chemical/straightening and other hair product usage during childhood, adolescence, and adulthood among African-American women: potential implications for health. J Expo Sci Environ Epidemiol. 2020;30(1):86-96.
123. Gaston SA, James-Todd T, Riley NM, Gladney MN, Harmon QE, Baird DD, et al. Hair Maintenance and Chemical Hair Product Usage as Barriers to Physical Activity in Childhood and Adulthood among African American Women. Int J Environ Res Public Health. 2020;17(24).
124. Gearhart-Serna LM, Tacam M, Jr., Slotkin TA, Devi GR. Analysis of polycyclic aromatic hydrocarbon intake in the US adult population from NHANES 2005-2014 identifies vulnerable subpopulations, suggests interaction between tobacco smoke exposure and sociodemographic factors. Environ Res. 2021;201:111614.
125. Ghosh R, Haque M, Turner PC, Cruz-Cano R, Dallal CM. Racial and Sex Differences between Urinary Phthalates and Metabolic Syndrome among U.S. Adults: NHANES 2005-2014. Int J Environ Res Public Health. 2021;18(13).
126. Gleason JA, Nanavaty JV, Fagliano JA. Drinking water lead and socioeconomic factors as predictors of blood lead levels in New Jersey's children between two time periods. Environ Res. 2019;169:409-16.
127. Grajeda LM, Thompson LM, Arriaga W, Canuz E, Omer SB, Sage M, et al. Effectiveness of Gas and Chimney Biomass Stoves for Reducing Household Air Pollution Pregnancy Exposure in Guatemala: Sociodemographic Effect Modifiers. Int J Environ Res Public Health. 2020;17(21).
128. Grazuleviciene R, Andrusaityte S, Grazulevicius T, Dedele A. Neighborhood Social and Built Environment and Disparities in the Risk of Hypertension: A Cross-Sectional Study. Int J Environ Res Public Health. 2020;17(20).
129. Grineski SE, Collins TW. Geographic and social disparities in exposure to air neurotoxicants at U.S. public schools. Environ Res. 2018;161:580-7.
130. Grineski SE, T WC, Rubio R. Distributional Environmental Injustices for a Minority Group without Minority Status: Arab Americans and Residential Exposure to Carcinogenic Air Pollution in the US. Int J Environ Res Public Health. 2019;16(24).
131. Groot J, Keller A, Pedersen M, Sigsgaard T, Loft S, Nybo Andersen AM. Indoor home environments of Danish children and the socioeconomic position and health of their parents: A descriptive study. Environ Int. 2022;160:107059.
132. Guo H, Chang Z, Wu J, Li W. Air pollution and lung cancer incidence in China: Who are faced with a greater effect? Environ Int. 2019;132:105077.
133. Guo H, Li W, Yao F, Wu J, Zhou X, Yue Y, et al. Who are more exposed to PM2.5 pollution: A mobile phone data approach. Environ Int. 2020;143:105821.
134. Guo H, Wei J, Li X, Ho HC, Song Y, Wu J, et al. Do socioeconomic factors modify the effects of PM1 and SO2 on lung cancer incidence in China? Sci Total Environ. 2021;756:143998.
135. Hall J, Galarraga J, Berman I, Edwards C, Khanjar N, Kavi L, et al. Environmental Injustice and Industrial Chicken Farming in Maryland. Int J Environ Res Public Health. 2021;18(21).
136. Han C, Xu R, Gao CX, Yu W, Zhang Y, Han K, et al. Socioeconomic disparity in the association between long-term exposure to PM(2.5) and mortality in 2640 Chinese counties. Environ Int. 2021;146:106241.
137. Hasan MM, Alam K. Inequality in access to improved drinking water sources and childhood diarrhoea in low- and middle-income countries. Int J Hyg Environ Health. 2020;226:113493.
138. Heggeseth BC, Holland N, Eskenazi B, Kogut K, Harley KG. Heterogeneity in childhood body mass trajectories in relation to prenatal phthalate exposure. Environ Res. 2019;175:22-33.
139. Henderson E, Continente X, Fernandez E, Tigova O, Cortes-Francisco N, Gallus S, et al. Secondhand smoke exposure in outdoor children's playgrounds in 11 European countries. Environ Int. 2021;149:105775.
140. Hicks DJ. Census Demographics and Chlorpyrifos Use in California's Central Valley, 2011-15: A Distributional Environmental Justice Analysis. Int J Environ Res Public Health. 2020;17(7).
141. Hobbs M, Wade A, Jones P, Marek L, Tomintz M, Sharma K, et al. Area-level deprivation, childhood dental ambulatory sensitive hospitalizations and community water fluoridation: evidence from New Zealand. Int J Epidemiol. 2020;49(3):908-16.
142. Holcomb DA, Knee J, Sumner T, Adriano Z, de Bruijn E, Nala R, et al. Human fecal contamination of water, soil, and surfaces in households sharing poor-quality sanitation facilities in Maputo, Mozambique. Int J Hyg Environ Health. 2020;226:113496.
143. Hoshiko S, Pearl M, Yang J, Aldous KM, Roeseler A, Dominguez ME, et al. Differences in Prenatal Tobacco Exposure Patterns among 13 Race/Ethnic Groups in California. Int J Environ Res Public Health. 2019;16(3).
144. Hu K, Guo Y, Hochrainer-Stigler S, Liu W, See L, Yang X, et al. Evidence for Urban-Rural Disparity in Temperature-Mortality Relationships in Zhejiang Province, China. Environ Health Perspect. 2019;127(3):37001.
145. Huang G, Zhou W, Qian Y, Fisher B. Breathing the same air? Socioeconomic disparities in PM(2.5) exposure and the potential benefits from air filtration. Sci Total Environ. 2019;657:619-26.
146. Huang H, Wei Y, Xia Y, Wei L, Chen X, Zhang R, et al. Child marriage, maternal serum metal exposure, and risk of preterm birth in rural Bangladesh: evidence from mediation analysis. J Expo Sci Environ Epidemiol. 2021;31(3):571-80.
147. Huang H, Woodruff TJ, Baer RJ, Bangia K, August LM, Jellife-Palowski LL, et al. Investigation of association between environmental and socioeconomic factors and preterm birth in California. Environ Int. 2018;121(Pt 2):1066-78.
148. Humphrey JL, Barton KE, Man Shrestha P, Carlton EJ, Newman LS, Dowling Root E, et al. Air infiltration in low-income, urban homes and its relationship to lung function. J Expo Sci Environ Epidemiol. 2020;30(2):262-70.
149. Humphrey JL, Lindstrom M, Barton KE, Shrestha PM, Carlton EJ, Adgate JL, et al. Social and Environmental Neighborhood Typologies and Lung Function in a Low-Income, Urban Population. Int J Environ Res Public Health. 2019;16(7).
150. Humphrey JL, Reid CE, Kinnee EJ, Kubzansky LD, Robinson LF, Clougherty JE. Putting Co-Exposures on Equal Footing: An Ecological Analysis of Same-Scale Measures of Air Pollution and Social Factors on Cardiovascular Disease in New York City. Int J Environ Res Public Health. 2019;16(23).
151. Hyland C, Bradshaw P, Deardorff J, Gunier RB, Mora AM, Kogut K, et al. Interactions of agricultural pesticide use near home during pregnancy and adverse childhood experiences on adolescent neurobehavioral development in the CHAMACOS study. Environ Res. 2022;204(Pt A):111908.
152. Hyland C, Bradshaw PT, Gunier RB, Mora AM, Kogut K, Deardorff J, et al. Associations between pesticide mixtures applied near home during pregnancy and early childhood with adolescent behavioral and emotional problems in the CHAMACOS study. Environ Epidemiol. 2021;5(3):e150.
153. Ish J, Symanski E, Whitworth KW. Exploring Disparities in Maternal Residential Proximity to Unconventional Gas Development in the Barnett Shale in North Texas. Int J Environ Res Public Health. 2019;16(3).
154. Iyer HS, Valeri L, James P, Chen JT, Hart JE, Laden F, et al. The contribution of residential greenness to mortality among men with prostate cancer: a registry-based cohort study of Black and White men. Environ Epidemiol. 2020;4(2):e087.
155. James-Todd T, Connolly L, Preston EV, Quinn MR, Plotan M, Xie Y, et al. Hormonal activity in commonly used Black hair care products: evaluating hormone disruption as a plausible contribution to health disparities. J Expo Sci Environ Epidemiol. 2021;31(3):476-86.
156. Johnson Gaither C, Afrin S, Garcia-Menendez F, Odman MT, Huang R, Goodrick S, et al. African American Exposure to Prescribed Fire Smoke in Georgia, USA. Int J Environ Res Public Health. 2019;16(17).
157. Johnston JE, Chau K, Franklin M, Cushing L. Environmental Justice Dimensions of Oil and Gas Flaring in South Texas: Disproportionate Exposure among Hispanic communities. Environ Sci Technol. 2020;54(10):6289-98.
158. Johnston JE, Enebish T, Eckel SP, Navarro S, Shamasunder B. Respiratory health, pulmonary function and local engagement in urban communities near oil development. Environ Res. 2021;197:111088.
159. Jones MR, Tellez-Plaza M, Vaidya D, Grau-Perez M, Post WS, Kaufman JD, et al. Ethnic, geographic and dietary differences in arsenic exposure in the multi-ethnic study of atherosclerosis (MESA). J Expo Sci Environ Epidemiol. 2019;29(3):310-22.
160. Jorgenson AK, Thombs RP, Clark B, Givens JE, Hill TD, Huang X, et al. Inequality amplifies the negative association between life expectancy and air pollution: A cross-national longitudinal study. Sci Total Environ. 2021;758:143705.
161. Juarez PD, Tabatabai M, Valdez RB, Hood DB, Im W, Mouton C, et al. The Effects of Social, Personal, and Behavioral Risk Factors and PM(2.5) on Cardio-Metabolic Disparities in a Cohort of Community Health Center Patients. Int J Environ Res Public Health. 2020;17(10).
162. Jukic AMZ, Kim SS, Meeker JD, Weiss ST, Cantonwine DE, McElrath TF, et al. A prospective study of maternal 25-hydroxyvitamin D (25OHD) in the first trimester of pregnancy and second trimester heavy metal levels. Environ Res. 2021;199:111351.
163. Jung J, Uejio CK, Adeyeye TE, Kintziger KW, Duclos C, Reid K, et al. Using social security number to identify sub-populations vulnerable to the health impacts from extreme heat in Florida, U.S. Environ Res. 2021;202:111738.
164. Kaiser ML, Hand MD, Pence EK. Individual and Community Engagement in Response to Environmental Challenges Experienced in Four Low-Income Urban Neighborhoods. Int J Environ Res Public Health. 2020;17(6).
165. Kazemiparkouhi F, Honda T, Eum KD, Wang B, Manjourides J, Suh HH. The impact of Long-Term PM(2.5) constituents and their sources on specific causes of death in a US Medicare cohort. Environ Int. 2022;159:106988.
166. Kehm RD, Oskar S, Tehranifar P, Zeinomar N, Rundle AG, Herbstman JB, et al. Associations of prenatal exposure to polycyclic aromatic hydrocarbons with pubertal timing and body composition in adolescent girls: Implications for breast cancer risk. Environ Res. 2021;196:110369.
167. Keidel D, Anto JM, Basagana X, Bono R, Burte E, Carsin AE, et al. The Role of Socioeconomic Status in the Association of Lung Function and Air Pollution-A Pooled Analysis of Three Adult ESCAPE Cohorts. Int J Environ Res Public Health. 2019;16(11).
168. Khabo-Mmekoa CMN, Momba MNB. The Impact of Social Disparities on Microbiological Quality of Drinking Water Supply in Ugu District Municipality of Kwazulu-Natal Province, South Africa. Int J Environ Res Public Health. 2019;16(16).
169. Kim E, Kwon HJ, Ha M, Lim JA, Lim MH, Yoo SJ, et al. How Does Low Socioeconomic Status Increase Blood Lead Levelsin KoreanChildren? Int J Environ Res Public Health. 2018;15(7).
170. Kim K, Jung J, Schollaert C, Spector JT. A Comparative Assessment of Cooling Center Preparedness across Twenty-Five U.S. Cities. Int J Environ Res Public Health. 2021;18(9).
171. Kingsley SL, Eliot MN, Kelsey KT, Calafat AM, Ehrlich S, Lanphear BP, et al. Variability and predictors of serum perfluoroalkyl substance concentrations during pregnancy and early childhood. Environ Res. 2018;165:247-57.
172. Kirwa K, Feric Z, Manjourides J, Alshawabekeh A, Vega CMV, Cordero JF, et al. Preterm birth and PM(2.5) in Puerto Rico: evidence from the PROTECT birth cohort. Environ Health. 2021;20(1):69.
173. Klompmaker JO, Hart JE, Holland I, Sabath MB, Wu X, Laden F, et al. County-level exposures to greenness and associations with COVID-19 incidence and mortality in the United States. Environ Res. 2021;199:111331.
174. Knapp M, Gustat J, Darensbourg R, Myers L, Johnson C. The Relationships between Park Quality, Park Usage, and Levels of Physical Activity in Low-Income, African American Neighborhoods. Int J Environ Res Public Health. 2018;16(1).
175. Knobel P, Kondo M, Maneja R, Zhao Y, Dadvand P, Schinasi LH. Associations of objective and perceived greenness measures with cardiovascular risk factors in Philadelphia, PA: A spatial analysis. Environ Res. 2021;197:110990.
176. Koman PD, Romo F, Swinton P, Mentz GB, de Majo RF, Sampson NR, et al. MI-Environment: Geospatial patterns and inequality of relative heat stress vulnerability in Michigan. Health Place. 2019;60:102228.
177. Kong YL, Anis-Syakira J, Fun WH, Balqis-Ali NZ, Shakirah MS, Sararaks S. Socio-Economic Factors Related to Drinking Water Source and Sanitation in Malaysia. Int J Environ Res Public Health. 2020;17(21).
178. Konkle SL, Zierold KM, Taylor KC, Riggs DW, Bhatnagar A. National secular trends in ambient air volatile organic compound levels and biomarkers of exposure in the United States. Environ Res. 2020;182:108991.
179. Korn A, Bolton SM, Spencer B, Alarcon JA, Andrews L, Voss JG. Physical and Mental Health Impacts of Household Gardens in an Urban Slum in Lima, Peru. Int J Environ Res Public Health. 2018;15(8).
180. Kravitz-Wirtz N, Teixeira S, Hajat A, Woo B, Crowder K, Takeuchi D. Early-Life Air Pollution Exposure, Neighborhood Poverty, and Childhood Asthma in the United States, 1990(-)2014. Int J Environ Res Public Health. 2018;15(6).
181. Kulick ER, Elkind MSV, Boehme AK, Joyce NR, Schupf N, Kaufman JD, et al. Long-term exposure to ambient air pollution, APOE-epsilon4 status, and cognitive decline in a cohort of older adults in northern Manhattan. Environ Int. 2020;136:105440.
182. Kulinkina AV, Sodipo MO, Schultes OL, Osei BG, Agyapong EA, Egorov AI, et al. Rural Ghanaian households are more likely to use alternative unimproved water sources when water from boreholes has undesirable organoleptic characteristics. Int J Hyg Environ Health. 2020;227:113514.
183. Lamichhane DK, Lee SY, Ahn K, Kim KW, Shin YH, Suh DI, et al. Quantile regression analysis of the socioeconomic inequalities in air pollution and birth weight. Environ Int. 2020;142:105875.
184. Latham S, Jennings JL. Reducing lead exposure in school water: Evidence from remediation efforts in New York City public schools. Environ Res. 2022;203:111735.
185. Lee H, Myung W, Kim SE, Kim DK, Kim H. Ambient air pollution and completed suicide in 26 South Korean cities: Effect modification by demographic and socioeconomic factors. Sci Total Environ. 2018;639:944-51.
186. Leijssen JB, Snijder MB, Timmermans EJ, Generaal E, Stronks K, Kunst AE. The association between road traffic noise and depressed mood among different ethnic and socioeconomic groups. The HELIUS study. Int J Hyg Environ Health. 2019;222(2):221-9.
187. Leuenberger A, Cambaco O, Zabre HR, Lyatuu I, Utzinger J, Munguambe K, et al. "It Is Like We Are Living in a Different World": Health Inequity in Communities Surrounding Industrial Mining Sites in Burkina Faso, Mozambique, and Tanzania. Int J Environ Res Public Health. 2021;18(21).
188. Li B, Xiao D. The Impact of Income Inequality on Subjective Environmental Pollution: Individual Evidence from China. Int J Environ Res Public Health. 2021;18(15).
189. Li C, Bloom MS, Lin S, Ren M, Hajat S, Wang Q, et al. Temperature variation and preterm birth among live singleton deliveries in Shenzhen, China: A time-to-event analysis. Environ Res. 2021;195:110834.
190. Li H, Hart JE, Mahalingaiah S, Nethery RC, James P, Bertone-Johnson E, et al. Associations of long-term exposure to environmental noise and outdoor light at night with age at natural menopause in a US women cohort. Environ Epidemiol. 2021;5(3):e154.
191. Li P, Jing J, Guo W, Guo X, Hu W, Qi X, et al. The associations of air pollution and socioeconomic factors with esophageal cancer in China based on a spatiotemporal analysis. Environ Res. 2021;196:110415.
192. Li R, Hou J, Tu R, Liu X, Zuo T, Dong X, et al. Associations of mixture of air pollutants with estimated 10-year atherosclerotic cardiovascular disease risk modified by socio-economic status: The Henan Rural Cohort Study. Sci Total Environ. 2021;793:148542.
193. Li Y, Akkus C, Yu X, Joyner A, Kmet J, Sweat D, et al. Heatwave Events and Mortality Outcomes in Memphis, Tennessee: Testing Effect Modification by Socioeconomic Status and Urbanicity. Int J Environ Res Public Health. 2019;16(22).
194. Liddell JL, Kington SG. "Something Was Attacking Them and Their Reproductive Organs": Environmental Reproductive Justice in an Indigenous Tribe in the United States Gulf Coast. Int J Environ Res Public Health. 2021;18(2).
195. Lieberman-Cribbin W, Gillezeau C, Schwartz RM, Taioli E. Unequal social vulnerability to Hurricane Sandy flood exposure. J Expo Sci Environ Epidemiol. 2021;31(5):804-9.
196. Lieberman-Cribbin W, Liu B, Sheffield P, Schwartz R, Taioli E. Socioeconomic disparities in incidents at toxic sites during Hurricane Harvey. J Expo Sci Environ Epidemiol. 2021;31(3):454-60.
197. Lievanos RS. Racialized Structural Vulnerability: Neighborhood Racial Composition, Concentrated Disadvantage, and Fine Particulate Matter in California. Int J Environ Res Public Health. 2019;16(17).
198. Liévanos RS, Evans CR, Light R. An Intercategorical Ecology of Lead Exposure: Complex Environmental Health Vulnerabilities in the Flint Water Crisis. Int J Environ Res Public Health. 2021;18(5).
199. Lin PD, Cardenas A, Hauser R, Gold DR, Kleinman KP, Hivert MF, et al. Temporal trends of concentrations of per- and polyfluoroalkyl substances among adults with overweight and obesity in the United States: Results from the Diabetes Prevention Program and NHANES. Environ Int. 2021;157:106789.
200. Lipfert FW, Wyzga RE. Environmental predictors of survival in a cohort of U.S. military veterans: A multi-level spatio-temporal analysis stratified by race. Environ Res. 2020;183:108842.
201. Liu H, Bai X, Pang X. Intercity variability and local factors influencing the level of pesticide residues in marketed fruits and vegetables of China. Sci Total Environ. 2020;700:134481.
202. Liu J, Clark LP, Bechle MJ, Hajat A, Kim SY, Robinson AL, et al. Disparities in Air Pollution Exposure in the United States by Race/Ethnicity and Income, 1990-2010. Environ Health Perspect. 2021;129(12):127005.
203. Liu J, Li Q, Gu W, Wang C. The Impact of Consumption Patterns on the Generation of Municipal Solid Waste in China: Evidences from Provincial Data. Int J Environ Res Public Health. 2019;16(10).
204. Liu J, Portnoy J, Raine A, Gladieux M, McGarry P, Chen A. Blood lead levels mediate the relationship between social adversity and child externalizing behavior. Environ Res. 2022;204(Pt D):112396.
205. Liu M, Tang W, Zhang Y, Wang Y, Baima K, Li Y, et al. Urban-rural differences in the association between long-term exposure to ambient air pollution and obesity in China. Environ Res. 2021;201:111597.
206. Liu X, Taylor MP, Aelion CM, Dong C. Novel Application of Machine Learning Algorithms and Model-Agnostic Methods to Identify Factors Influencing Childhood Blood Lead Levels. Environ Sci Technol. 2021;55(19):13387-99.
207. Loftus CT, Bush NR, Day DB, Ni Y, Tylavsky FA, Karr CJ, et al. Exposure to prenatal phthalate mixtures and neurodevelopment in the Conditions Affecting Neurocognitive Development and Learning in Early childhood (CANDLE) study. Environ Int. 2021;150:106409.
208. Loftus CT, Ni Y, Szpiro AA, Hazlehurst MF, Tylavsky FA, Bush NR, et al. Exposure to ambient air pollution and early childhood behavior: A longitudinal cohort study. Environ Res. 2020;183:109075.
209. Loizeau M, Buteau S, Chaix B, McElroy S, Counil E, Benmarhnia T. Does the air pollution model influence the evidence of socio-economic disparities in exposure and susceptibility? Environ Res. 2018;167:650-61.
210. Loustaunau MG, Chakraborty J. Vehicular Air Pollution in Houston, Texas: An Intra-Categorical Analysis of Environmental Injustice. Int J Environ Res Public Health. 2019;16(16).
211. Lucchini RG, Guazzetti S, Renzetti S, Conversano M, Cagna G, Fedrighi C, et al. Neurocognitive impact of metal exposure and social stressors among schoolchildren in Taranto, Italy. Environ Health. 2019;18(1):67.
212. Luo Q, Zhang M, Yao W, Fu Y, Wei H, Tao Y, et al. A Spatio-Temporal Pattern and Socio-Economic Factors Analysis of Improved Sanitation in China, 2006(-)2015. Int J Environ Res Public Health. 2018;15(11).
213. Ma X, Longley I, Gao J, Salmond J. Evaluating the Effect of Ambient Concentrations, Route Choices, and Environmental (in)Justice on Students' Dose of Ambient NO(2) While Walking to School at Population Scales. Environ Sci Technol. 2020;54(20):12908-19.
214. Madrigano J, Lane K, Petrovic N, Ahmed M, Blum M, Matte T. Awareness, Risk Perception, and Protective Behaviors for Extreme Heat and Climate Change in New York City. Int J Environ Res Public Health. 2018;15(7).
215. Malecki KMC, Schultz AA, Bergmans RS. Neighborhood Perceptions and Cumulative Impacts of Low Level Chronic Exposure to Fine Particular Matter (PM(2.5)) on Cardiopulmonary Health. Int J Environ Res Public Health. 2018;15(1).
216. Malig BJ, Wu XM, Guirguis K, Gershunov A, Basu R. Associations between ambient temperature and hepatobiliary and renal hospitalizations in California, 1999 to 2009. Environ Res. 2019;177:108566.
217. Marcillo C, Krometis LA, Krometis J. Approximating Community Water System Service Areas to Explore the Demographics of SDWA Compliance in Virginia. Int J Environ Res Public Health. 2021;18(24).
218. Martin C, Simonds VW, Young SL, Doyle J, Lefthand M, Eggers MJ. Our Relationship to Water and Experience of Water Insecurity among Apsaalooke (Crow Indian) People, Montana. Int J Environ Res Public Health. 2021;18(2).
219. Masri S, LeBron A, Logue M, Valencia E, Ruiz A, Reyes A, et al. Social and spatial distribution of soil lead concentrations in the City of Santa Ana, California: Implications for health inequities. Sci Total Environ. 2020;743:140764.
220. Masri S, Scaduto E, Jin Y, Wu J. Disproportionate Impacts of Wildfires among Elderly and Low-Income Communities in California from 2000-2020. Int J Environ Res Public Health. 2021;18(8).
221. McDonald JA, Tehranifar P, Flom JD, Terry MB, James-Todd T. Hair product use, age at menarche and mammographic breast density in multiethnic urban women. Environ Health. 2018;17(1):1.
222. McDonald YJ, Jones NE. Drinking Water Violations and Environmental Justice in the United States, 2011-2015. Am J Public Health. 2018;108(10):1401-7.
223. Mehta SS, Applebaum KM, James-Todd T, Coleman-Phox K, Adler N, Laraia B, et al. Associations between sociodemographic characteristics and exposures to PBDEs, OH-PBDEs, PCBs, and PFASs in a diverse, overweight population of pregnant women. J Expo Sci Environ Epidemiol. 2020;30(1):42-55.
224. Mekonnen ZK, Oehlert JW, Eskenazi B, Shaw GM, Balmes JR, Padula AM. The relationship between air pollutants and maternal socioeconomic factors on preterm birth in California urban counties. J Expo Sci Environ Epidemiol. 2021;31(3):503-13.
225. Mikati I, Benson AF, Luben TJ, Sacks JD, Richmond-Bryant J. Disparities in Distribution of Particulate Matter Emission Sources by Race and Poverty Status. Am J Public Health. 2018;108(4):480-5.
226. Milando CW, Yitshak-Sade M, Zanobetti A, Levy JI, Laden F, Fabian MP. Modeling the impact of exposure reductions using multi-stressor epidemiology, exposure models, and synthetic microdata: an application to birthweight in two environmental justice communities. J Expo Sci Environ Epidemiol. 2021;31(3):442-53.
227. Min E, Piazza M, Galaviz VE, Saganic E, Schmeltz M, Freelander L, et al. Quantifying the Distribution of Environmental Health Threats and Hazards in Washington State Using a Cumulative Environmental Inequality Index. Environ Justice. 2021;14(4):298-314.
228. Mitchell BC, Chakraborty J, Basu P. Social Inequities in Urban Heat and Greenspace: Analyzing Climate Justice in Delhi, India. Int J Environ Res Public Health. 2021;18(9).
229. Mitro SD, Chu MT, Dodson RE, Adamkiewicz G, Chie L, Brown FM, et al. Phthalate metabolite exposures among immigrants living in the United States: findings from NHANES, 1999–2014. J Expo Sci Environ Epidemiol. 2019;29(1):71-82.
230. Montresor-Lopez JA, Reading SR, Yanosky JD, Mittleman MA, Bell RA, Crume TL, et al. The relationship between traffic-related air pollution exposures and allostatic load score among youth with type 1 diabetes in the SEARCH cohort. Environ Res. 2021;197:111075.
231. Moody HA, Grady SC. Lead Emissions and Population Vulnerability in the Detroit Metropolitan Area, 2006-2013: Impact of Pollution, Housing Age and Neighborhood Racial Isolation and Poverty on Blood Lead in Children. Int J Environ Res Public Health. 2021;18(5).
232. Mousavi A, Yuan Y, Masri S, Barta G, Wu J. Impact of 4th of July Fireworks on Spatiotemporal PM(2.5) Concentrations in California Based on the PurpleAir Sensor Network: Implications for Policy and Environmental Justice. Int J Environ Res Public Health. 2021;18(11).
233. Mperejekumana P, Li H, Wu R, Lu J, Tursunov O, Elshareef H, et al. Determinants of Household Energy Choice for Cooking in Northern Sudan: A Multinomial Logit Estimation. Int J Environ Res Public Health. 2021;18(21).
234. Mullen C, Flores A, Grineski S, Collins T. Exploring the distributional environmental justice implications of an air quality monitoring network in Los Angeles County. Environ Res. 2022;206:112612.
235. Mullen C, Grineski S, Collins T, Xing W, Whitaker R, Sayahi T, et al. Patterns of distributive environmental inequity under different PM(2.5) air pollution scenarios for Salt Lake County public schools. Environ Res. 2020;186:109543.
236. Muller MHB, Polder A, Brynildsrud OB, Gronnestad R, Karimi M, Lie E, et al. Prenatal exposure to persistent organic pollutants in Northern Tanzania and their distribution between breast milk, maternal blood, placenta and cord blood. Environ Res. 2019;170:433-42.
237. Munoz-Pizza DM, Villada-Canela M, Reyna MA, Texcalac-Sangrador JL, Serrano-Lomelin J, Osornio-Vargas A. Assessing the Influence of Socioeconomic Status and Air Pollution Levels on the Public Perception of Local Air Quality in a Mexico-US Border City. Int J Environ Res Public Health. 2020;17(13).
238. Murage P, Hajat S, Bone A. Variation in Cold-Related Mortality in England Since the Introduction of the Cold Weather Plan: Which Areas Have the Greatest Unmet Needs? Int J Environ Res Public Health. 2018;15(11).
239. Murray J, Eskenazi B, Bornman R, Gaspar FW, Crause M, Obida M, et al. Exposure to DDT and hypertensive disorders of pregnancy among South African women from an indoor residual spraying region: The VHEMBE study. Environ Res. 2018;162:49-54.
240. Nadybal SM, Collins TW, Grineski SE. Light pollution inequities in the continental United States: A distributive environmental justice analysis. Environ Res. 2020;189:109959.
241. Nardone A, Rudolph K, Morello-Frosch R, Casey J. Redlines and greenspace: The relationship between historical redlining and 2010 greenspace across the United States. Environ Health Perspect. 2020;129(1):17006.
242. Nguyen VK, Kahana A, Heidt J, Polemi K, Kvasnicka J, Jolliet O, et al. A comprehensive analysis of racial disparities in chemical biomarker concentrations in United States women, 1999-2014. Environ Int. 2020;137:105496.
243. Niehoff NM, O'Brien KM, Keil AP, Levine KE, Liyanapatirana C, Haines LG, et al. Metals and Breast Cancer Risk: A Prospective Study Using Toenail Biomarkers. Am J Epidemiol. 2021;190(11):2360-73.
244. Nigra AE, Chen Q, Chillrud SN, Wang L, Harvey D, Mailloux B, et al. Inequalities in Public Water Arsenic Concentrations in Counties and Community Water Systems across the United States, 2006-2011. Environ Health Perspect. 2020;128(12):127001.
245. Nolan JES, Coker ES, Ward BR, Williamson YA, Harley KG. "Freedom to Breathe": Youth Participatory Action Research (YPAR) to Investigate Air Pollution Inequities in Richmond, CA. Int J Environ Res Public Health. 2021;18(2).
246. North CM, MacNaughton P, Lai PS, Vallarino J, Okello S, Kakuhikire B, et al. Personal carbon monoxide exposure, respiratory symptoms, and the potentially modifying roles of sex and HIV infection in rural Uganda: a cohort study. Environ Health. 2019;18(1):73.
247. Nozadi SS, Li L, Luo L, MacKenzie D, Erdei E, Du R, et al. Prenatal Metal Exposures and Infants' Developmental Outcomes in a Navajo Population. Int J Environ Res Public Health. 2021;19(1).
248. O'Regan AC, Hunter RF, Nyhan MM. "Biophilic Cities": Quantifying the Impact of Google Street View-Derived Greenspace Exposures on Socioeconomic Factors and Self-Reported Health. Environ Sci Technol. 2021;55(13):9063-73.
249. O'Shea MJ, Toupal J, Caballero-Gomez H, McKeon TP, Howarth MV, Pepino R, et al. Lead Pollution, Demographics, and Environmental Health Risks: The Case of Philadelphia, USA. Int J Environ Res Public Health. 2021;18(17).
250. Obeng-Gyasi E, Roostaei J, Gibson JM. Lead Distribution in Urban Soil in a Medium-Sized City: Household-Scale Analysis. Environ Sci Technol. 2021;55(6):3696-705.
251. Odetola L, Sills S, Morrison S. A pilot study on the feasibility of testing residential tap water in North Carolina: implications for environmental justice and health. J Expo Sci Environ Epidemiol. 2021;31(6):972-8.
252. Ojeda AS, Widener J, Aston CE, Philp RP. ESRD and ESRD-DM associated with lignite-containing aquifers in the U.S. Gulf Coast region of Arkansas, Louisiana, and Texas. Int J Hyg Environ Health. 2018;221(6):958-66.
253. Okello G, Devereux G, Semple S. Women and girls in resource poor countries experience much greater exposure to household air pollutants than men: Results from Uganda and Ethiopia. Environ Int. 2018;119:429-37.
254. Okorie CN, Thomas MD, Mendez RM, Di Giuseppe EC, Roberts NS, Marquez-Magana L. Geospatial Distributions of Lead Levels Found in Human Hair and Preterm Birth in San Francisco Neighborhoods. Int J Environ Res Public Health. 2021;19(1).
255. Orta OR, Wesselink AK, Bethea TN, Claus Henn B, Weuve J, Fruh V, et al. Brominated flame retardants and organochlorine pesticides and incidence of uterine leiomyomata: A prospective ultrasound study. Environ Epidemiol. 2021;5(1):e127.
256. Ouyang W, Gao B, Cheng H, Hao Z, Wu N. Exposure inequality assessment for PM(2.5) and the potential association with environmental health in Beijing. Sci Total Environ. 2018;635:769-78.
257. Padula AM, Huang H, Baer RJ, August LM, Jankowska MM, Jellife-Pawlowski LL, et al. Environmental pollution and social factors as contributors to preterm birth in Fresno County. Environ Health. 2018;17(1):70.
258. Padula AM, Ma C, Huang H, Morello-Frosch R, Woodruff TJ, Carmichael SL. Drinking water contaminants in California and hypertensive disorders in pregnancy. Environ Epidemiol. 2021;5(2):e149.
259. Papatheodorou S, Yao W, Vieira CLZ, Li L, Wylie BJ, Schwartz J, et al. Residential radon exposure and hypertensive disorders of pregnancy in Massachusetts, USA: A cohort study. Environ Int. 2021;146:106285.
260. Parada H, Jr., Wu T, Fry RC, Farnan L, Smith GJ, Mohler JL, et al. Understanding the Relationship between Environmental Arsenic and Prostate Cancer Aggressiveness among African-American and European-American Men in North Carolina. Int J Environ Res Public Health. 2020;17(22).
261. Park C, Hwang M, Baek Y, Jung S, Lee Y, Paek D, et al. Urinary phthalate metabolite and bisphenol A levels in the Korean adult population in association with sociodemographic and behavioral characteristics: Korean National Environmental Health Survey (KoNEHS) 2012-2014. Int J Hyg Environ Health. 2019;222(5):903-10.
262. Park SK, Peng Q, Ding N, Mukherjee B, Harlow SD. Determinants of per- and polyfluoroalkyl substances (PFAS) in midlife women: Evidence of racial/ethnic and geographic differences in PFAS exposure. Environ Res. 2019;175:186-99.
263. Park YM, Kwan MP. Understanding Racial Disparities in Exposure to Traffic-Related Air Pollution: Considering the Spatiotemporal Dynamics of Population Distribution. Int J Environ Res Public Health. 2020;17(3).
264. Pearson D, Basu R, Wu XM, Ebisu K. Temperature and hand, foot and mouth disease in California: An exploratory analysis of emergency department visits by season, 2005-2013. Environ Res. 2020;185:109461.
265. Perera FP, Wheelock K, Wang Y, Tang D, Margolis AE, Badia G, et al. Combined effects of prenatal exposure to polycyclic aromatic hydrocarbons and material hardship on child ADHD behavior problems. Environ Res. 2018;160:506-13.
266. Persson A, Moller J, Engstrom K, Sundstrom ML, Nooijen CFJ. Is moving to a greener or less green area followed by changes in physical activity? Health Place. 2019;57:165-70.
267. Philips EM, Jaddoe VWV, Asimakopoulos AG, Kannan K, Steegers EAP, Santos S, et al. Bisphenol and phthalate concentrations and its determinants among pregnant women in a population-based cohort in the Netherlands, 2004-5. Environ Res. 2018;161:562-72.
268. Polinski KJ, Dabelea D, Hamman RF, Adgate JL, Calafat AM, Ye X, et al. Distribution and predictors of urinary concentrations of phthalate metabolites and phenols among pregnant women in the Healthy Start Study. Environ Res. 2018;162:308-17.
269. Pope CA, 3rd, Lefler JS, Ezzati M, Higbee JD, Marshall JD, Kim SY, et al. Mortality Risk and Fine Particulate Air Pollution in a Large, Representative Cohort of U.S. Adults. Environ Health Perspect. 2019;127(7):77007.
270. Preston EV, Chan M, Nozhenko K, Bellavia A, Grenon MC, Cantonwine DE, et al. Socioeconomic and racial/ethnic differences in use of endocrine-disrupting chemical-associated personal care product categories among pregnant women. Environ Res. 2021;198:111212.
271. Preston EV, Fruh V, Quinn MR, Hacker MR, Wylie BJ, O'Brien K, et al. Endocrine disrupting chemical-associated hair product use during pregnancy and gestational age at delivery: a pilot study. Environ Health. 2021;20(1):86.
272. Protano C, Cammalleri V, Antonucci A, Ungureanu AS, Santilli F, Martellucci S, et al. Further Insights on Predictors of Environmental Tobacco Smoke Exposure during the Pediatric Age. Int J Environ Res Public Health. 2019;16(21).
273. Puett RC, Yanosky JD, Mittleman MA, Montresor-Lopez J, Bell RA, Crume TL, et al. Inflammation and acute traffic-related air pollution exposures among a cohort of youth with type 1 diabetes. Environ Int. 2019;132:105064.
274. Pun VC, Manjourides J, Suh HH. Association of neighborhood greenness with self-perceived stress, depression and anxiety symptoms in older U.S adults. Environ Health. 2018;17(1):39.
275. Qiu X, Fong KC, Shi L, Papatheodorou S, Di Q, Just A, et al. Prenatal exposure to particulate air pollution and gestational age at delivery in Massachusetts neonates 2001-2015: A perspective of causal modeling and health disparities. Environ Epidemiol. 2020;4(5):e113.
276. Quandt SA, Mora DC, Seering TL, Chen H, Arcury TA, Laurienti PJ. Using Life History Calendars to Estimate in Utero and Early Life Pesticide Exposure of Latinx Children in Farmworker Families. Int J Environ Res Public Health. 2020;17(10).
277. Rafiepourgatabi M, Woodward A, Salmond JA, Dirks KN. Socioeconomic Status and Route Characteristics in Relation to Children's Exposure to Air Pollution from Road Traffic While Walking to School in Auckland, New Zealand. Int J Environ Res Public Health. 2021;18(9).
278. Rajkumar S, Young BN, Clark ML, Benka-Coker ML, Bachand AM, Brook RD, et al. Household air pollution from biomass-burning cookstoves and metabolic syndrome, blood lipid concentrations, and waist circumference in Honduran women: A cross-sectional study. Environ Res. 2019;170:46-55.
279. Rammah A, Whitworth KW, Han I, Chan W, Hess JW, Symanski E. Temperature, placental abruption and stillbirth. Environ Int. 2019;131:105067.
280. Rammah A, Whitworth KW, Han I, Chan W, Symanski E. Time-Varying Exposure to Ozone and Risk of Stillbirth in a Nonattainment Urban Region. Am J Epidemiol. 2019;188(7):1288-95.
281. Ramphal B, Dworkin JD, Pagliaccio D, Margolis AE. Noise complaint patterns in New York City from January 2010 through February 2021: Socioeconomic disparities and COVID-19 exacerbations. Environ Res. 2022;206:112254.
282. Rana J, Uddin J, Peltier R, Oulhote Y. Associations between Indoor Air Pollution and Acute Respiratory Infections among Under-Five Children in Afghanistan: Do SES and Sex Matter? Int J Environ Res Public Health. 2019;16(16).
283. Ravenscroft J, Schell LM, Akwesasne Task Force on the E. Patterns of PCB exposure among Akwesasne adolescents: The role of dietary and inhalation pathways. Environ Int. 2018;121(Pt 1):963-72.
284. Reames TG, Bravo MA. People, place and pollution: Investigating relationships between air quality perceptions, health concerns, exposure, and individual- and area-level characteristics. Environ Int. 2019;122:244-55.
285. Reeves KW, Santana MD, Manson JE, Hankinson SE, Zoeller RT, Bigelow C, et al. Predictors of urinary phthalate biomarker concentrations in postmenopausal women. Environ Res. 2019;169:122-30.
286. Rehling J, Bunge C, Waldhauer J, Conrad A. Socioeconomic Differences in Walking Time of Children and Adolescents to Public Green Spaces in Urban Areas-Results of the German Environmental Survey (2014-2017). Int J Environ Res Public Health. 2021;18(5).
287. Renteria R, Grineski S, Collins T, Flores A, Trego S. Social disparities in neighborhood heat in the Northeast United States. Environ Res. 2022;203:111805.
288. Reynolds P, Canchola AJ, Duffy CN, Hurley S, Neuhausen SL, Horn-Ross PL, et al. Urinary cadmium and timing of menarche and pubertal development in girls. Environ Res. 2020;183:109224.
289. Rhee J, Fabian MP, Ettinger de Cuba S, Coleman S, Sandel M, Lane KJ, et al. Effects of Maternal Homelessness, Supplemental Nutrition Programs, and Prenatal PM(2.5) on Birthweight. Int J Environ Res Public Health. 2019;16(21).
290. Richmond-Bryant J, Mikati I, Benson AF, Luben TJ, Sacks JD. Disparities in Distribution of Particulate Matter Emissions from US Coal-Fired Power Plants by Race and Poverty Status After Accounting for Reductions in Operations Between 2015 and 2017. Am J Public Health. 2020;110(5):655-61.
291. Riddell CA, Goin DE, Morello-Frosch R, Apte JS, Glymour MM, Torres JM, et al. Hyper-localized measures of air pollution and risk of preterm birth in Oakland and San Jose, California. Int J Epidemiol. 2022;50(6):1875-85.
292. Roberman J, Emeto TI, Adegboye OA. Adverse Birth Outcomes Due to Exposure to Household Air Pollution from Unclean Cooking Fuel among Women of Reproductive Age in Nigeria. Int J Environ Res Public Health. 2021;18(2).
293. Robinson O, Tamayo I, de Castro M, Valentin A, Giorgis-Allemand L, Hjertager Krog N, et al. The Urban Exposome during Pregnancy and Its Socioeconomic Determinants. Environ Health Perspect. 2018;126(7):077005.
294. Rosofsky A, Levy JI, Breen MS, Zanobetti A, Fabian MP. The impact of air exchange rate on ambient air pollution exposure and inequalities across all residential parcels in Massachusetts. J Expo Sci Environ Epidemiol. 2019;29(4):520-30.
295. Rosofsky A, Levy JI, Zanobetti A, Janulewicz P, Fabian MP. Temporal trends in air pollution exposure inequality in Massachusetts. Environ Res. 2018;161:76-86.
296. Rowles Iii LS, Hossain AI, Ramirez I, Durst NJ, Ward PM, Kirisits MJ, et al. Seasonal contamination of well-water in flood-prone colonias and other unincorporated U.S. communities. Sci Total Environ. 2020;740:140111.
297. Runkle JD, Matthews JL, Sparks L, McNicholas L, Sugg MM. Racial and ethnic disparities in pregnancy complications and the protective role of greenspace: A retrospective birth cohort study. Sci Total Environ. 2022;808:152145.
298. Saez M, Lopez-Casasnovas G. Assessing the Effects on Health Inequalities of Differential Exposure and Differential Susceptibility of Air Pollution and Environmental Noise in Barcelona, 2007-2014. Int J Environ Res Public Health. 2019;16(18).
299. Sampson NR, Price CE, Kassem J, Doan J, Hussein J. "We're Just Sitting Ducks": Recurrent Household Flooding as An Underreported Environmental Health Threat in Detroit's Changing Climate. Int J Environ Res Public Health. 2018;16(1).
300. Sanchez M, Mila C, Sreekanth V, Balakrishnan K, Sambandam S, Nieuwenhuijsen M, et al. Personal exposure to particulate matter in peri-urban India: predictors and association with ambient concentration at residence. J Expo Sci Environ Epidemiol. 2020;30(4):596-605.
301. Saucy A, Roosli M, Kunzli N, Tsai MY, Sieber C, Olaniyan T, et al. Land Use Regression Modelling of Outdoor NO(2) and PM(2.5) Concentrations in Three Low Income Areas in the Western Cape Province, South Africa. Int J Environ Res Public Health. 2018;15(7).
302. Schaider LA, Swetschinski L, Campbell C, Rudel RA. Environmental justice and drinking water quality: are there socioeconomic disparities in nitrate levels in U.S. drinking water? Environ Health. 2019;18(1):3.
303. Schildroth S, Wise LA, Wesselink AK, De La Cruz P, Bethea TN, Weuve J, et al. Correlates of Persistent Endocrine-Disrupting Chemical Mixtures among Reproductive-Aged Black Women. Environ Sci Technol. 2021;55(20):14000-14.
304. Schilmann A, Riojas-Rodriguez H, Catalan-Vazquez M, Estevez-Garcia JA, Masera O, Berrueta-Soriano V, et al. A follow-up study after an improved cookstove intervention in rural Mexico: Estimation of household energy use and chronic PM(2.5) exposure. Environ Int. 2019;131:105013.
305. Schule SA, Nanninga S, Dreger S, Bolte G. Relations between Objective and Perceived Built Environments and the Modifying Role of Individual Socioeconomic Position. A Cross-Sectional Study on Traffic Noise and Urban Green Space in a Large German City. Int J Environ Res Public Health. 2018;15(8).
306. Schwarz L, Bruckner T, Ilango SD, Sheridan P, Basu R, Benmarhnia T. A quantile regression approach to examine fine particles, term low birth weight, and racial/ethnic disparities. Environ Epidemiol. 2019;3(4):e060.
307. Shaffer RM, Ferguson KK, Sheppard L, James-Todd T, Butts S, Chandrasekaran S, et al. Maternal urinary phthalate metabolites in relation to gestational diabetes and glucose intolerance during pregnancy. Environ Int. 2019;123:588-96.
308. Sheffield PE, Shmool JLC, Kinnee EJ, Clougherty JE. Violent crime and socioeconomic deprivation in shaping asthma-related pollution susceptibility: a case-crossover design. J Epidemiol Community Health. 2019;73(9):846-53.
309. Sheridan CE, Roscoe CJ, Gulliver J, de Preux L, Fecht D. Inequalities in Exposure to Nitrogen Dioxide in Parks and Playgrounds in Greater London. Int J Environ Res Public Health. 2019;16(17).
310. Sheridan P, Ilango S, Bruckner TA, Wang Q, Basu R, Benmarhnia T. Ambient Fine Particulate Matter and Preterm Birth in California: Identification of Critical Exposure Windows. Am J Epidemiol. 2019;188(9):1608-15.
311. Shi T, Liu M, Hu Y, Li C, Zhang C, Ren B. Spatiotemporal Pattern of Fine Particulate Matter and Impact of Urban Socioeconomic Factors in China. Int J Environ Res Public Health. 2019;16(7).
312. Shrestha PM, Humphrey JL, Carlton EJ, Adgate JL, Barton KE, Root ED, et al. Impact of Outdoor Air Pollution on Indoor Air Quality in Low-Income Homes during Wildfire Seasons. Int J Environ Res Public Health. 2019;16(19).
313. Silva GS, Warren JL, Deziel NC. Spatial Modeling to Identify Sociodemographic Predictors of Hydraulic Fracturing Wastewater Injection Wells in Ohio Census Block Groups. Environ Health Perspect. 2018;126(6):067008.
314. Silva MJ, Wong LY, Samandar E, Preau JL, Jr., Jia LT, Calafat AM. Exposure to di-2-ethylhexyl terephthalate in the U.S. general population from the 2015-2016 National Health and Nutrition Examination Survey. Environ Int. 2019;123:141-7.
315. Sim K, Kim Y, Hashizume M, Gasparrini A, Armstrong B, Sera F, et al. Nonlinear temperature-suicide association in Japan from 1972 to 2015: Its heterogeneity and the role of climate, demographic, and socioeconomic factors. Environ Int. 2020;142:105829.
316. Smith ML, Hardeman RR. Association of Summer Heat Waves and the Probability of Preterm Birth in Minnesota: An Exploration of the Intersection of Race and Education. Int J Environ Res Public Health. 2020;17(17).
317. Sobel M, Navas-Acien A, Powers M, Grau-Perez M, Goessler W, Best LG, et al. Environmental-level exposure to metals and metal-mixtures associated with spirometry-defined lung disease in American Indian adults: Evidence from the Strong Heart Study. Environ Res. 2022;207:112194.
318. Sohrabi S, Zietsman J, Khreis H. Burden of Disease Assessment of Ambient Air Pollution and Premature Mortality in Urban Areas: The Role of Socioeconomic Status and Transportation. Int J Environ Res Public Health. 2020;17(4).
319. Son JY, Lane KJ, Miranda ML, Bell ML. Health disparities attributable to air pollutant exposure in North Carolina: Influence of residential environmental and social factors. Health Place. 2020;62:102287.
320. Son JY, Lee JT, Lane KJ, Bell ML. Impacts of high temperature on adverse birth outcomes in Seoul, Korea: Disparities by individual- and community-level characteristics. Environ Res. 2019;168:460-6.
321. Son JY, Sabath MB, Lane KJ, Miranda ML, Dominici F, Di Q, et al. Long-term Exposure to PM2.5 and Mortality for the Older Population: Effect Modification by Residential Greenness. Epidemiology. 2021;32(4):477-86.
322. Song L, Smith GS, Adar SD, Post WS, Guallar E, Navas-Acien A, et al. Ambient air pollution as a mediator in the pathway linking race/ethnicity to blood pressure elevation: The multi-ethnic study of atherosclerosis (MESA). Environ Res. 2020;180:108776.
323. Sterrett ME, Bloom MS, Jamro EL, Wenzel AG, Wineland RJ, Unal ER, et al. Maternal Food and Beverage Consumption Behaviors and Discrepant Phthalate Exposure by Race. Int J Environ Res Public Health. 2021;18(4).
324. Su B, Wu L. Occupants' Health and Their Living Conditions of Remote Indigenous Communities in New Zealand. Int J Environ Res Public Health. 2020;17(22).
325. Sun S, Weinberger KR, Yan M, Brooke Anderson G, Wellenius GA. Tropical cyclones and risk of preterm birth: A retrospective analysis of 20 million births across 378 US counties. Environ Int. 2020;140:105825.
326. Sun X, Li X, Liu D, Yang T, Zhao Y, Wu T, et al. Use of a Survey to Assess the Environmental Exposure and Family Perception to Lead in Children (<6 Years) in Four Valley Cities, Northwestern China. Int J Environ Res Public Health. 2018;15(4).
327. Sun Y, Li X, Benmarhnia T, Chen JC, Avila C, Sacks DA, et al. Exposure to air pollutant mixture and gestational diabetes mellitus in Southern California: Results from electronic health record data of a large pregnancy cohort. Environ Int. 2022;158:106888.
328. Symanski E, An Han H, Hopkins L, Smith MA, McCurdy S, Han I, et al. Metal air pollution partnership solutions: building an academic-government-community-industry collaboration to improve air quality and health in environmental justice communities in Houston. Environ Health. 2020;19(1):39.
329. Tamire M, Addissie A, Kumie A, Husmark E, Skovbjerg S, Andersson R, et al. Respiratory Symptoms and Lung Function among Ethiopian Women in Relation to Household Fuel Use. Int J Environ Res Public Health. 2019;17(1).
330. Tanzer R, Malings C, Hauryliuk A, Subramanian R, Presto AA. Demonstration of a Low-Cost Multi-Pollutant Network to Quantify Intra-Urban Spatial Variations in Air Pollutant Source Impacts and to Evaluate Environmental Justice. Int J Environ Res Public Health. 2019;16(14).
331. Tapia V, Steenland K, Sarnat SE, Vu B, Liu Y, Sanchez-Ccoyllo O, et al. Time-series analysis of ambient PM(2.5) and cardiorespiratory emergency room visits in Lima, Peru during 2010-2016. J Expo Sci Environ Epidemiol. 2020;30(4):680-8.
332. Thacher JD, Poulsen AH, Raaschou-Nielsen O, Jensen A, Hillig K, Roswall N, et al. High-resolution assessment of road traffic noise exposure in Denmark. Environ Res. 2020;182:109051.
333. Thind MPS, Tessum CW, Azevedo IL, Marshall JD. Fine Particulate Air Pollution from Electricity Generation in the US: Health Impacts by Race, Income, and Geography. Environ Sci Technol. 2019;53(23):14010-9.
334. Tonne C, Mila C, Fecht D, Alvarez M, Gulliver J, Smith J, et al. Socioeconomic and ethnic inequalities in exposure to air and noise pollution in London. Environ Int. 2018;115:170-9.
335. Torres Toda M, Miri M, Alonso L, Gomez-Roig MD, Foraster M, Dadvand P. Exposure to greenspace and birth weight in a middle-income country. Environ Res. 2020;189:109866.
336. Tsai K, Simiyu S, Mumma J, Aseyo RE, Cumming O, Dreibelbis R, et al. Enteric Pathogen Diversity in Infant Foods in Low-Income Neighborhoods of Kisumu, Kenya. Int J Environ Res Public Health. 2019;16(3).
337. Tu R, Hou J, Liu X, Li R, Dong X, Pan M, et al. Low socioeconomic status aggravated associations of exposure to mixture of air pollutants with obesity in rural Chinese adults: A cross-sectional study. Environ Res. 2021;194:110632.
338. Uche UI, Evans S, Rundquist S, Campbell C, Naidenko OV. Community-Level Analysis of Drinking Water Data Highlights the Importance of Drinking Water Metrics for the State, Federal Environmental Health Justice Priorities in the United States. Int J Environ Res Public Health. 2021;18(19).
339. Van Horne YO, Alcala CS, Peltier RE, Quintana PJE, Seto E, Gonzales M, et al. An applied environmental justice framework for exposure science. J Expo Sci Environ Epidemiol. 2022.
340. Van Vliet EDS, Kinney PL, Owusu-Agyei S, Schluger NW, Ae-Ngibise KA, Whyatt RM, et al. Current respiratory symptoms and risk factors in pregnant women cooking with biomass fuels in rural Ghana. Environ Int. 2019;124:533-40.
341. van Woerden I, Bruening M, Montresor-Lopez J, Payne-Sturges DC. Trends and disparities in urinary BPA concentrations among U.S. emerging adults. Environ Res. 2019;176:108515.
342. Varnell RR, Arnold TJ, Quandt SA, Talton JW, Chen H, Miles CM, et al. Menstrual Cycle Patterns and Irregularities in Hired Latinx Child Farmworkers. J Occup Environ Med. 2021;63(1):38-43.
343. Varshavsky JR, Morello-Frosch R, Harwani S, Snider M, Petropoulou SE, Park JS, et al. A Pilot Biomonitoring Study of Cumulative Phthalates Exposure among Vietnamese American Nail Salon Workers. Int J Environ Res Public Health. 2020;17(1).
344. Veluswami Subramanian S, Cho MJ, Mukhitdinova F. Health Risk in Urbanizing Regions: Examining the Nexus of Infrastructure, Hygiene and Health in Tashkent Province, Uzbekistan. Int J Environ Res Public Health. 2018;15(11).
345. Vernet C, Johnson M, Kogut K, Hyland C, Deardorff J, Bradman A, et al. Organophosphate pesticide exposure during pregnancy and childhood and onset of juvenile delinquency by age 16 years: The CHAMACOS cohort. Environ Res. 2021;197:111055.
346. Voelkel J, Hellman D, Sakuma R, Shandas V. Assessing Vulnerability to Urban Heat: A Study of Disproportionate Heat Exposure and Access to Refuge by Socio-Demographic Status in Portland, Oregon. Int J Environ Res Public Health. 2018;15(4).
347. Vowles M, Kerry R, Ingram B, Mason L. Investigation of the Environmental and Socio-Economic Characteristics of Counties with a High Asthma Burden to Focus Asthma Action in Utah. Int J Environ Res Public Health. 2020;17(14).
348. Wang F, Yang J, Shackman J, Liu X. Impact of Income Inequality on Urban Air Quality: A Game Theoretical and Empirical Study in China. Int J Environ Res Public Health. 2021;18(16).
349. Wang J, Kuffer M, Sliuzas R, Kohli D. The exposure of slums to high temperature: Morphology-based local scale thermal patterns. Sci Total Environ. 2019;650(Pt 2):1805-17.
350. Wang L, Hou J, Hu C, Zhou Y, Sun H, Zhang J, et al. Mediating factors explaining the associations between polycyclic aromatic hydrocarbons exposure, low socioeconomic status and diabetes: A structural equation modeling approach. Sci Total Environ. 2019;648:1476-83.
351. Wang Q, Zhang Y, Ban J, Zhu H, Xu H, Li T. The relationship between population heat vulnerability and urbanization levels: A county-level modeling study across China. Environ Int. 2021;156:106742.
352. Wang R, Xue D, Liu Y, Liu P, Chen H. The Relationship between Air Pollution and Depression in China: Is Neighbourhood Social Capital Protective? Int J Environ Res Public Health. 2018;15(6).
353. Wang VA, Chu MT, Chie L, Gaston SA, Jackson CL, Newendorp N, et al. Acculturation and endocrine disrupting chemical-associated personal care product use among US-based foreign-born Chinese women of reproductive age. J Expo Sci Environ Epidemiol. 2021;31(2):224-32.
354. Wang X, Mukherjee B, Batterman S, Harlow SD, Park SK. Urinary metals and metal mixtures in midlife women: The Study of Women's Health Across the Nation (SWAN). Int J Hyg Environ Health. 2019;222(5):778-89.
355. Wang Y, Fan H, Banerjee R, Weaver AM, Weiner M. A National County-Level Assessment of U.S. Nursing Facility Characteristics Associated with Long-Term Exposure to Traffic Pollution in Older Adults. Int J Environ Res Public Health. 2018;15(3).
356. Wang YX, Liu C, Chen YJ, Chen HG, Yang P, Wang P, et al. Predictors and correlations of phthalate metabolite concentrations in urine and seminal plasma among reproductive-aged men. Environ Res. 2018;161:336-44.
357. Warner GR, Pacyga DC, Strakovsky RS, Smith R, James-Todd T, Williams PL, et al. Urinary phthalate metabolite concentrations and hot flashes in women from an urban convenience sample of midlife women. Environ Res. 2021;197:110891.
358. Wasserman GA, Liu X, Parvez F, Chen Y, Factor-Litvak P, LoIacono NJ, et al. A cross-sectional study of water arsenic exposure and intellectual function in adolescence in Araihazar, Bangladesh. Environ Int. 2018;118:304-13.
359. Wattigney WA, Savadatti SS, Liu M, Pavuk M, Lewis-Michl E, Kannan K, et al. Biomonitoring of per- and polyfluoroalkyl substances in minority angler communities in central New York State. Environ Res. 2022;204(Pt C):112309.
360. Weinstein JR, Diaz-Artiga A, Benowitz N, Thompson LM. Reductions in urinary metabolites of exposure to household air pollution in pregnant, rural Guatemalan women provided liquefied petroleum gas stoves. J Expo Sci Environ Epidemiol. 2020;30(2):362-73.
361. Wesselink AK, Bethea TN, McClean M, Weuve J, Williams PL, Hauser R, et al. Predictors of plasma polychlorinated biphenyl concentrations among reproductive-aged black women. Int J Hyg Environ Health. 2019;222(7):1001-10.
362. Wesselink AK, Fruh V, Hauser R, Weuve J, Taylor KW, Orta OR, et al. Correlates of urinary concentrations of phthalate and phthalate alternative metabolites among reproductive-aged Black women from Detroit, Michigan. J Expo Sci Environ Epidemiol. 2021;31(3):461-75.
363. Wheeler DC, Boyle J, Nelson EJ. Modeling annual elevated blood lead levels among children in Maryland in relation to neighborhood deprivation. Sci Total Environ. 2022;805:150333.
364. Wheeler DC, Jones RM, Schootman M, Nelson EJ. Explaining variation in elevated blood lead levels among children in Minnesota using neighborhood socioeconomic variables. Sci Total Environ. 2019;650(Pt 1):970-7.
365. Williams AA, Spengler JD, Catalano P, Allen JG, Cedeno-Laurent JG. Building Vulnerability in a Changing Climate: Indoor Temperature Exposures and Health Outcomes in Older Adults Living in Public Housing during an Extreme Heat Event in Cambridge, MA. Int J Environ Res Public Health. 2019;16(13).
366. Williams AD, Grantz KL, Zhang C, Nobles C, Sherman S, Mendola P. Ambient Volatile Organic Compounds and Racial/Ethnic Disparities in Gestational Diabetes Mellitus: Are Asian/Pacific Islander Women at Greater Risk? Am J Epidemiol. 2019;188(2):389-97.
367. Williams AD, Ha S, Shenassa E, Messer LC, Kanner J, Mendola P. Joint effects of ethnic enclave residence and ambient volatile organic compounds exposure on risk of gestational diabetes mellitus among Asian/Pacific Islander women in the United States. Environ Health. 2021;20(1):56.
368. Windham GC, Soriano JW, Dobraca D, Sosnoff CS, Hiatt RA, Kushi LH. Environmental Tobacco Smoke Exposure in Relation to Family Characteristics, Stressors and Chemical Co-Exposures in California Girls. Int J Environ Res Public Health. 2019;16(21).
369. Winter SC, Obara LM, Barchi F. Environmental Correlates of Health-Related Quality of Life among Women Living in Informal Settlements in Kenya. Int J Environ Res Public Health. 2019;16(20).
370. Wise LA, Wesselink AK, Schildroth S, Calafat AM, Bethea TN, Geller RJ, et al. Correlates of plasma concentrations of per- and poly-fluoroalkyl substances among reproductive-aged Black women. Environ Res. 2022;203:111860.
371. Woo SHL, Liu JC, Yue X, Mickley LJ, Bell ML. Air pollution from wildfires and human health vulnerability in Alaskan communities under climate change. Environ Res Lett. 2020;15(9).
372. Workman B, Beck AF, Newman NC, Nabors L. Evaluation of a Program to Reduce Home Environment Risks for Children with Asthma Residing in Urban Areas. Int J Environ Res Public Health. 2021;19(1).
373. Wortzel JD, Wiebe DJ, Elahi S, Agawu A, Barg FK, Emmett EA. Ascertainment Bias in a Historic Cohort Study of Residents in an Asbestos Manufacturing Community. Int J Environ Res Public Health. 2021;18(5).
374. Wyatt LH, Peterson GCL, Wade TJ, Neas LM, Rappold AG. The contribution of improved air quality to reduced cardiovascular mortality: Declines in socioeconomic differences over time. Environ Int. 2020;136:105430.
375. Xiao C, Yang Y, Xu X, Ma X. Housing Conditions, Neighborhood Physical Environment, and Secondhand Smoke Exposure at Home: Evidence from Chinese Rural-to-Urban Migrant Workers. Int J Environ Res Public Health. 2020;17(8).
376. Xiao Q, Geng G, Liang F, Wang X, Lv Z, Lei Y, et al. Changes in spatial patterns of PM(2.5) pollution in China 2000-2018: Impact of clean air policies. Environ Int. 2020;141:105776.
377. Xie M, Jia C, Zhang Y, Wang B, Qin N, Cao S, et al. Household Exposure to Secondhand Smoke among Chinese Children: Status, Determinants, and Co-Exposures. Int J Environ Res Public Health. 2020;17(15).
378. Xing Q, Sun Z, Tao Y, Zhang X, Miao S, Zheng C, et al. Impacts of urbanization on the temperature-cardiovascular mortality relationship in Beijing, China. Environ Res. 2020;191:110234.
379. Xu J, White AJ, Niehoff NM, O'Brien KM, Sandler DP. Airborne metals exposure and risk of hypertension in the Sister Study. Environ Res. 2020;191:110144.
380. Yan D, Ren X, Zhang W, Li Y, Miao Y. Exploring the real contribution of socioeconomic variation to urban PM(2.5) pollution: New evidence from spatial heteroscedasticity. Sci Total Environ. 2022;806(Pt 4):150929.
381. Yang J, Ma S, Song Y, Li F, Zhou J. Rethinking of Environmental Health Risks: A Systematic Approach of Physical-Social Health Vulnerability Assessment on Heavy-Metal Exposure through Soil and Vegetables. Int J Environ Res Public Health. 2021;18(24).
382. Yang J, Yin P, Sun J, Wang B, Zhou M, Li M, et al. Heatwave and mortality in 31 major Chinese cities: Definition, vulnerability and implications. Sci Total Environ. 2019;649:695-702.
383. Yang Y, Lan H, Li J. Spatial Econometric Analysis of the Impact of Socioeconomic Factors on PM(2.5) Concentration in China's Inland Cities: A Case Study from Chengdu Plain Economic Zone. Int J Environ Res Public Health. 2019;17(1).
384. Yang Y, Li J, Zhu G, Yuan Q. Spatio(-)Temporal Relationship and Evolvement of Socioeconomic Factors and PM(2.5) in China During 1998(-)2016. Int J Environ Res Public Health. 2019;16(7).
385. Yao B, Lu X, Xu L, Wang Y, Qu H, Zhou H. Relationship between low-level lead, cadmium and mercury exposures and blood pressure in children and adolescents aged 8-17 years: An exposure-response analysis of NHANES 2007-2016. Sci Total Environ. 2020;726:138446.
386. Yao L, Huang C, Jing W, Yue X, Xu Y. Quantitative Assessment of Relationship between Population Exposure to PM(2.5) and Socio-Economic Factors at Multiple Spatial Scales over Mainland China. Int J Environ Res Public Health. 2018;15(9).
387. Yao Y, Chen D, Wu Y, Zhou L, Cheng J, Li Y, et al. Urinary phthalate metabolites in primary school starters in Pearl River Delta, China: Occurrences, risks and possible sources. Environ Res. 2019;179(Pt B):108853.
388. Yeter D, Banks EC, Aschner M. Disparity in Risk Factor Severity for Early Childhood Blood Lead among Predominantly African-American Black Children: The 1999 to 2010 US NHANES. Int J Environ Res Public Health. 2020;17(5).
389. Yitshak-Sade M, Fabian MP, Lane KJ, Hart JE, Schwartz JD, Laden F, et al. Estimating the Combined Effects of Natural and Built Environmental Exposures on Birthweight among Urban Residents in Massachusetts. Int J Environ Res Public Health. 2020;17(23).
390. Yitshak-Sade M, James P, Kloog I, Hart JE, Schwartz JD, Laden F, et al. Neighborhood Greenness Attenuates the Adverse Effect of PM(2.5) on Cardiovascular Mortality in Neighborhoods of Lower Socioeconomic Status. Int J Environ Res Public Health. 2019;16(5).
391. Yu S, Zhu X, He Q. An Assessment of Urban Park Access Using House-Level Data in Urban China: Through the Lens of Social Equity. Int J Environ Res Public Health. 2020;17(7).
392. Zandieh R, Martinez J, Flacke J. Older Adults' Outdoor Walking and Inequalities in Neighbourhood Green Spaces Characteristics. Int J Environ Res Public Health. 2019;16(22).
393. Zayas-Costa M, Cole HVS, Anguelovski I, Connolly JJT, Bartoll X, Triguero-Mas M. Mental Health Outcomes in Barcelona: The Interplay between Gentrification and Greenspace. Int J Environ Res Public Health. 2021;18(17).
394. Zhang L, Wu L. Effects of Environmental Quality Perception on Depression: Subjective Social Class as a Mediator. Int J Environ Res Public Health. 2021;18(11).
395. Zhang M, Liu T, Wang G, Buckley JP, Guallar E, Hong X, et al. In Utero Exposure to Heavy Metals and Trace Elements and Childhood Blood Pressure in a U.S. Urban, Low-Income, Minority Birth Cohort. Environ Health Perspect. 2021;129(6):67005.
396. Zhang X, Johnson N, Carrillo G, Xu X. Decreasing trend in passive tobacco smoke exposure and association with asthma in U.S. children. Environ Res. 2018;166:35-41.
397. Zhang X, Zhou S, Lin R, Su L. Relationship between Long-Term Residential Green Exposure and Individuals' Mental Health: Moderated by Income Differences and Residential Location in Urban China. Int J Environ Res Public Health. 2020;17(23).
398. Zhao J, Gladson L, Cromar K. A Novel Environmental Justice Indicator for Managing Local Air Pollution. Int J Environ Res Public Health. 2018;15(6).
399. Zhao X, Cheng H, He S, Cui X, Pu X, Lu L. Spatial associations between social groups and ozone air pollution exposure in the Beijing urban area. Environ Res. 2018;164:173-83.
400. Zhou Y, Buck C, Maier W, von Lengerke T, Walter U, Dreier M. Built Environment and Childhood Weight Status: A Multi-Level Study Using Population-Based Data in the City of Hannover, Germany. Int J Environ Res Public Health. 2020;17(8).
401. Zilversmit Pao L, Harville EW, Wickliffe JK, Shankar A, Buekens P. The Cumulative Risk of Chemical and Nonchemical Exposures on Birth Outcomes in Healthy Women: The Fetal Growth Study. Int J Environ Res Public Health. 2019;16(19).
402. Zottarelli LK, Sharif HO, Xu X, Sunil TS. Effects of social vulnerability and heat index on emergency medical service incidents in San Antonio, Texas, in 2018. J Epidemiol Community Health. 2021;75(3):271-6.
